# π‐Extended 4,5‐Fused Bis‐Fluorene: Highly Open‐Shell Compounds and their Cationic Tetrathiafulvalene Derivatives

**DOI:** 10.1002/anie.202410458

**Published:** 2024-10-21

**Authors:** Frédéric Lirette, Viktor Bliksted Roug Pedersen, Félix Gagnon, Mogens Brøndsted Nielsen, Israel Fernández, Jean‐François Morin

**Affiliations:** ^1^ Department of Chemistry and Centre de Recherche sur les Matériaux Avancés (CERMA) Université Laval 1045 Ave de la Medecine Québec QC Canada G1V0A6; ^2^ Department of Chemistry University of Copenhagen Universitetsparken 5 DK-2100 Copenhagen Ø Denmark; ^3^ Departamento de Química Orgánica and Centro de Innovación en Química Avanzada (ORFEO-CINQA) Facultad de Ciencias Químicas Universidad Complutense de Madrid 28040- Madrid Spain

**Keywords:** Diradical, Aromaticity, Conjugated molecules, Extended fluorene, Open-shell compounds

## Abstract

The synthesis of diradical organic compounds has garnered significant attention due to their thermally accessible spin inversion and optoelectronic properties. Yet, preparing such stable structures with high open‐shell behavior remains challenging. Herein, we report the synthesis and properties of four π‐extended, fused fluorene derivatives with high diradical character, taking advantage of a molecular design where the closed‐shell does not include any Clar sextet, comparatively to a maximum of 5 in the corresponding open‐shell state. This led to an unusual open‐shell triplet ground state with an outstanding singlet‐triplet energy difference (Δ*E*_ST_) of ca. 19 kcal/mol, one of the highest values reported to date for an all‐carbon conjugated scaffold. Incorporation of dithiafulvene units at each end of the molecule (at the five‐membered rings) furnishes extended tetrathiafulvalenes (TTFs) undergoing reversible oxidations to the radical cation and diradical dication. The various pro‐aromatic structures presented herein show highly localized spin density and a limited conjugation due to the confined π‐electrons in the aromatic cycles, as supported by ^1^H NMR, UV/Visible, EPR spectroscopy and DFT calculations.

## Graphical abstract

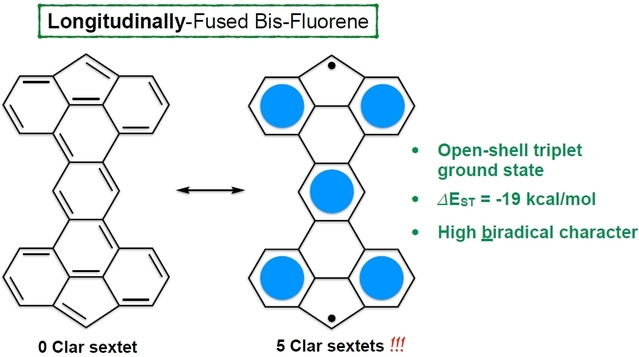

## Introduction

In recent years, there has been a growing interest in organic compounds with high diradical character due to their unique and intriguing electronic properties.[^1^, ^2^, ^3^, ^4^, ^5^] These molecules exist in an equilibrium between a closed‐shell state (*y*=0, all electrons paired) and an open‐shell state (*y*=1, with two or more unpaired electrons).[^6^, ^7^] The presence of open‐shell electronic structures gives rise to a wide range of electronic and magnetic properties that make these molecules attractive candidates for various applications. For example, open‐shell organic molecules can exhibit paramagnetic behavior and spin inversion, which can be exploited in spintronics.[^8^, ^9^, ^10^] In addition, the semiconducting properties of open‐shell compounds can be used in the development of organic electronic devices, such as organic field‐effect transistors (OFETs),[^11^, ^12^, ^13^, ^14^, ^15^] organic light‐emitting diodes (OLEDs),[^16^, ^17^, ^18^, ^19^] and organic photovoltaics (OPVs).[^20^, ^21^, ^22^]

**Figure 1:**
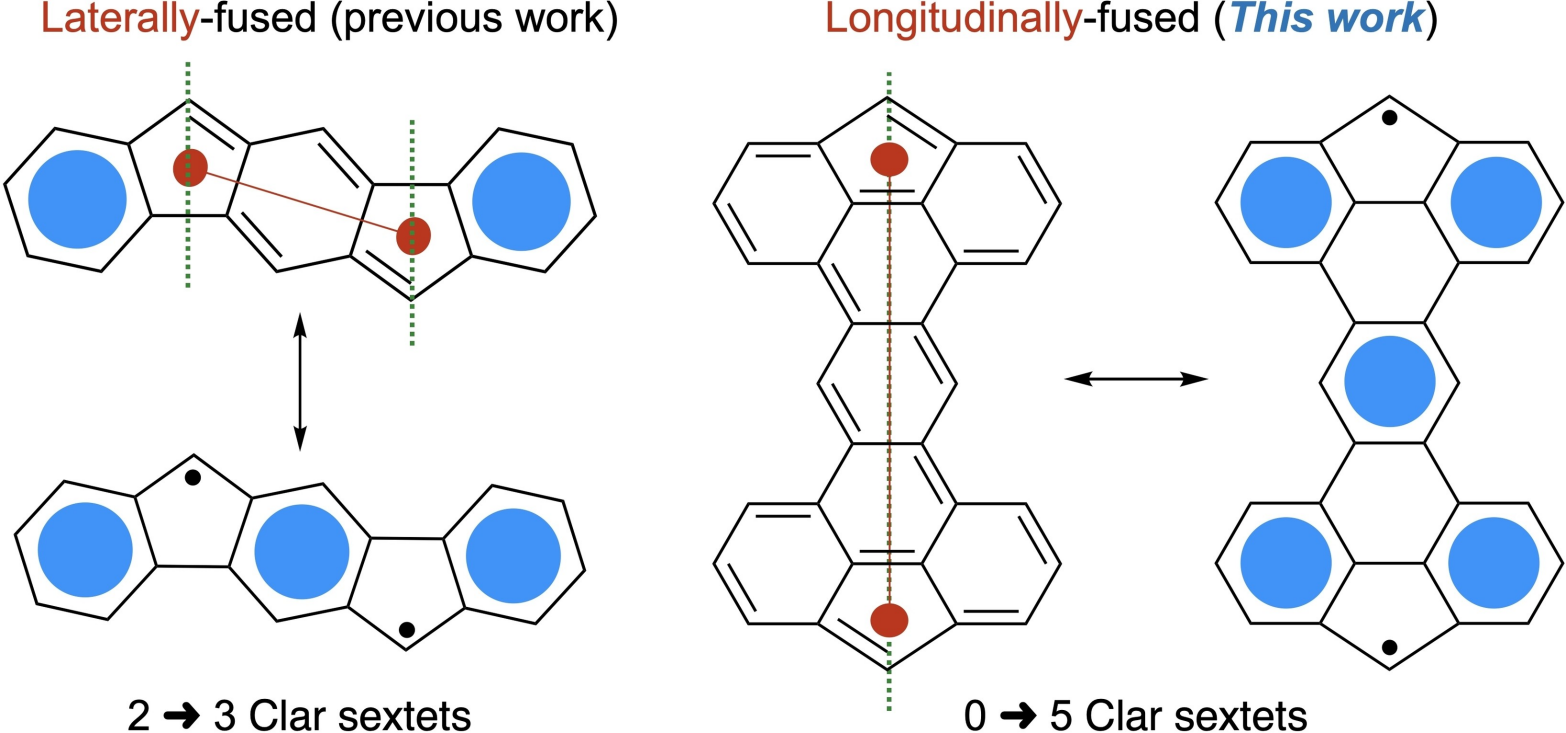
Laterally‐ and longitudinally‐fused 5‐membered rings in their closed‐ and open‐shell forms.

One of the main challenges of this area is the synthesis of stable biradicaloids with an open‐shell character (*y*) as close to 1 as possible. The stability of these molecules is governed by various factors such as the degree of electron delocalization (thermodynamic stability) and steric hindrance around the radical centers (kinetic stability).[^23^, ^24^, ^25^] Indenofluorene‐based open‐shell compounds are one class of molecules that have been extensively studied in the past years due to their high stability and straightforward synthesis.[^15^, ^26^, ^27^, ^28^, ^29^] Their five‐membered rings and pro‐aromatic character drive the localization of the spin density on a kinetically blocked carbon atom, while leading to the formation of additional Clar sextets that stabilize the structures (Figure 1).[^30^, ^31^, ^32^] Although many examples of organic diradicals with a fluorene pattern have been synthesized, most of them are linearly fused with conjugated units on the cycles’ axis (Figure 1, left), meaning that at least one sextet is present in its closed‐shell form.[^33^, ^34^] To form diradical compounds with a *y* value close to 1, a larger difference between the number of Clar sextets in the closed and open‐shell forms is needed.

One possible strategy to optimize the difference in the number of Clar sextets between the closed‐shell and the open‐shell forms is to longitudinally fuse two fluorene units by their 4 and 5 positions through a conjugated bridge (Figure 1, right). In the closed‐shell form, the resulting molecules do not possess any Clar sextet while their open‐shell form possesses 4 or more sextets, which is a major driving force for the formation of the later form. To the best of our knowledge, there are very few reports of diradical compounds that do not possess a Clar sextet in their closed‐shell form without having a *p*‐quinodimethane scaffold.[^14^, ^35^, ^36^, ^37^, ^38^] The Thiele's and Chichibabin's hydrocarbons are the most famous examples of such compounds, but they all used the laterally‐fused strategy and the properties of some analogs remain elusive due to their lack of stability.[^39^, ^40^, ^41^, ^42^]

**Figure 2:**
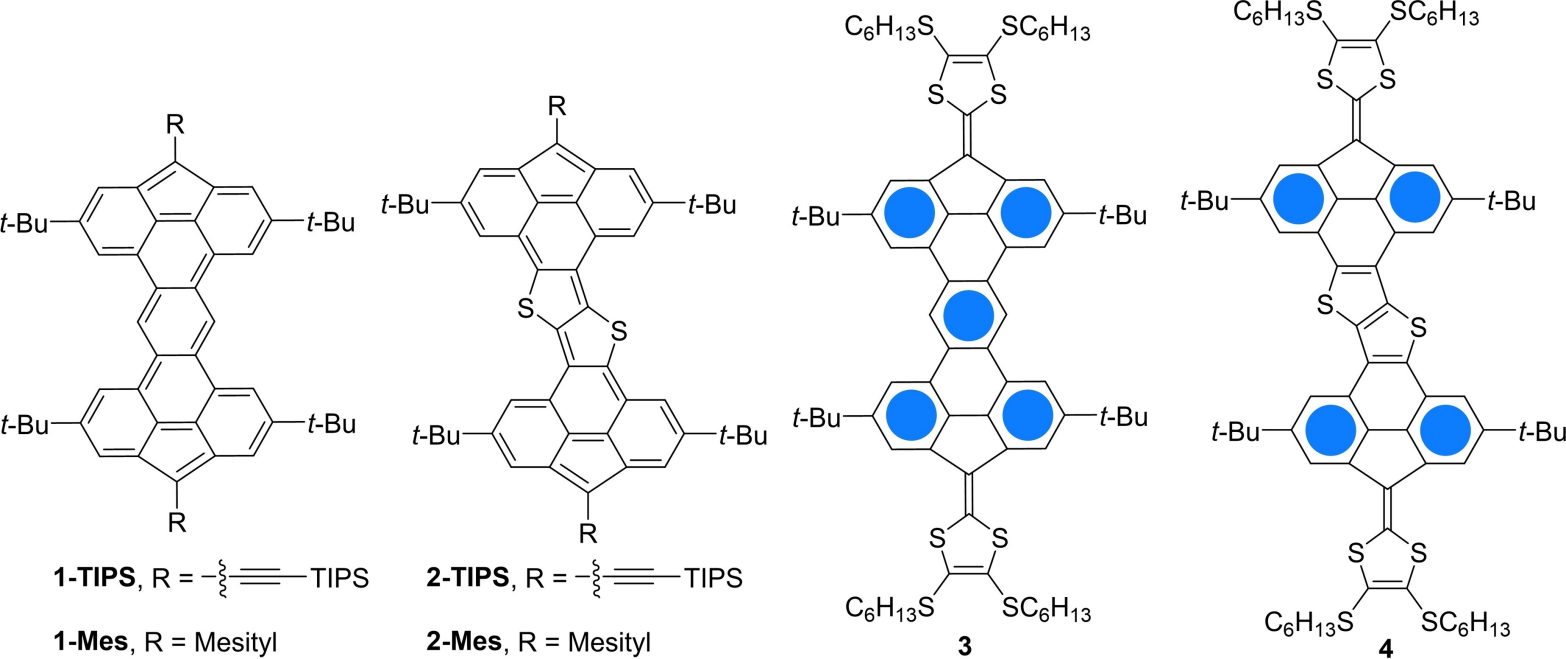
Structures of compounds **1**–**4**.

In this article, we describe the synthesis of a series of π‐extended fused fluorenes possessing an open‐shell character owing to the large difference (up to 5) in the number of Clar sextets between the closed‐ and open‐shell forms. Two π‐conjugated bridges with different electronic properties were used; thus, two 4H‐cyclopenta[*def*]phenanthrenes were fused to either a central benzene core (**1**) or a thieno[3,2‐*b*]thiophene core (**2**), furnishing two fully unsaturated π‐systems that both yielded diradical triplet species (Figure 2). As no electron delocalization of the unpaired electrons is possible without reducing the number of Clar sextets, the spin density is highly localized on the two edge carbon atoms of the five‐membered rings, which is supported by density functional theory (DFT) calculations. Diradical compounds have also been obtained through the double oxidation of extended tetrathiafulvalenes (TTF) (**3** and **4**, Figure 2) whose electrochemical properties have also been studied. All these compounds have been characterized using electron paramagnetic resonance (EPR) and UV/Visible spectroscopy.

## Results and Discussion

**Scheme 1:**
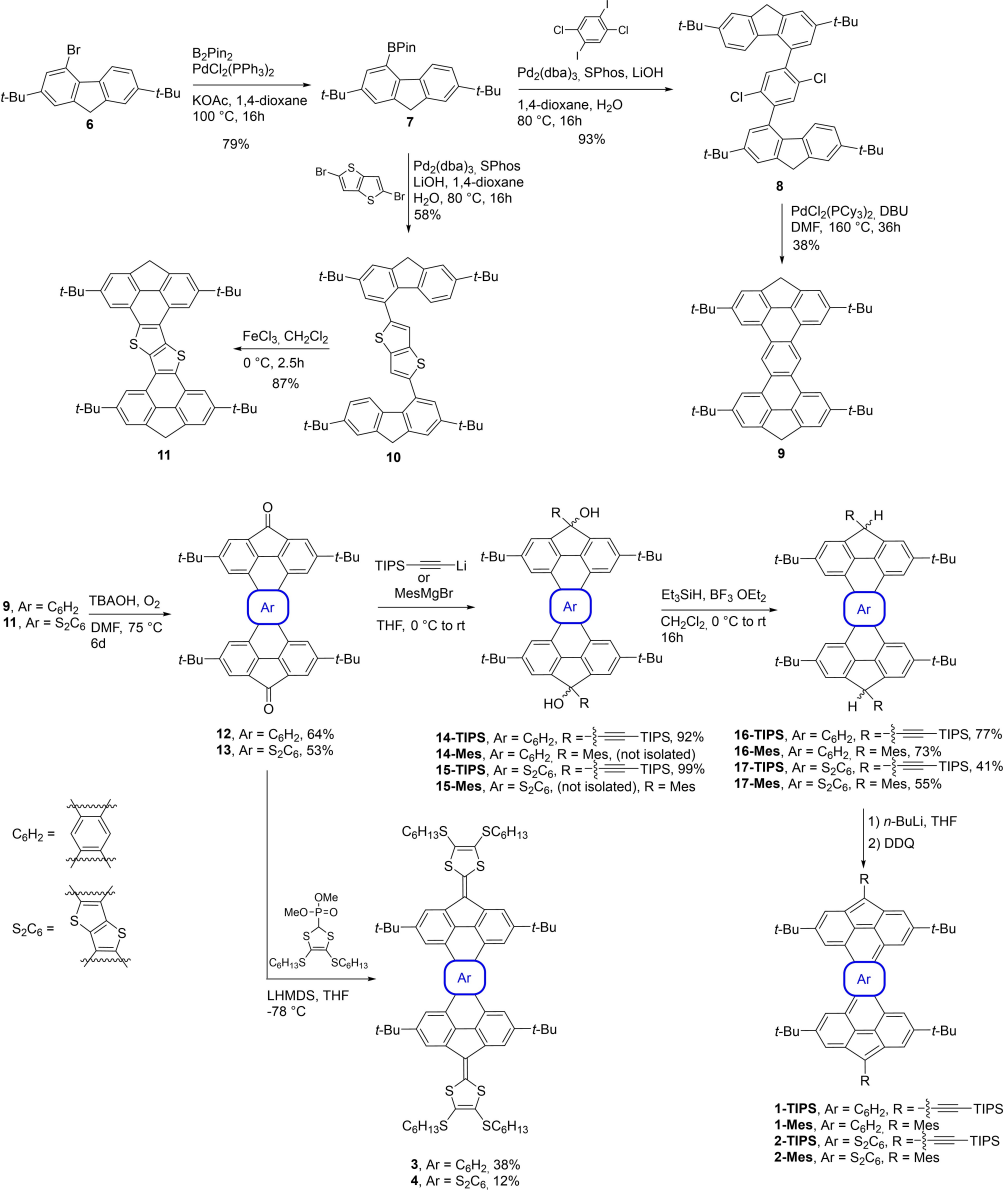
Synthesis of compounds **1‐Mes**, **1‐TIPS**, **2‐Mes**, **2‐TIPS**, **3** and **4**.

The synthesis route for compounds **1** and **2** is described in Scheme 1. Compound **6**, obtained in 2 steps from fluorene as previously reported,^[43]^ was borylated using Miyaura conditions to give compound **7** in 79 % yield. Then, a Suzuki cross‐coupling reaction with either 1,4‐dichloro‐2,6‐diiodobenzene^[44]^ or 2,5‐dibromothieno[3,2‐*b*]thiophene,^[45]^ gives compounds **8** and **10**, respectively. In order to form the fully cyclized compound **9**, compound **8** underwent a Pd‐catalyzed intramolecular cyclization reaction in a moderate yield, the main product formed during this step (53 % yield) being the monocyclized and dechlorinated compound (not shown). For the formation of thienothiophene annulated analog **11**, a Scholl reaction was performed on **10**, leading to the desired, but hardly soluble 2,5‐bis‐fluorenethieno[3,2‐*b*]thiophene **11** in 87 % yield.

After the intramolecular cyclization step, compounds **9** and **11** underwent the same synthetic pathway to afford their diradical counterparts. Oxidation under basic conditions was performed by continuously bubbling air in the solution for six days to afford the diones **12** and **13** in 64 % and 53 % yield, respectively. Then, a nucleophilic addition of lithium(triisopropylsilyl)acetylide on the ketones gives the diol intermediates **14** and **15**. Employing the well‐known reductive aromatization by the addition of a solution of SnCl_2_ in diluted HCl mostly leads to decomposition. Thus, a dehydroxylation reaction using triethylsilane and boron trifluoride etherate was performed to give molecules **16‐TIPS** and **17‐TIPS** in 77 % and 41 %, respectively. A sequence of deprotonation with *n*‐BuLi followed by oxidation with DDQ allows the formation of compounds **1‐TIPS** and **2‐TIPS**. Unfortunately, these compounds could not be isolated, presumably due to their high reactivity (high diradical character). Therefore, all subsequent characterization on compounds **1‐TIPS** and **2‐TIPS** was accomplished after their in situ generation using the deprotonation/oxidation sequence. In order to generate more stable compounds, **1‐Mes** and **2‐Mes** analogs have been synthesized by nucleophilic addition of 2‐mesitylmagnesium bromide on compounds **16** and **17**, followed by the same dihydroxylation reaction, giving respectively the protonated mesityl derivatives **16‐Mes** and **17‐Mes** in 73 % and 55 % yield.

The two extended tetrathiafulvalene (TTF) derivatives **3** and **4** were prepared in yields of 38 % and 12 %, respectively, by treating the corresponding diones **12** and **13** with the anionic form of the phosphonate ester (Scheme 1). A similar Horner–Wadsworth–Emmons olefination protocol was previously employed to prepare indenofluorene‐extended TTFs.^[46]^ The low yield for the formation of **4** could be explained by the low solubility of compound **13**, and even when an excess of the deprotonated phosphonate was used, compound **13** was recovered as the major product.

**Figure 3:**
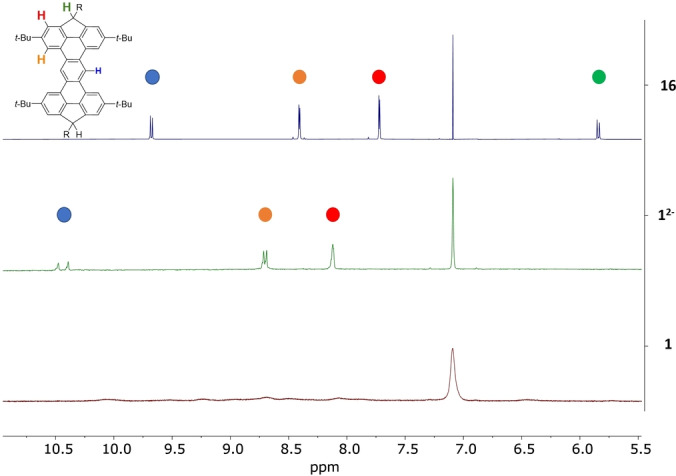
Aromatic region of ^1^H NMR spectra of compound **16‐TIPS** (top, blue), dianion **1‐TIPS^2−^** (middle, green) and **1‐TIPS** (bottom, red) at 298 K (500 MHz, benzene‐*d*_6_).

As expected, compounds **1** and **2** are NMR‐silent, which was first ascribed to a thermally accessible excited triplet state. Interestingly, decreasing the temperature to −60 °C was not sufficient to observe NMR signals. This result suggests that the diradical is actually a triplet ground state with a significant singlet‐triplet energy gap (Δ*E*_ST_). This result is consistent with a high diradical character (*y* value near 1).[^24^, ^47^, ^48^] As illustrated in Figure 3, the ^1^H NMR spectrum of compound **16‐TIPS** recorded in benzene‐*d*_6_ shows two sets of peaks that could be assigned to the *syn*/*anti* isomers. Four bundles of singlets could be observed, one at 5.84 ppm corresponding to the C−H of the sp^3^ carbon bridge, and three others in the aromatic region between 7.72 and 9.68 ppm, with the most downfield shifted one corresponding to the proton of the central phenyl unit. Upon addition of 2.0 eq. of *n*‐BuLi, all protons of the aromatic core shifted, and the signal attributed to the sp^3^ carbon disappeared, supporting the formation of the dianionic species. Oxidation of the resulting negatively charged compound with 2.0 eq of iodine caused the disappearance of the sharp peaks. No well‐resolved peak has been observed, even when the temperature was lowered to 213 K in THF‐*d*_8_. The resulting solution was analyzed by HRMS, where only the mass of the resulting diradical compound **1‐TIPS** was detected (see Supporting Information section). Similar NMR spectroscopic behavior was observed for compounds **1‐Mes**, **2‐TIPS** and **2‐Mes**.

To further evidence the formation of biradical compounds after the oxidation of the dianions, a radical quenching experiment was conducted by adding a hydrogen donor, tri‐*n*‐butyltin hydride (Bu_3_SnH), to a freshly prepared solution of **1‐Mes** in dry THF at room temperature. As shown in the ^1^H NMR spectrum (Figure S47), **16‐Mes** can be recovered with only slight formation of side products, suggesting that **1‐Mes** does not undergo significant decomposition and/or rearrangement in solution at room temperature.

**Figure 4:**
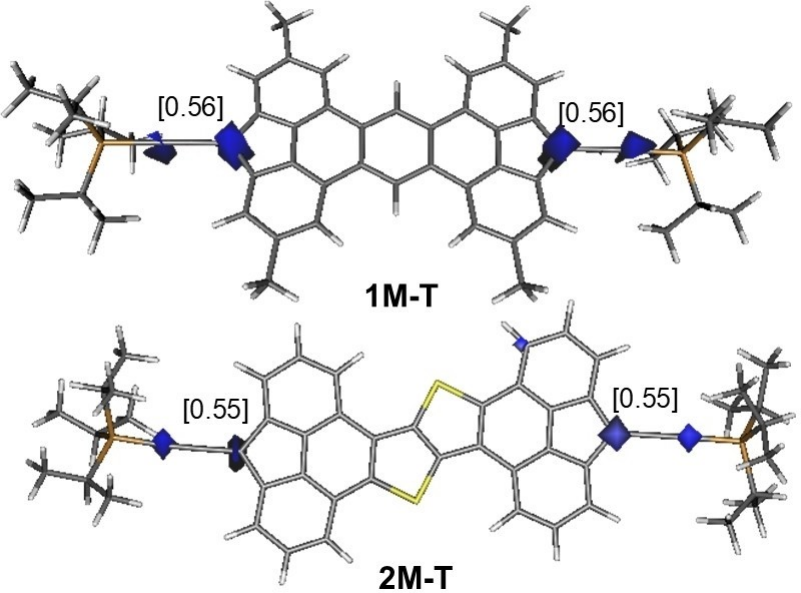
Computed (unrestricted (u) B3LYP−D3/def2‐SVP level) spin‐density of triplet species **1 M‐T and 2 M‐T** (isosurface value of 0.06 au). Values in brackets refer to the computed spin‐density at the key carbon atoms.

DFT calculations were carried out at the dispersion corrected B3LYP−D3/def2‐SVP level to gain more insight into the electronic structure of the title species. To this end, we first focused on the model compounds **1 M’** and **2 M’**, where the bulky *t*‐butyl groups in **1‐Mes** and **2‐Mes** were replaced by methyl groups. Our calculations indicate that in both cases the corresponding triplet species (**1 M’‐T** and **2 M’‐T**) are markedly more stable than their respective singlet counterparts (Δ*E*_ST_=19.6 and 19.8 kcal/mol, respectively), which is fully consistent with the experimental findings (see above). A similar scenario was found for **1 M** and **2 M**, model species of the TIPS‐alkynyl derivatives **1‐TIPS** and **2‐TIPS**, respectively, where the corresponding triplet species **1 M‐T** and **2 M‐T** are 18.2 and 18.4 kcal/mol more stable than their closed‐shell singlet counterparts. In all cases, the π‐extended 4,5‐fused bisfluorene core is highly planar and, according to the computed spin‐densities, the unpaired electrons are mainly located at the carbon atoms of the five‐membered rings directly attached to the TIPS‐alkynyl or mesityl substituents (Figure 4) although the two unpaired electrons are virtually connected through delocalization. Therefore, our calculations indicate that these molecules are better described as biradicals (independent radicals) with *y* ≥ 0.98.

**Figure 5:**
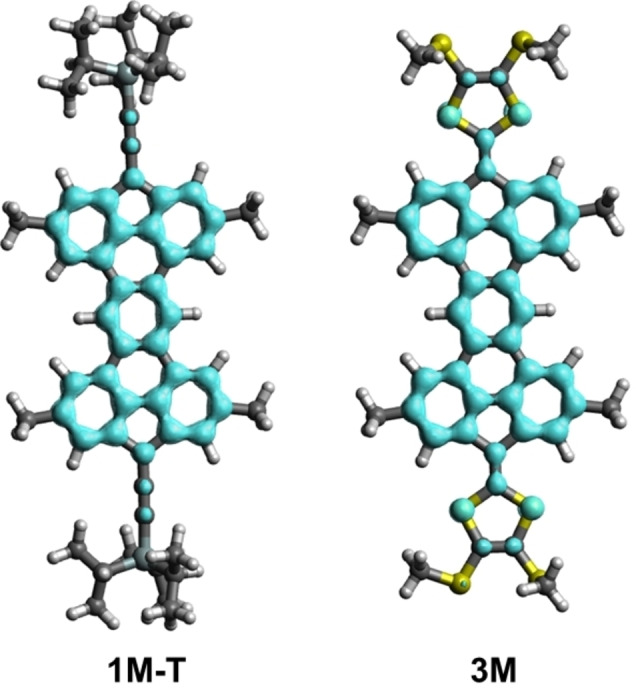
EDDB(r) contour maps computed for compounds **1  M‐T** and **3 M** isosurface value of 0.03 a.u.).

As hypothesized above, the enhanced (thermodynamic) stability of the triplet species over their corresponding closed‐shell singlet counterparts, mainly derives from the occurrence of the Clar sextets formed upon localization of the unpaired electrons at the five‐membered rings. This is supported by the comparison of the computed isotropic Nuclear Independent Chemical Shift (NICS)^[49]^ values as well as their respective, more reliable, out‐of‐plane component computed 1 Å above the ring center (NICS(1)_zz_),^[50]^ of the different rings in **1 M** and **2 M** and their triplet species. From the data in Table 1, it becomes evident that a remarkable enhancement of the aromaticity within the six‐membered rings A and C occurs in the triplet species. Thus, while these rings can be considered as non‐aromatic in the closed‐shell species, they are clearly aromatic in the corresponding open‐shell triplet states. The aromaticity increase in the central ring E is much less significant, as this ring becomes already aromatic in the singlet species. Same trends could be seen for mesityl compounds **1 M’** and **2 M’**. Furthermore, by applying the electron density of delocalized bonds (EDDB)^[51]^ method (Figure 5), the occurrence of the five Clar sextets in triplet form of **1 M (1 M‐T**), responsible for its enhanced stability, can be clearly visualized.

**Table 1:** Computed NICS(0) and NICS(1)_zz_ (within parentheses) values, in ppm. All data have been computed at the GIAO−B3LYP/def2‐SVP//B3LYP−D3/def2‐SVP level.

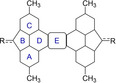					
	ring A	ring B	ring C	ring D	ring E
**1 M**	−0.6 (−4.6)	+10.6 (+23.8)	−0.6 (−4.6)	−2.3 (−9.2)	−8.5 (−25.7)
**1 M−T**	−4.4 (−14.0)	+7.0 (+14.4)	−4.4 (−14.0)	−2.1 (−9.0)	−8.8 (−26.4)
**2 M**	−2.0 (−8.2)	+9.2 (+20.3)	−2.5 (−9.3)	−4.6 (−13.0)	−8.7 (−13.4)
**2 M−T**	−5.2 (−16.2)	+6.5 (+13.2)	−5.5 (−17.5)	−4.8 (−13.8)	−9.2 (−14.7)
**1 M’**	+0.4 (−2.5)	+13.0 (+26.6)	+0.4 (−2.5)	−2.5 (−9.9)	−8.5 (−26.1)
**1 M’−T**	−3.6 (−13.2)	+8.4 (+14.8)	−3.6 (−13.2)	−2.1 (−9.4)	−8.7 (−26.6)
**2 M’**	−1.4 (−7.7)	+11.6 (+23.2)	−1.1 (−5.9)	−4.8 (−13.3)	−8.8 (−13.7)
**2 M’−T**	−4.4 (−14.4)	+7.9 (+13.8)	−4.7 (−16.7)	−4.8 (−13.8)	−9.2 (−14.7)
**3 M**	−7.8 (−22.0)	+3.8 (+7.5)	−7.8 (−22.0)	−1.6 (−7.5)	−8.3 (−25.3)
**3 M^•+^**	+2.0 (+2.9)	+11.2 (+27.0)	+2.0 (+2.9)	+0.6 (−4.2)	−4.2 (−14.6)
**3 M^2+^**	+3.5 (+6.2)	+3.8 (+37.2)	+3.5 (+6.2)	−2.1 (−8.0)	−7.6 (−23.3)
**4 M**	−8.4 (−23.1)	+3.5 (+7.2)	−8.7 (−23.7)	−4.9 (−13.6)	−8.9 (−13.8)

**Figure 6:**
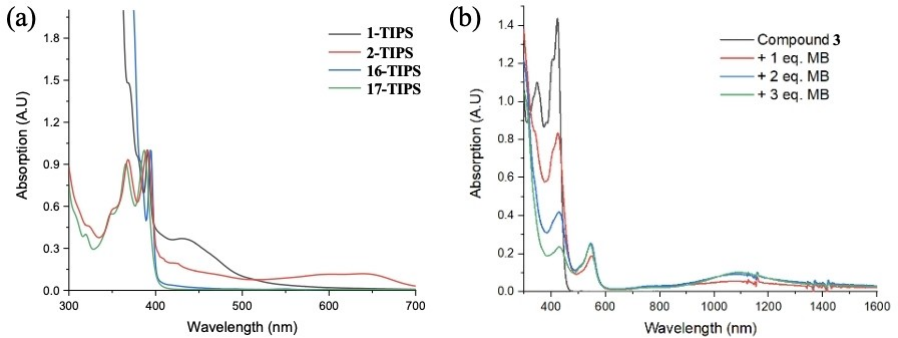
Normalized UV/Visible absorption of (a) **1‐TIPS** (black) and **2‐TIPS** (red) (both in situ generated) as well as **16‐TIPS** (blue) and **17‐TIPS** (green) in THF under a N_2_ atmosphere at 25 °C, and (b) compound **3** in the neutral (black) and oxidized (red, blue and green) forms.

The UV/Visible absorption spectra of compounds **1** and **2** and their precursors are shown in Figure 6a or Supporting Information section. The in situ generated **1‐TIPS** and **2‐TIPS** exhibit very similar features in the high‐energy region with absorption bands at 391 nm and 390 nm, respectively, which are very similar to their dehydro analogs (*λ*_max_=394 nm for **16‐TIPS** and *λ*_max_=387 nm for **17‐TIPS**). As usually observed for diradical compounds, the absorption spectra of both compounds **1** and **2** exhibit additional, less intense bands extending in the visible region, located at 430 nm (extending up to 600 nm) and 640 nm (extending up to 700 nm) for compounds **1‐TIPS** and **2‐TIPS**, respectively. According to Time Dependent‐DFT calculations (PCM‐TD−B3LYP−D3/def2‐SVP//B3LYP−D3/def2‐SVP level), for diradical **1 M‐T**, the absorption band located at ca. 600 nm (*λ*_calc_=617 nm, *f*=0.14), corresponds to the HOMO‐1→SOMO vertical transition, which confirms the π–π* nature of the transition (see Figure S48). Most open‐shell compounds often exhibit absorption up to the NIR region of the spectrum, characteristic of their extended conjugated path.[^52^, ^53^, ^54^] In this case, delocalizing the radical in the structure to form the closed‐shell mesomeric form is not favorable as five Clar sextets would need to be broken. No other resonance closed‐shell form in which fewer Clar sextets would be broken can be drawn, meaning that the unpaired electrons are highly localized on the sp^3^ carbon atom of the five‐membered ring (see above). This results in a highly aromatic, but weakly conjugated polycyclic core, leading to relatively high energy electronic transitions compared to most of the reported organic diradical molecules. This is also the hypothesis for the similar UV/Visible absorption of the diradical compounds when compared to their dihydro counterparts. Although **1‐TIPS** and **2‐TIPS** possess free radicals on their structure, the conjugation remains the same on their aromatic core, leading to similar electronic absorptions.

For TTF‐based compounds **3** and **4**, UV/Visible absorption spectroscopy (Figures 6b and S35) reveals similar absorptions between 400 and 450 nm with a band at *λ*_max_=424 and 434 nm for **3** and **4**, respectively. TD‐DFT calculations assign this band to the HOMO→LUMO vertical transition (*λ*_calc_=402 nm, *f*=0.49, for **3 M**), once again involving a π–π* transition. Compound **3**, on the other hand, shows a broad and strong absorption profile in the UV region (300–350 nm), which is not present for compound **4**. Upon oxidation of the extended TTF **3** using magic blue (MB; tris(4‐bromophenyl)ammoniumyl hexachloroantimonate), a broad absorption in the NIR region (~1100 nm) emerges (Figures 6b, S36 and S37). Importantly, this absorption concomitantly increases by adding more and more MB until two equivalents. Thus, this absorption is characteristic for both the radical cation (generated with one equivalent MB) and dication (generated with two equivalents MB) species, in line with a diradical character of the dication species. In contrast, the NIR absorptions of mono‐ and dications of indenofluorene‐extended TTFs are significantly different; indeed, for such laterally extended TTF compounds, the dication was found to exhibit quinoid rather than diradical character.^[46]^

The redox properties of both extended TTF compounds were studied by cyclic and differential pulse voltammetry in CH_2_Cl_2_ containing 0.1 M Bu_4_NPF_4_ as the supporting electrolyte. The poor solubility of compound **4** only allowed studying it at a concentration of 0.1 mM, and only weak signals were observed in the cyclic voltammogram. Thus, the reversibility of the oxidations can hardly be evaluated (see SI). Using differential pulse voltammetry, we obtain the oxidation potentials listed in Table S1. The more soluble benzene compound **3** was studied at a concentration of 0.5 mM, and the cyclic voltammogram (Figure S38) revealed two closely positioned single‐electron oxidations at +0.32 and +0.38 V vs Fc/Fc^+^, which we ascribe to converting the two dithiafulvene units to 1,3‐dithiolium rings. A further reversible oxidation is observed at +0.56 V followed by a quasi‐reversible oxidation at +0.84 V, both presumably occurring on the central π‐system, placing the charge within the core. Compound **4** shows the same trend, but with a larger gap between the first two single‐electron oxidations (+0.26 and +0.47 V vs Fc/Fc) (Figure S40), which may be explained by a better conjugation between both dithiafulvene units due to the thieno[3,2‐*b*]thiophene bridge being less aromatic than the benzene one.^[55]^

**Figure 7:**
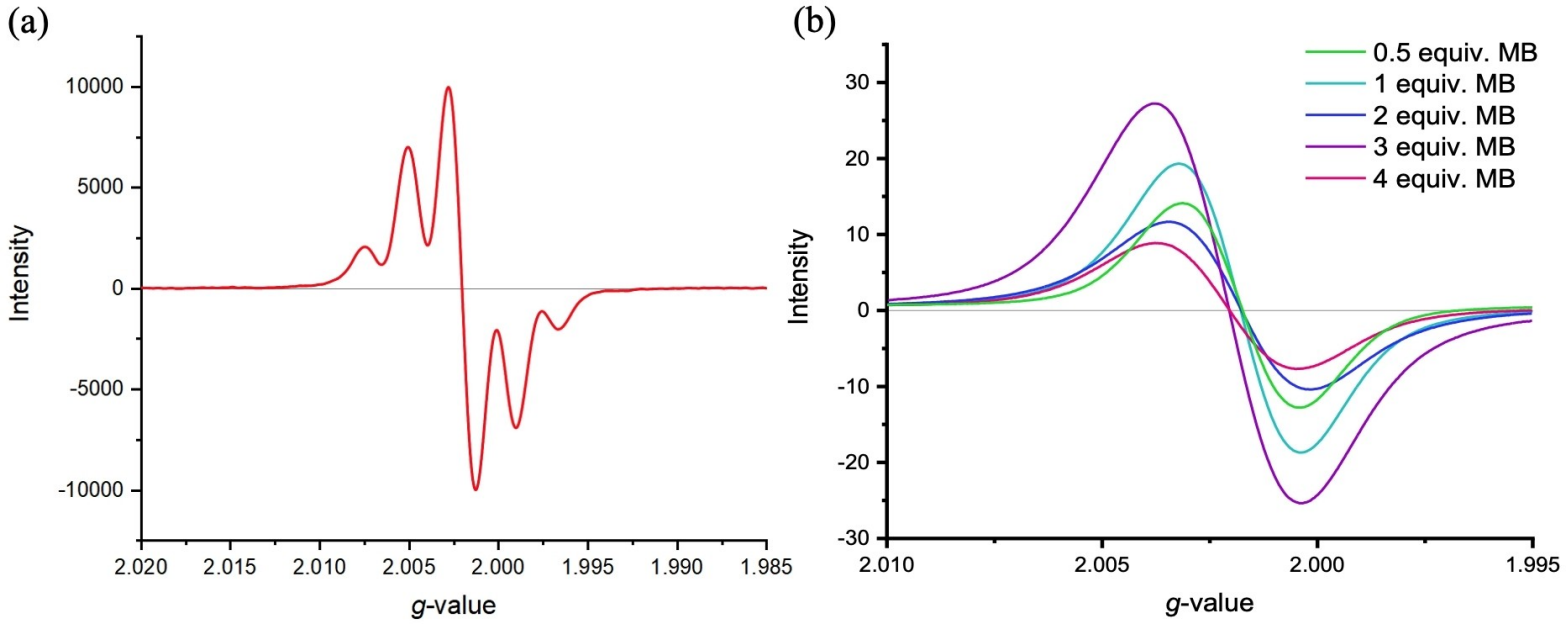
EPR spectra of (a) in situ generated **1‐Mes** in THF at room temperature and (b) **3** with variable eq. of MB in CH_2_Cl_2_ at 25 °C.

EPR spectroscopic measurements were performed on in situ generated diradical compounds **1‐TIPS**, **1‐Mes**, **2‐TIPS** and **2‐Mes** in a THF solution using *n*‐BuLi to deprotonate the methylene bridges, followed by treatment with iodine to oxidize the resulting dianion. On the one hand, both **1‐TIPS** and **2‐TIPS** compounds show broad and unresolved EPR signals (see Supporting Information section). Unfortunately, these compounds are not soluble and stable enough for precise measurements. On the other hand, **1‐Mes** (Figure 7a) and **2‐Mes** both exhibit a well‐defined quintet, which can be attributed to the coupling of the unpaired electron at each end of the molecule with the four protons of the corresponding fluorene unit (protons *ortho* to the *t*‐Bu groups). In fact, spin density calculations show that even though the spin is mostly located on the five‐membered ring, some spin density can also be found on the adjacent fluorene, giving rise to radical‐proton coupling (see Figure 4). The difference in the results for the TIPS and Mes series suggests that the bulkiness of the mesityl group surrounding the radical center is necessary to stabilize the compound.

**Scheme 2:**
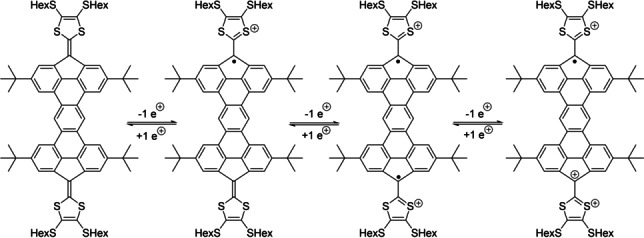
Proposed structure of cations of **3**.

EPR spectra were also recorded for compound **3** after treatment with MB as the oxidizing agent in CH_2_Cl_2_, and the spectra are shown in Figure 7. As expected, compound **3** becomes EPR‐active upon treatment with MB (converting **3** into the radical cation). The EPR signal is also present for the dication formed upon adding two equiv. of MB, albeit the signal intensity has decreased to some extent. The g‐value suggests that the dicationic species has a similar radical character as that of the monocation, suggesting a diradical structure of the dication (see Scheme 2). In consequence, the radical structure of the monocation seems similar to that of the dication, in good agreement with the two radicals located on separate fluorenyl moieties to avoid breaking the five Clar‐sextets of the π‐conjugated core. When treated with 3 equiv. of MB, a shift in the g‐value is observed, suggesting a different radical structure of the trication versus the mono‐ and dications. In this case, we suggest the π‐conjugated core is oxidized, thus making the observed radical character change as the radical is no longer located solely on the fluorene moieties.

DFT calculations were also used to gain more insight into the oxidation process involving compound **3** depicted in Scheme 2. Our calculations on the model compound **3 M**, where the *t*‐butyl and hexyl groups were replaced by methyl groups, confirm that the closed‐shell singlet is much more stable than the corresponding triplet species (Δ*E*_ST_=‐40.3 kcal/mol), which sharply contrasts with the analogous species **1‐TIPS** and highlights the crucial role of the substitution at the sp^3^ carbon bridge on the electronic structure of the system. This can be mainly ascribed to the occurrence of the Clar sextets in the singlet species as confirmed by the NICS (see Table 1) and EDDB (Figure 5) calculations. Similar results were found for the analogous species **4 M** (Δ*E*_ST_=‐48.9 kcal/mol).

**Figure 8:**
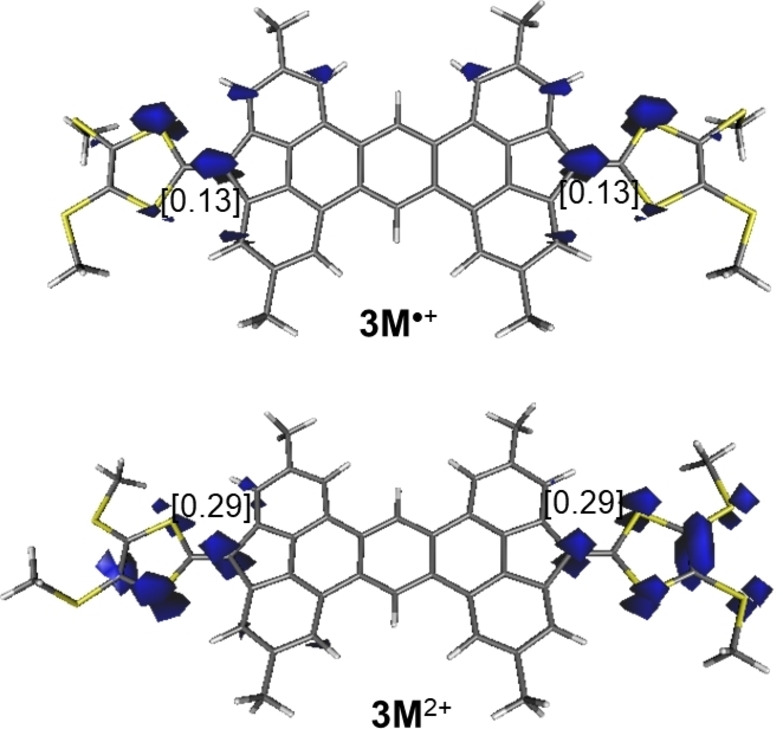
Computed (unrestricted (u) B3LYP−D3/def2‐SVP level) spin‐density of triplet species **3 M** ^•+^ and **3 M^2+^** (isosurface value of 0.02 au). Values in brackets refer to the computed spin‐density at the key carbon atoms.

The 1e‐oxidation of **3 M** leads to the corresponding radical cation **3 M^•+^** where the unpaired electron, according to the computed spin‐density (Figure 8), is equally distributed at both fluorene C(=TTF) carbon atoms. Subsequent 1e‐oxidation leads to dication **3 M^2+^**. Not surprisingly, the open‐shell (triplet) state of this species lies 10.4 kcal/mol below the corresponding closed‐shell singlet state, which confirms that the radical structure of the monocation is rather similar to that of the dication, as also confirmed by the computed NICS values (see Table 1).

## Conclusion

In summary, fusing fluorene units in a longitudinal fashion with a conjugated bridge allows for the preparation of diradical species characterized by a maximum number of Clar sextets. These diradicals exhibit a high diradical character and possess a triplet ground state with an unusual value of Δ*E*_ST_ ca. 19 kcal/mol, which is one of the highest reported to date for an all‐carbon molecule. Switching the protective groups from TIPS‐alkynyl to a bulkier one (i.e. mesityl), which does not allow delocalization, enhances the spin density on the aromatic core, leading to hyperfine coupling in the EPR spectra. By using the core as a spacer for an extended TTF, this diradical species could be obtained reversibly by oxidation/reduction. The diradical character of the various species was supported both experimentally and computationally. Efforts are underway to prepare even more stable versions of these diradicals.

## Supporting Information

Experimental procedures, NMR spectra, cyclic voltammetry, absorption spectroscopy, EPR spectroscopy and DFT calculation details.

## Conflict of Interests

The authors declare no conflict of interest.

1

## Supporting information

As a service to our authors and readers, this journal provides supporting information supplied by the authors. Such materials are peer reviewed and may be re‐organized for online delivery, but are not copy‐edited or typeset. Technical support issues arising from supporting information (other than missing files) should be addressed to the authors.

Supporting Information

## Data Availability

The data that support the findings of this study are available in the supplementary material of this article.
