# Supplementary material for: π‐Extended 4,5‐Fused Bis‐Fluorene: Highly Open‐Shell Compounds and their Cationic Tetrathiafulvalene Derivatives
Source: Angew Chem Int Ed Engl. 2024 Oct 21;63(49):e202410458. doi: 10.1002/anie.202410458 (PMC11586689; doi:10.1002/anie.202410458)
Supplement: Supplementary file 1 — Supporting Information [file ANIE-63-e202410458-s001.pdf]

Angewandte Chemie
Eine Zeitschrift der Gesellschaft Deutscher Chemiker

Supporting Information

π-Extended 4,5-Fused Bis-Fluorene: Highly Open-Shell Compounds and their Cationic Tetrathiafulvalene Derivatives

*F. Lirette, V. Bliksted Roug Pedersen, F. Gagnon, M. Brøndsted Nielsen, I. Fernández, J.-F. Morin**

SUPPORTING INFORMATION

π-Extended *4,5*-Fused Bisfluorene: Highly Open-Shell Compounds and their Cationic Tetrathiafulvalene Derivatives

Frédéric Lirette,[†] Viktor Bliksted Roug Pedersen,[§] Félix Gagnon,[†] Mogens Brøndsted Nielsen,[§] Israel Fernández,[‡] and Jean-François Morin[†,*]

[†] Département de chimie and Centre de Recherche sur les Matériaux Avancés (CERMA), 1045 Ave de la Médecine, Université Laval, Québec, Canada G1V 0A6, jean-francois.morin@chm.ulaval.ca

[§] Department of Chemistry, University of Copenhagen, Universitetsparken 5, DK-2100 Copenhagen Ø (Denmark), E-mail: mbn@chem.ku.dk

[‡] Departamento de Química Orgánica and Centro de Innovación en Química Avanzada (ORFEO-CINQA), Facultad de Ciencias Químicas, Universidad Complutense de Madrid, 28040-Madrid, Spain

Corresponding author: jean-francois.morin@chm.ulaval.ca

Table of Content

Apparatus 3
Chemicals 5
Experimental Section 5
Experimental Characterization 21
UV-visible spectroscopy 54
Chemical oxidation 55
Electrochemistry 56
EPR spectroscopy 59
Computational Details 61
References 91

Apparatus

General experimental procedures:

A Varian Inova AS400 spectrometer (Varian, Palo Alto, USA) at 400 MHz and an Agilent DD2 500 MHz were used to perform all NMR analyses. All peaks are reported on a ppm scale (δ), identified as m (multiplet), s (singlet), d (doublet), t (triplet), dd (doublet of doublets). They are reported relatively to the residual solvent peak.The coupling constant (*J*) values are reported in hertz (Hz). HRMS (High-resolution mass spectra) analyses were achieved on an Agilent 6210 TOF-LCMS instrument utilizing an APPI ion source (Agilent Technologies, Toronto, Canada).

UV-visible absorption spectroscopy:

Absorption properties of the compounds were measured in a 1 cm path length quartz cells using a Cary 7000 spectrophotometer (varian diode-array apparatus) in THF (HPLC grade). Spectra are reported with a blank THF sample absorption spectrum subtracted from them.

Electron paramagnetic resonance spectroscopy:

Solution X-band EPR spectra were recorded between -75 °C and 25 °C. Data were acquired on a Bruker Magnettech ESR 5000 spectrometer equipped with a nitrogen variable temperature unit. Microwave power was in the range 2 – 40 mW.

A solution of compounds **16** and **17** in THF was treated with 2 eq. *n*-BuLi and DDQ and an EPR spectrum was recorded.
A solution of compounds **16-Mes** and **17-Mes** in THF was treated with 2 eq. *n*-BuLi and I_2 and an EPR spectrum was recorded.

General experimental procedures for compounds 3 and 4

Anhydrous solvents were obtained from a solvent drying tower (IT model PS-MD-05). Solvents were degassed with nitrogen. Chromatographic purification was performed using silica gel (flash: 40 – 63 µm).
NMR spectra were recorded on a Bruker instrument equipped with a cryoprobe at 500 MHz and 126 MHz for ^{1}H- and ^{13}C-NMR, respectively. Deuterated CH_2Cl_2 (CD_2Cl_2, ^{1}H = 5.32 ppm, ^{13}C =

54.00 ppm) and CS_2 with a D_2O lock tube (^1H = 4.79 (D_2O) ppm, ^{13}C = 192.58 ppm (CS_2)) were used as solvents and internal references.

Chemical shift values are referenced to the ppm scale and coupling constants are expressed in Hertz (Hz). HRMS analysis was performed on a Bruker SolariX XR MALDI-FT-ICR instrument with dithranol as matrix.

UV-Vis and UV-Vis-NIR absorption spectroscopy

UV-Vis absorption spectra were recorded on a Varian Cary 50 UV-Vis spectrophotometer scanning between 800 and 200 nm. All spectra were recorded with baseline correction in $CHCl_3$ (HPLC grade) at 25 °C in a quartz cuvette with a 10 mm path length. UV-Vis-NIR absorption spectra were recorded on a Shimadzu UV-3600 CH_2Cl_2 (HPLC grade) at 25 °C in a quartz cuvette with a 10-mm path length with baseline correction and spectra are reported with a blank CH_2Cl_2 sample absorption spectrum subtracted from them. The UV-Vis-NIR spectra were recorded from dilutions of the prepared solutions **3** and MB for EPR measurements and have been recorded for qualitative features.

Electrochemistry

Cyclic voltammograms (CV) and differential pulse voltammograms (DPV) were obtained using an Autolab PGSTAT12 instrument and Nova 1.11 software with a scan rate of 0.1 V/s for the CVs and for the DPV. Ag/AgCl was used as the reference electrode, a Pt wire was used as the counter electrode, and a platinum electrode (3 mm) was used as the working electrode. The reference electrode was separated from the solution containing the substrate by a ceramic frit. Measured potentials were referenced to the ferrocene/ferrocenium (Fc/Fc^+) redox couple, measured before and after the experiment for a 1 mM solution of Fc. A 0.1 M concentration of NBu_4PF_6 was used as supporting electrolyte. All solutions were purged with argon prior to measurements. Compound **3** was measured at 0.5 mM in CH_2Cl_2. Compound **4** was measured as a saturated solution; its concentration was estimated by measuring the UV-Vis absorption spectrum - a concentration of 0.1 mM obtained from the molar absorptivity (ignoring any small difference between solvents; no change was observed in the absorption profile from the presence of the electrolyte).

Electron paramagnetic resonance spectroscopy

Solution X-band EPR spectra were recorded at room temperature. Data were acquired on a Bruker Elexsys E500spectrometer equipped with a Bruker SUPER-X CW-EPR bridge, a Bruker ER 4116 DM dual mode cavity, and an EIP 538B frequency counter. Modulation amplitudes were around 0.5 G and microwave power was in the range 2 – 20 mW.

A solution of **3** in dichloromethane was treated with an amount of magic blue (MB) in dichloromethane and an EPR spectrum was recorded. This was performed separately for the range of equivalents of MB: 0.5, 1.0, 2.0, 3.0 and 4.0 equivalents.

Chemicals

All chemical reagents were used as received and were ordered from Sigma–Aldrich Co., Canada and Oakwood Products, Inc. Phosphonate **A** (dimethyl (4,5-bis(hexylthio)-1,3-dithiol-2-yl)phosphonate) was synthesized according to literature procedure.[56]

Experimental Section

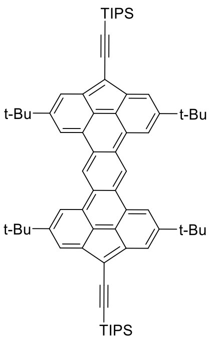

Compound 1: Compound **16** (50 mg, 0.05 mmol) was dissolved in dry THF (5 mL). The solution was degassed with nitrogen for 10 min and cooled in an ice bath. 2 eq. of *n*-BuLi was then added dropwise. The solution was stirred for 10 min at 0 °C before DDQ (23 mg, 0.1 mmol) was added quickly to the solution. The solution was stirred for 10 min before it was use for different analyses.

Other oxidizing agent such as *p*-chloranil, silver triflate (for UV-visible spectroscopy) and iodine (for NMR spectroscopy) have also been used, leading to same result. No NMR peak could be detected. Further purification leads to degradation. HRMS (APPI+): $C_{70}H_{89}Si_2$ $[M+H]^+$ 985.6503; found 985.6497.

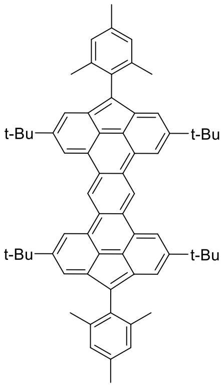

Compound 1-Mes: Compound **16-Mes** (50 mg, 0.05 mmol) was dissolved in dry THF (5 mL). The solution was degassed with nitrogen for 10 min and cooled in an ice bath. 2 eq. of *n*-BuLi was then added dropwise. The solution was stirred for 10 min at 0 °C before I_2 was added quickly to the solution. The solution was stirred for 10 min before it was use for different analyses. No NMR peak could be detected. Further purification leads to degradation. HRMS (APPI+): $C_{66}H_{69}$ $[M+H]^+$ 861.5399; found 861.5394.

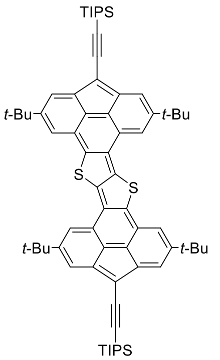

Compound 2: Compound **17** (70 mg, 0.066 mmol) was dissolved in dry THF (5 mL). The solution was degassed with nitrogen for 10 min and cooled in an ice bath. 2 eq. of *n*-BuLi was then added dropwise. The solution was stirred for 10 min at 0 °C before DDQ (30 mg, 0.13 mmol) was added quickly to the solution. The solution was stirred for 10 min before it was use for different analyses.

Other oxidizing agent such as *p*-chloranil, silver triflate (for UV-visible spectroscopy) and iodine (for NMR spectroscopy) have also been used, leading to same result. No NMR peak could be detected. Further purification leads to degradation. HRMS (APPI+): $C_{70}H_{87}S_2Si_2$ $[M+H]^+$ 1047.5788; found 1047.5782.

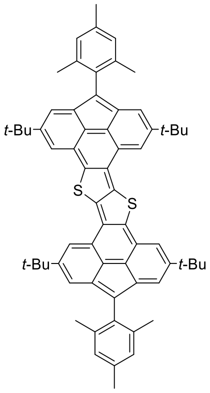

Compound 2-Mes: Compound **17-Mes** (50 mg, 0.05 mmol) was dissolved in dry THF (5 mL). The solution was degassed with nitrogen for 10 min and cooled in an ice bath. 2 eq. of *n*-BuLi was then added dropwise. The solution was stirred for 10 min at 0 °C before I_2 was added quickly to the solution. The solution was stirred for 10 min before it was use for different analyses. No NMR peak could be detected. Further purification leads to degradation. HRMS (APPI+): $C_{66}H_{67}S_2$ $[M+H]^+$ 923.4684; found 923.4679.

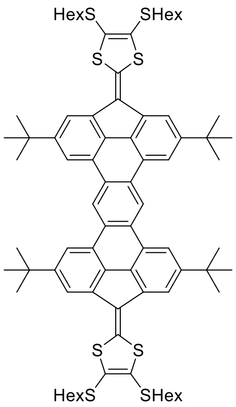

Compound 3: To a flame-dried round-bottomed Schlenk flask equipped with a magnetic stir bar was added dione compound **12** (39.9 mg, 61 μmol). The flask was evacuated and backfilled with

nitrogen four times, after which anhydrous THF (20 mL) was added, and the suspension was degassed with nitrogen under sonication for 15 minutes. The flask was then cooled in a dry ice/acetone bath for 1 hour. In a separate flame-dried round-bottomed Schlenk flask equipped with a magnetic stir bar was added dimethyl (4,5-bis(hexylthio)-1,3-dithiol-2-yl)phosphonate (181.5 mg, 408 μmol, 6.7 equiv.), and the flask was evacuated and backfilled with nitrogen four times, after which anhydrous THF (10 mL) was added. The flask was then cooled in a dry ice/acetone bath for 25 minutes before a solution of LiHMDS in THF (1 M, 0.36 mL 360 μmol, 5.9 equiv.) was added dropwise. The clear orange solution slowly turned darker upon addition of the LiHMDS, and the solution was stirred for 50 minutes before the resulting dark greenish brown solution was cannulated dropwise into the flask containing compound **12**. The mixture was allowed to stir in the dry ice/acetone bath overnight allowing it to slowly reach room temperature. The following day the orange reaction mixture was poured into aqueous sat. NH_4Cl (25 mL) and extracted with dichloromethane (50 mL, then 2 x 25 mL). The organic phases were combined and washed with water (50 mL) before it was dried over Na_2SO_4, then filtered and the volatiles removed under reduced pressure. The resulting yellow oil was purified by flash column chromatography (silica gel, 40-63 μm) with carbon disulfide as eluent. The compound was collected as a yellow band, and recrystallized from dichloromethane by a layer of pentane, resulting in a fluffy yellow solid, which was washed with pentane and dried under reduced pressure yielding the **3** as a yellow solid (29.5 mg, 23 μmol) in 37% yield.^{1}H NMR (500 MHz, CD_2Cl_2) δ 9.51 (s, 2H), 8.47 (s, 4H), 7.83 (s, 4H), 2.99 (t, 8H, *J* = 7.4), 1.80-1.74 (m, 8H), 1.74 (s, 36H), 1.54-1.48 (m, 8H), 1.37-1.34 (m, 16H), 0.91 (t, *J* = 7.0 Hz, 12H). ^{13}C NMR (126 MHz, CD_2Cl_2) δ 151.32, 139.32, 136.21, 131.94, 129.70, 128.84, 125.60, 121.77, 119.36, 118.97, 115.41, 36.97, 36.43, 32.69, 31.84, 30.44, 28.77, 23.02, 14.23. HRMS ($MALDI^+$ FT-ICR, dithranol): *m/z* = 1290.3997 [$M^{\bullet+}$], calcd. for [$C_{78}H_{98}S_8^{\bullet+}$] = 1290.54287

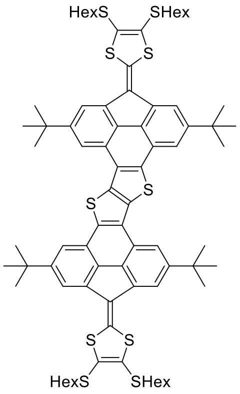

Compound 4: To a flame-dried round-bottomed Schlenk flask equipped with a magnetic stir bar was added dione compound **13** (54.2 mg, 76 μmol). The flask was evacuated and backfilled with nitrogen four times, after which anhydrous THF (50 mL) was added, and the suspension was degassed with nitrogen under sonication for 15 minutes. The flask was then cooled in a dry ice/acetone bath for 1 hour. In a separate flame-dried round-bottomed Schlenk flask equipped with a magnetic stir bar was added phosphonate dimethyl (4,5-bis(hexylthio)-1,3-dithiol-2-yl)phosphonate (246.3 mg, 521 μmol, 6.9 equiv.), and the flask was evacuated and backfilled with nitrogen four times, after which anhydrous THF (20 mL) was added. The flask was then cooled in a dry ice/acetone bath for 30 minutes before a solution of LiHMDS in THF (1 M, 0.45 mL 450 μmol, 6.0 equiv.) was added dropwise. The clear orange solution slowly turned darker upon addition of the LiHMDS, and the solution was stirred for 1 hour before the resulting dark greenish brown solution was cannulated dropwise into the flask containing dione compound **13**. The mixture was allowed to stir in the dry ice/acetone bath overnight allowing it to slowly reach room temperature. The following day the orange reaction mixture was poured into aqueous sat. NH_4Cl (50 mL) and extracted with carbon disulfide (2 x 50 mL). The organic phases were combined and dried over Na_2SO_4, filtered and the volatiles removed under reduced pressure to yield an orange solid. The solid was sonicated in MeOH, centrifuged and the solvent decanted off; this process was repeated for a total of three times with MeOH and subsequently three times with pentane. The resulting yellow solid was dissolved in carbon disulfide and passed through a silica gel plug and recrystallized from a bilayer solution of dichloromethane and heptane to give compound **4** as a yellow solid (11.9 mg, 8.8 μmol) in 12% yield. ^{1}H NMR (500 MHz, CS_2) δ 8.56 (s, 2H), 8.26 (s, 2H), 8.09 (s, 2H), 8.02 (s, 2H), 3.29 (t, *J* = 7.3 Hz, 4H), 3.28 (t, *J* = 7.3 Hz, 4H), 2.10-2.04 (m, 8H) 2.05 (s, 18H), 1.98 (s, 18H), 1.86-1.80 (m, 8 H), 1.70-1.67 (m, 16H), 1.26 (m, 12H). ^{13}C NMR (126 MHz, CS_2) δ 151.08, 150.91, 139.42, 138.99, 136.43, 136.19, 134.10, 131.40, 130.98, 129.15,

128.16, 124.33, 122.82, 122.47, 118.53, 118.38, 116.35, 114.94, 37.17, 37.16, 35.96, 35.94, 32.70, 32.58, 32.02, 30.45, 28.99, 23.43, 14.74. 1 sp^2 and 5 sp^3-carbon signals missing, presumably due to overlap. HRMS ($MALDI^+$ FT-ICR, dithranol): *m/*z = 1352.46979 [$M^{\bullet+}$], calcd. for [$C_{78}H_{98}S_8^{\bullet+}$] = 1352.47136.

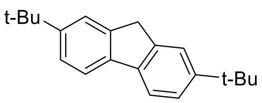

Compound 5: To a solution of fluorene (10.0 g, 60.1 mmol) in dichloromethane (85 mL) cooled in an ice bath, under a nitrogen atmosphere, was added iron trichloride (0.97 g, 6.0 mmol) followed by 2-chloro-2-methylpropane (13.8 mL, 125 mmol) over a period of 10 min. The reaction mixture was stirred at 0 °C for 3 h. The resulting mixture was poured into water (200 mL) and washed with a 1 M aqueous solution of hydrochloric acid (100 mL). The aqueous layer was further extracted with dichloromethane and the organic extracts were combined and washed with brine. The organic layer was dried ($MgSO_4$), evaporated under reduced pressure and the crude product was purified by column chromatography (silica gel, 100% hexanes). The resulting product was recrystallized in ethanol to afford the desired compound as a white solid (14.5 g, 87%).^{1}H NMR (400 MHz, chloroform-*d*) δ 7.66 (dd, *J* = 8.0, 0.6 Hz, 2H), 7.56 (dd, *J* = 1.8, 0.8 Hz, 2H), 7.39 (dd, *J* = 8.1, 1.8 Hz, 2H), 3.86 (s, 2H), 1.38 (s, 18H). ^{13}C NMR (101 MHz, chloroform-*d*) δ 149.43, 143.25, 139.11, 123.76, 121.85, 119.06, 37.08, 34.80, 31.62. HRMS (APPI+): $C_{21}H_{27}$ $[M+H]^+$ 279.2113; found 279.2107.

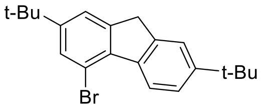

Compound 6: I_2 (0.10 g, 0.43 mmol) and compound **5** (3.00 g, 7.2 mmol) were dissolved in dichloromethane (14 mL) at 0 °C. A solution of Br_2 (0.4 mL, 7.8 mmol) in dichloromethane (6 mL) was added dropwise, and the mixture was stirred for 1 h at 0 °C. The product was extracted with diethyl ether, washed with water and NaOH solution. The organic layer was dried ($MgSO_4$), evaporated under reduced pressure and the crude product was purified by recrystallisation in

ethanol to afford the desired compound as a white solid (2.15 g, 84 %). ^{1}H NMR (400 MHz, chloroform-*d*) δ 8.47 (d, *J* = 8.3 Hz, 1H), 7.57 (d, *J* = 1.7 Hz, 1H), 7.53 (d, *J* = 1.6 Hz, 1H), 7.50 (d, *J* = 1.6 Hz, 1H), 7.46 (dd, *J* = 8.3, 1.9 Hz, 1H), 3.91 (s, 2H), 1.40 (s, 9H), 1.37 (s, 9H). ^{13}C NMR (126 MHz, chloroform-*d*) δ 150.92, 150.15, 145.87, 143.58, 138.40, 137.41, 128.70, 123.58, 122.60, 121.57, 120.93, 116.21, 37.43, 34.83, 34.78, 31.53, 31.42. HRMS (APPI+): $C_{21}H_{26}Br$ $[M+H]^+$ 357.1218; found 357.1212.

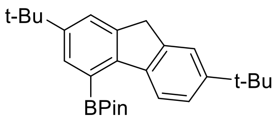

Compound 7: Compound **6** (3.60 g, 10.1 mmol), Bis(pinacolato)diboron (2.8 g, 7.5 mmol), [1,1′-bis(diphenylphosphino)ferrocene]dichloropalladium(II) (247 mg, 0.30 mmol) and potassium acetate (3.0 g, 31 mmol) were added to a screw-capped pressure vessel under nitrogen. The mixture was degassed with a flow of nitrogen for 15 min. Nitrogen-purged 1,4-dioxane (50 mL) was added. The mixture was purged three times with nitrogen and heated at 100 °C for 16 h. After cooling at room temperature, the solvent was removed under reduced pressure. The crude product was purified on a short pad of silica (dichloromethane/hexanes 1:1 v/v). The solvent was removed under reduce pressure and the resulting solid was purified by recrystallisation in ethanol to afford the desired compound as a white solid (3.21 g, 79%). ^{1}H NMR (500 MHz, chloroform-*d*) δ 8.59 (d, *J* = 8.3 Hz, 1H), 7.82 (d, *J* = 2.0 Hz, 1H), 7.63 (d, *J* = 2.1 Hz, 1H), 7.53 (d, *J* = 2.0 Hz, 1H), 7.40 (dd, *J* = 8.3, 1.9 Hz, 1H), 3.85 (s, 2H), 1.46 (s, 12H), 1.40 (s, 9H), 1.39 (s, 9H). ^{13}C NMR (101 MHz, chloroform-*d*) δ 193.83, 149.16, 147.85, 143.86, 143.83, 143.54, 140.25, 131.36, 124.51, 123.60, 123.05, 121.32, 83.76, 37.14, 34.69, 34.62, 31.58, 31.56, 25.00. HRMS (APPI+): $C_{27}H_{38}BO_2$ $[M+H]^+$ 405.2965; found 405.2964.

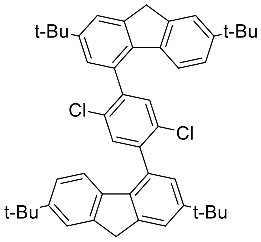

Compound 8: Compound **7** (2.0 g, 7.4 mmol), 1,4-dichloro-2,5-diiodobenzene (1.45 g, 3.6 mmol) and lithium hydroxide monohydrate (630 mg, 15 mmol) were added to a round-bottom flask. 1,4-dioxane (38 mL) and water (9.5 mL) were added. The mixture was degassed with a flow of nitrogen for 15 min. Tris(dibenzylideneacetone) dipalladium(0) (167 mg, 0.18 mmol) and SPhos (300 mg, 0.7 mmol) were added. The mixture was purged three times with nitrogen and heated at 85 °C for 16 h. After cooling at room temperature, the mixture was extracted with dichloromethane and washed with brine and the solvent was removed under reduced pressure. The crude product was washed with a AcOEt/hexanes (15/85 %) solution and filtered to afford the desired compound as a light-gray solid (2.36 g, 93%). There are two isomers of the compound. ^{1}H NMR (isomer 1) (500 MHz, chloroform-*d*) δ 7.66 (d, *J* = 1.8 Hz, 2H), 7.63 (s, 2H), 7.63 (dd, *J* = 1.9, 0.7 Hz, 2H), 7.34 (dd, *J* = 8.2, 1.9 Hz, 2H), 7.29 (d, *J* = 1.8 Hz, 2H), 7.07 (dd, *J* = 8.2, 0.6 Hz, 2H), 3.99 (d, *J* = 5.2 Hz, 4H), 1.44 (s, 18H), 1.39 (s, 18H). ^{1}H NMR (isomer 2) (400 MHz, chloroform-*d*) δ 7.66 (d, *J* = 1.8 Hz, 2H), 7.62 (s, 2H), 7.58 (dd, *J* = 2.0, 0.7 Hz, 2H), 7.31 (d, *J* = 1.8 Hz, 2H), 7.26 – 7.25 (m, 2H), 6.88 (dd, *J* = 8.2, 0.6 Hz, 2H), 3.97 (s, 4H), 1.47 (s, 18H), 1.36 (s, 18H). ^{13}C NMR (mixture) (126 MHz, chloroform-*d*) δ 149.60, 149.33, 143.96, 143.89, 143.83, 143.76, 141.01, 138.64, 136.71, 136.53, 132.41, 132.23, 132.01, 131.95, 125.78, 125.35, 123.87, 123.75, 121.94, 121.82, 121.39, 121.31, 67.10, 34.91, 34.77, 34.75, 31.68, 31.61, 31.58, 31.55.. HRMS (APPI+): $C_{48}H_{53}Cl_2$ $[M+H]^+$ 699.3524; found 699.3519.

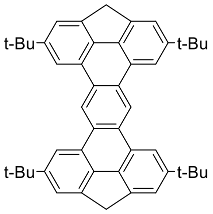

Compound 9: A screw-capped pressure vessel under nitrogen was charged with compound **8** (2.8 g, 4.0 mmol), dichlorobis(tricyclohexylphosphine)palladium(II) (590 mg, 0.8 mmol) and 1,8-

diazabicyclo[5.4.0]undec-7-ene (7.3 g, 48.0 mmol). The mixture was purged and degassed with a flow of nitrogen for 30 min. Degassed dimethylformamide (50 mL) was added. The mixture was heated at 160 °C for 36 h. The mixture was precipitated in water, filtered and washed with ethanol and a dichloromethane/hexanes (1/9 v/v) solution. The precipitate was solubilized in warm chloroform and purified on a short pad of silica (100 % chloroform) to afford the desired compound as a white solid (0.94 g, 38%). ^{1}H NMR (500 MHz, chloroform-*d*) δ 9.88 (s, 2H), 8.64 (d, *J* = 1.1 Hz, 4H), 7.90 (d, *J* = 1.1 Hz, 4H), 4.35 (s, 4H), 1.64 (s, 36H). ^{13}C NMR (126 MHz, chloroform-*d*) δ 150.83, 141.58, 135.66, 129.79, 125.69, 120.93, 118.98, 115.60, 37.72, 35.92, 32.26. HRMS (APPI+): $C_{48}H_{51}$ $[M+H]^+$ 627.3991; found 627.3985.

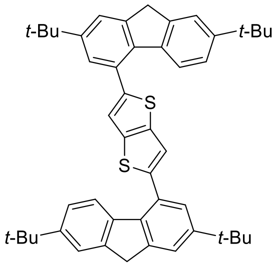

Compound 10: Compound **7** (3.6 g, 8.9 mmol), 2,5-dibromothieno[3,2-*b*]thiophene (1.3 g, 4.4 mmol) and lithium hydroxide monohydrate (760 mg, 18 mmol) were added to a round-bottom flask. 1,4-dioxane (44 mL) and water (12 mL) were added. The mixture was degassed with a flow of nitrogen for 15 min. Tris(dibenzylideneacetone) dipalladium(0) (200 mg, 0.22 mmol) and SPhos (358 mg, 0.87 mmol) were added. The mixture was purged three times with nitrogen and heated at 85 °C for 16 h. After cooling at room temperature, the mixture was extracted with DCM and washed with brine and the solvent was removed under reduced pressure. The crude product was washed with a AcOEt/hexanes (5/100, v/v) solution and filtered to afford the desired compound as an off-white solid (1.74 g, 58%). ^{1}H NMR (500 MHz, chloroform-*d*) δ 7.63 (dt, *J* = 1.8, 0.9 Hz, 2H), 7.58 (dd, *J* = 1.9, 0.8 Hz, 2H), 7.46 (d, *J* = 1.8 Hz, 2H), 7.41 (s, 2H), 7.38 (dd, *J* = 8.3, 0.6 Hz, 2H), 3.96 (s, 4H), 1.43 (s, 18H), 1.36 (s, 18H). ^{13}C NMR (126 MHz, chloroform-*d*) δ 149.67, 149.03, 144.19, 143.80, 143.76, 139.08, 138.53, 137.55, 128.70, 127.30, 123.73, 122.28, 122.17, 121.70, 119.09, 37.15, 34.76, 34.76, 31.55 (d, *J* = 4.8 Hz). HRMS (APPI+): $C_{48}H_{53}S_2$ $[M+H]^+$ 693.35887; found 693.3583.

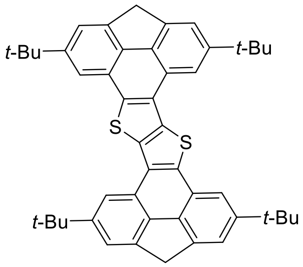

Compound 11: Compound **10** (650 mg, 0.94 mmol) and iron(III) chloride (1.52 g, 9.4 mmol) were added to a round-bottom flask under inert atmosphere in an ice bath. Dry dichloromethane (90 mL) was added to the flask and the resulting mixture was stirred for 2.5 h at 0 °C. The mixture was quenched with methanol, filtered and washed with methanol, water and a small volume of dichloromethane to afford the desired compound as an off-white solid (560 mg, 87%). Compound **11** is not soluble enough to give a clean ^{1}H NMR spectrum. ^{1}H NMR (500 MHz, chloroform-*d*) δ 8.48 (s, 2H), 8.14 (s, 2H), 7.94 (s, 2H), 7.87 (s, 2H), 4.44 (s, 4H), 1.56 (s, 36H). Compound **11** is not soluble enough to give a NMR ^{13}C spectrum. HRMS (APPI+): $C_{48}H_{59}S_2$ $[M+H]^+$ 689.3276; found 689.3270.

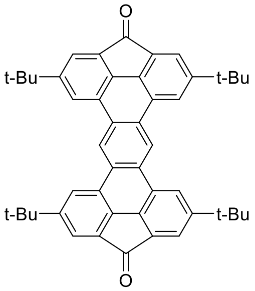

Compound 12: Compound **9** (590 mg, 0.94 mmol), tetrabutylammonium hydroxide 1M (5.65 mL, 5.65 mmol) and dimethylformamide (400 mL) were added in a round-bottom flask. The mixture was heated at 75 °C and bubbled with air for 6 days. 5 to 10 mL of dimethylformamide was added each day to compensate for the evaporation. The mixture was diluted in 400 mL of 2M HCl aq., filtered and washed with water, ethanol and dichloromethane to afford the desired compound as a yellow solid (0.394 mg, 64%). Compound **12** is not soluble enough to give NMR spectra. HRMS (APPI+): $C_{48}H_{47}O_2$ $[M+H]^+$ 655.3576; found 655.3571.

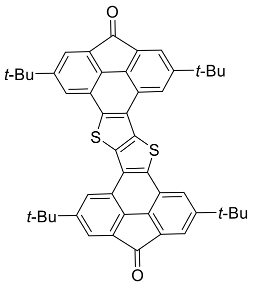

Compound 13: Compound **11** (1.19 g, 1.73 mmol), tetrabutylammonium hydroxide 1 M in methanol (10.36 mL, 10.36 mmol) and dimethylformamide (210 mL) were added in a round-bottom flask. The mixture was heated at 75 °C and bubbled with air for 6 days. 5 to 10 mL of dimethylformamide was added each day to compensate for the evaporation. The mixture was diluted in 200 mL of HCl 2M, filtered and washed with water, ethanol and dichloromethane to afford the desired compound as a yellow solid (660 mg, 53 %). Compound **13** is not soluble enough to give NMR spectra and HRMS spectrum.

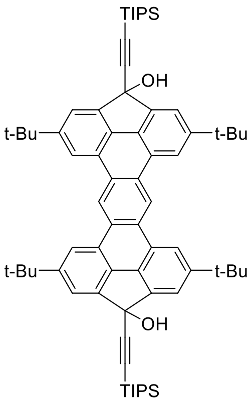

Compound 14: Dry THF (9 mL) and triisopropylsilylacetylene (0.4 mL, 1.8 mmol) were added to a round-bottom flask under inert atmosphere in an ice bath. *n*-BuLi (2.5 M, 1.8 mmol) was added dropwise and the resulting mixture was stirred for 30 min at 0 °C. Compound **12** (150 mg, 0.22 mmol) was added quickly to the solution. The mixture was stirred for 16 h at room temperature. The reaction was extracted with dichloromethane and washed with water, dried over $MgSO_4$ and the solvent was removed under reduced pressure. The crude product was purified by column chromatography (silica gel, 100% dichloromethane) to afford the desired compound as a white solid (205 mg, 92%). ^{1}H NMR (400 MHz, chloroform-*d*) δ 9.75 (s, 2H), 8.59 (d, *J* = 1.1 Hz, 4H), 8.01 (d, *J* = 1.1 Hz, 4H), 2.81 (s, 2H), 1.61 (s, 42H).^{13}C NMR (126 MHz, chloroform-*d*) δ 152.60,

146.01, 132.69, 129.63, 125.81, 120.20, 119.13, 118.19, 106.78, 85.73, 77.83, 36.11, 32.04, 18.60, 11.17. HRMS (APPI+): $C_{70}H_{91}O_2Si_2$ $[M+H]^+$ 1019.6558; found 1019.6552.

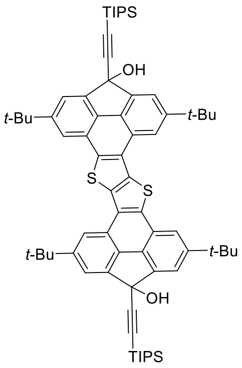

Compound 15: Dry THF (35 mL) and triisopropylsilylacetylene (1.25 mL, 5.6 mmol) were added to a round-bottom flask under inert atmosphere in an ice bath. *n*-BuLi (2.5 M, 5.6 mmol) was added dropwise and the resulting mixture was stirred for 30 min at 0 °C. Compound **13** (500 mg, 0.7 mmol) was added quickly to the solution. The mixture was stirred for 16 h at room temperature. The reaction was extracted with dichloromethane and washed with water, dried over $MgSO_4$ and the solvent was removed under reduced pressure. The crude product was purified by column chromatography (silica gel, 100% dichloromethane) to afford the desired compound as a white solid (750 mg, 99%). ^{1}H NMR (400 MHz, chloroform-*d*) δ 8.48 – 8.42 (m, 2H), 8.13 – 8.10 (m, 2H), 8.06 – 8.04 (m, 2H), 8.00 – 7.97 (m, 2H), 2.85 (s, 2H), 1.66 (s, 18H), 1.59 (s, 18H), 1.11 – 1.07 (m, 42H). ^{13}C NMR (126 MHz, chloroform-*d*) δ 153.21, 153.10, 146.37, 146.16, 138.85, 133.81, 132.48, 131.89, 128.17, 124.76, 123.21, 119.13, 118.87, 118.76, 117.53, 106.44, 94.75, 86.25, 85.96, 78.39, 36.22, 32.18, 32.05, 30.32, 18.61, 18.59, 18.46, 18.43, 11.15, 11.01. HRMS (APPI+): $C_{70}H_{89}O_2S_2Si_2$ $[M+H]^+$ 1081.5843 found 1081.5837.

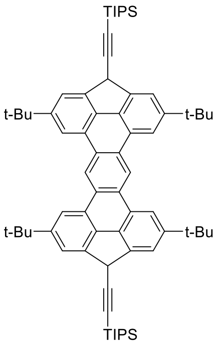

Compound 16: Compound **14** (100 mg, 0.10 mmol) was solubilized in 5 mL of dichloromethane in a round-bottom flask at 0 °C. Triethylsilane (0.12 mL, 0.70 mmol) and boron trifluoride diethyl etherate (0.09 mL, 0.7 mmol) were added and the resulting mixture was stirred at 0 °C for an hour, then 12 hours at room temperature. The solution was extracted with chloroform, washed with $NaHCO_3$ aq., dried with $MgSO_4$ and the solvent was removed under reduced pressure. The crude product was purified by column chromatography (silica gel, dichloromethane/hexanes (1:5 v/v) to afford the desired compound as a white solid (76 mg, 77 %). ^{1}H NMR (500 MHz, chloroform-*d*) δ 9.87 (s, 1H), 9.85 (s, 1H), 8.59 (d, *J* = 4.1 Hz, 4H), 7.90 (d, *J* = 3.6 Hz, 4H), 6.03 (s, 1H), 6.01 (s, 1H), 1.64 (s, 36H), 1.24 – 1.19 (m, 42H). ^{13}C NMR (126 MHz, chloroform-*d*) δ 207.11 (d, *J* = 2.4 Hz), 150.92, 150.90, 137.66, 131.50, 129.81, 129.80, 125.69, 119.23, 119.20, 118.62, 115.53, 100.11, 84.07, 84.03, 35.98, 32.23, 18.53, 11.39. Some carbon signals are duplicated due to the presence of two isomers. HRMS (APPI+): $C_{70}H_{91}Si_2$ $[M+H]^+$ 987.6659; found 987.6654.

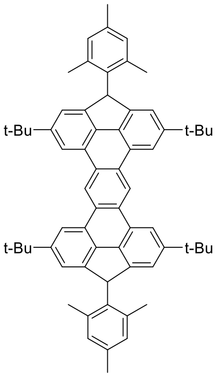

Compound 16-Mes: Dry THF (50 mL) and compound **12** (400 mg, 0.6 mmol) were added to a round-bottom flask under inert atmosphere and cooled at -78 °C. 2-mesitylmagnesium bromide 1.0 M in THF (4.78 mL) was added dropwise and the resulting mixture was stirred for 30 min at -78 °C. The mixture was stirred for 16 h at room temperature. 15 mL of DCM and 20 mL of water was

added to the flask and the organic solvent was removed under reduced pressure, leading to a suspension of white solid in water. The crude product was filtered and rinsed several times with MeOH. 350 mg of the resulting powder and 60 mL of dichloromethane were added in a round-bottom flask at 0 °C. Triethylsilane (0.47 mL, 2.57 mmol) and boron trifluoride diethyl etherate (0.36 mL, 2.8 mmol) were added and the resulting mixture was stirred at 0 °C for an hour, then 12 hours at room temperature. The reaction was quenched with $NaHCO_3$ aq., and the organic solvent was removed under reduced pressure. The crude product was filtered, rinsed with clean water and triturated with a solution of AcOEt/Hexanes (3:7 v/v) to afford the desired compound as a white solid (385 mg, 73%). ^{1}H NMR (500 MHz, chloroform-*d*) δ 9.95 (s, 1H), 9.94 (s, 1H), 8.67 (d, *J* = 1.0 Hz, 2H), 8.67 (d, *J* = 1.5 Hz, 2H), 7.58 (d, *J* = 1.2 Hz, 2H), 7.57 (d, *J* = 1.1 Hz, 2H), 7.19 – 7.07 (m, 2H), 6.77 – 6.60 (m, 2H), 6.02 (s, 2H), 2.85 (s, 6H), 2.36 (s, 6H), 1.57 (s, 36H), 1.06 (s, 6H). ^{13}C NMR (126 MHz, chloroform-*d*)) δ 151.41, 145.50, 138.45, 137.62, 136.09, 134.61, 133.66, 130.45, 129.90, 128.86, 125.75, 119.91, 119.14, 115.77, 51.61, 35.96, 32.24, 21.99, 20.94, 19.05. Some carbon signals are duplicated due to the presence of two isomers. HRMS (APPI+): $C_{66}H_{71}$ $[M+H]^+$ 863.5556; found 863.5550.

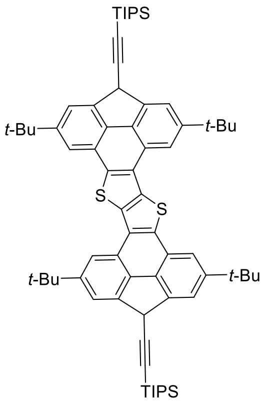

Compound 17: Compound **15** (750 mg, 0.70 mmol) was solubilized in 5 mL of dichloromethane in a round-bottom flask at 0 °C. Triethylsilane (0.93 mL, 5.8 mmol) and boron trifluoride diethyl etherate (0.74 mL, 6.0 mmol) were added and the resulting mixture was stirred at 0 °C for an hour, then 12 hours at room temperature. The solution was extracted with chloroform and washed with $NaHCO_3$ aq., dried with $MgSO_4$ and the solvent was removed under reduced pressure. The crude product was purified by column chromatography (silica gel, dichloromethane/hexanes (1:2 v/v)

and triturated in isopropanol to afford the desired compound as a white solid (338 mg, 41 %). ^{1}H NMR (400 MHz, chloroform-*d*) δ 8.48 (s, 1H), 8.47 (s, 1H), 8.17 – 8.11 (m, 2H), 8.02 (d, *J* = 1.1 Hz, 1H), 7.96 (d, *J* = 1.1 Hz, 2H), 7.95 (d, *J* = 1.1 Hz, 2H), 5.47 (s, 2H), 1.68 (s, 18H), 1.60 (s, 18H), 1.07 (d, *J* = 1.7 Hz, 42H). ^{13}C NMR (126 MHz, chloroform-*d*) δ 152.30, 152.18, 143.30, 143.09, 138.88, 134.10, 133.89, 133.51, 128.27, 124.72, 123.17, 119.77, 119.54, 117.08, 115.83, 104.90, 83.03, 42.31, 32.28, 32.15, 18.65. HRMS (APPI+): $C_{70}H_{89}S_2Si_2$ $[M+H]^+$ 1049.5944; found 1049.5938.

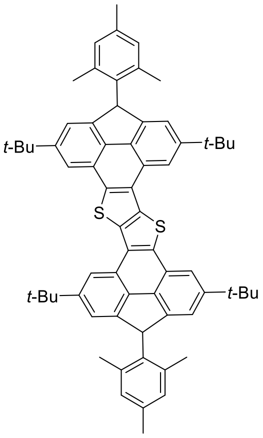

Compound 17-Mes: Dry THF (50 mL) and compound **13** (400 mg, 0.56 mmol) were added to a round-bottom flask under inert atmosphere and cooled at -78 °C. 2-mesitylmagnesium bromide 1.0 M in THF (4.78 mL) was added dropwise and the resulting mixture was stirred for 30 min at -78 °C. The mixture was stirred for 16 h at room temperature. 15 mL of DCM and 20 mL of water was added to the flask and the organic solvent was removed under reduced pressure, leading to a suspension of white solid in water. The crude product was filtered and rinsed several times with MeOH. 350 mg of the resulting powder and 60 mL of dichloromethane were added in a round-bottom flask at 0 °C. Triethylsilane (0.47 mL, 2.57 mmol) and boron trifluoride diethyl etherate (0.36 mL, 2.8 mmol) were added and the resulting mixture was stirred at 0 °C for an hour, then 12 hours at room temperature. The reaction was quenched with $NaHCO_3$ aq., and the organic solvent was removed under reduced pressure. The crude product was filtered, rinsed with clean water and triturated with a solution of AcOEt/Hexanes (3:7 v/v) to afford the desired compound as a brown solid (285 mg, 55 ^{1}H NMR (500 MHz, cdcl$_3$) δ 8.52 (s, 1H), 8.51 (s, 1H), 8.26 – 8.04 (m, 2H), 7.62 (d, *J* = 1.2 Hz, 2H), 7.56 (d, *J* = 1.2 Hz, 2H), 7.15 (s, 2H), 6.73 (s, 2H), 6.12 (s, 1H), 6.09 (s, 1H), 2.85 (s, 3H), 2.83 (s, 3H), 2.37 (s, 6H), 1.64 (d, *J* = 1.5 Hz, 18H), 1.56 (d, *J* = 1.6 Hz, 18H), 1.05 (s, 3H), 1.02 (s, 3H).^{13}C NMR (126 MHz, cdcl$_3$) δ 152.05, 152.02, 151.94, 151.92, 145.89, 145.84,

145.66, 145.60, 138.96, 138.94, 138.51, 137.64, 137.62, 136.23, 134.42, 134.39, 133.95, 133.92, 133.84, 133.82, 133.25, 130.44, 130.42, 128.91, 128.35, 128.32, 124.70, 123.19, 118.89, 118.66, 118.64, 116.50, 115.20, 52.29, 36.09, 36.07, 32.41, 32.40, 32.26, 21.99, 21.97, 21.96, 20.95, 19.18, 19.09. Some carbon signals are duplicated due to the presence of two isomers. HRMS (APPI+): $C_{66}H_{69}S_2$ $[M+H]^+$ 925.4841; found 925.4835.

Experimental Characterization

Compound 3:

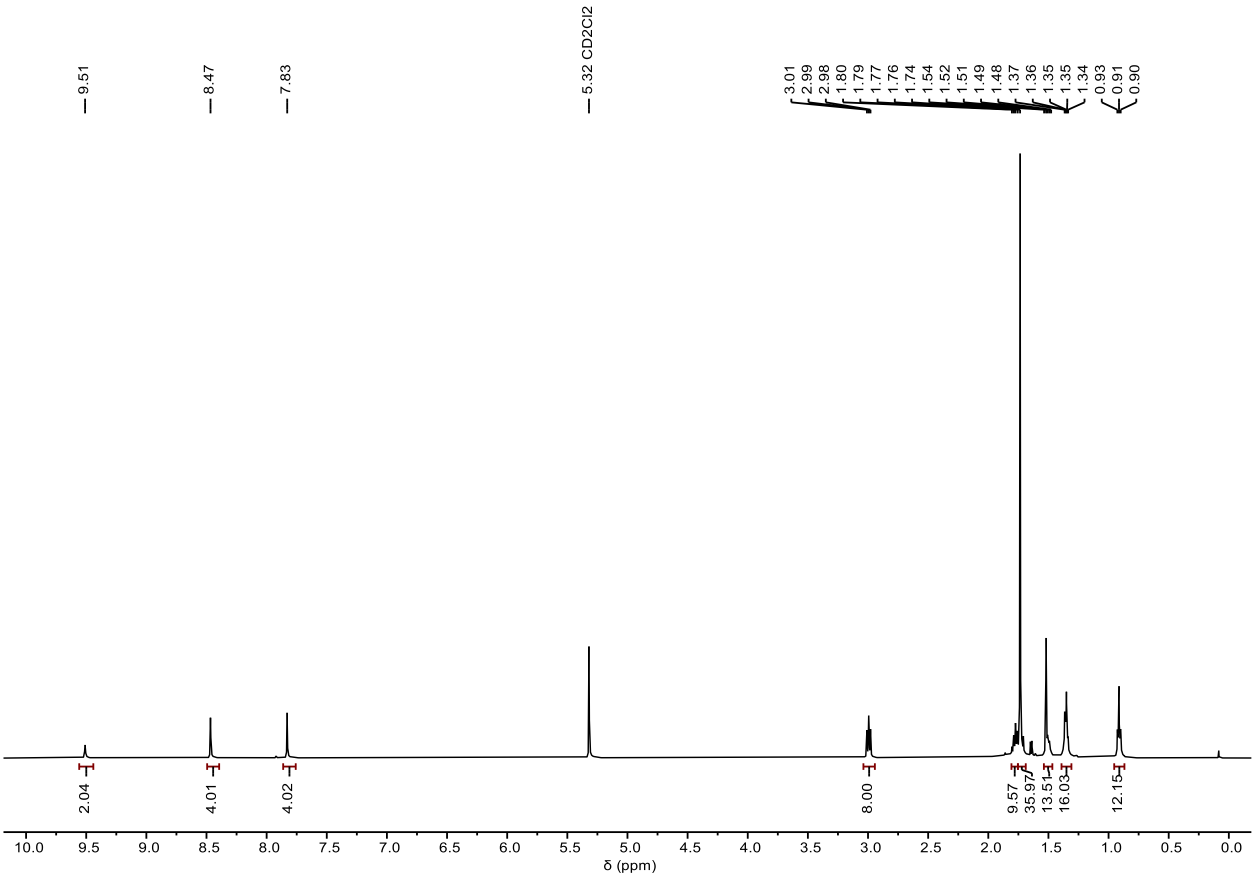

Figure S1. ^{1}H NMR (500 MHz) of compound **3** in CD_2Cl_2.

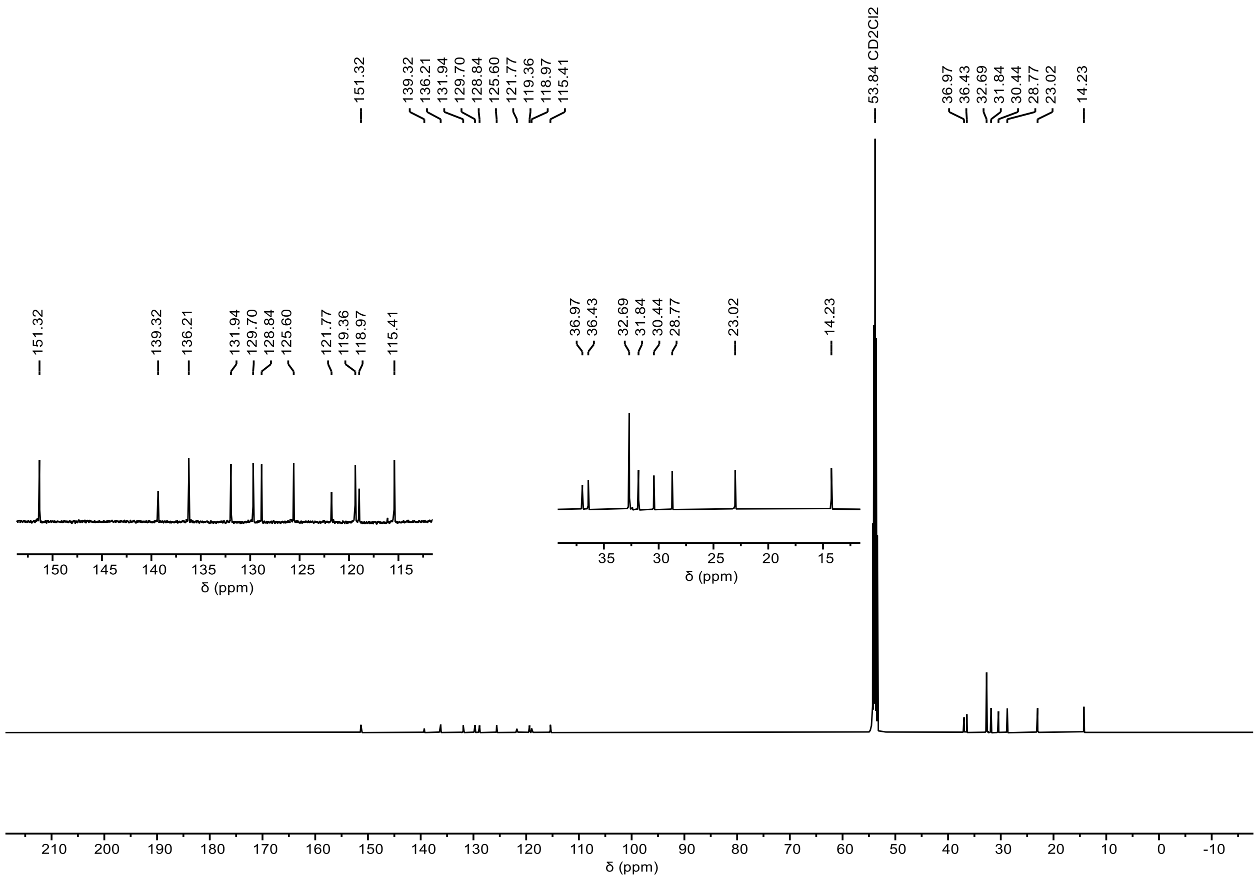

Figure S2. ^{13}C NMR (126 MHz) of compound **3** in CD_2Cl_2.

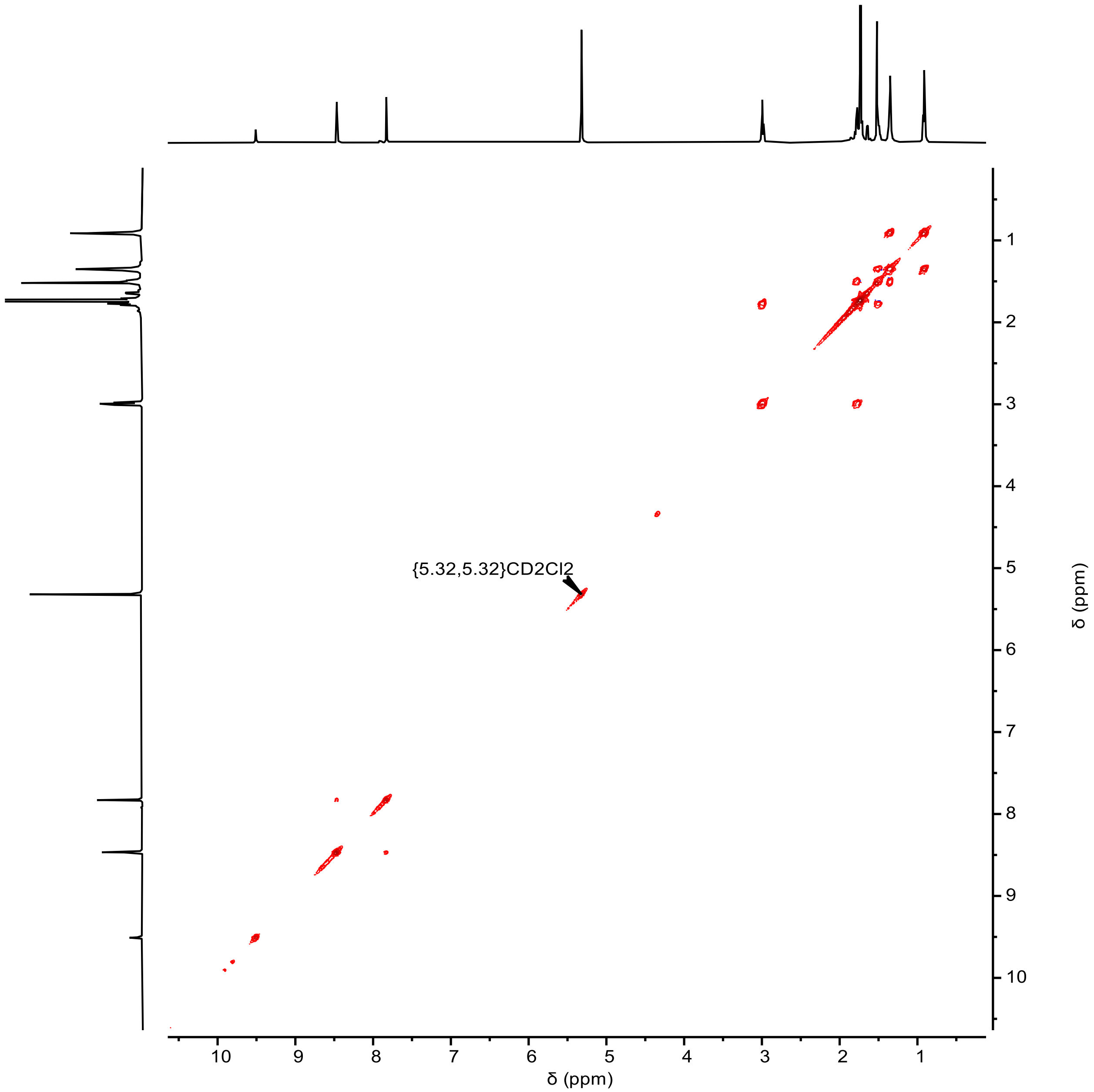

Figure S3. ^{1}H-^{1}H COSY NMR of compound **3** in CD_2Cl_2.

Compound 4:

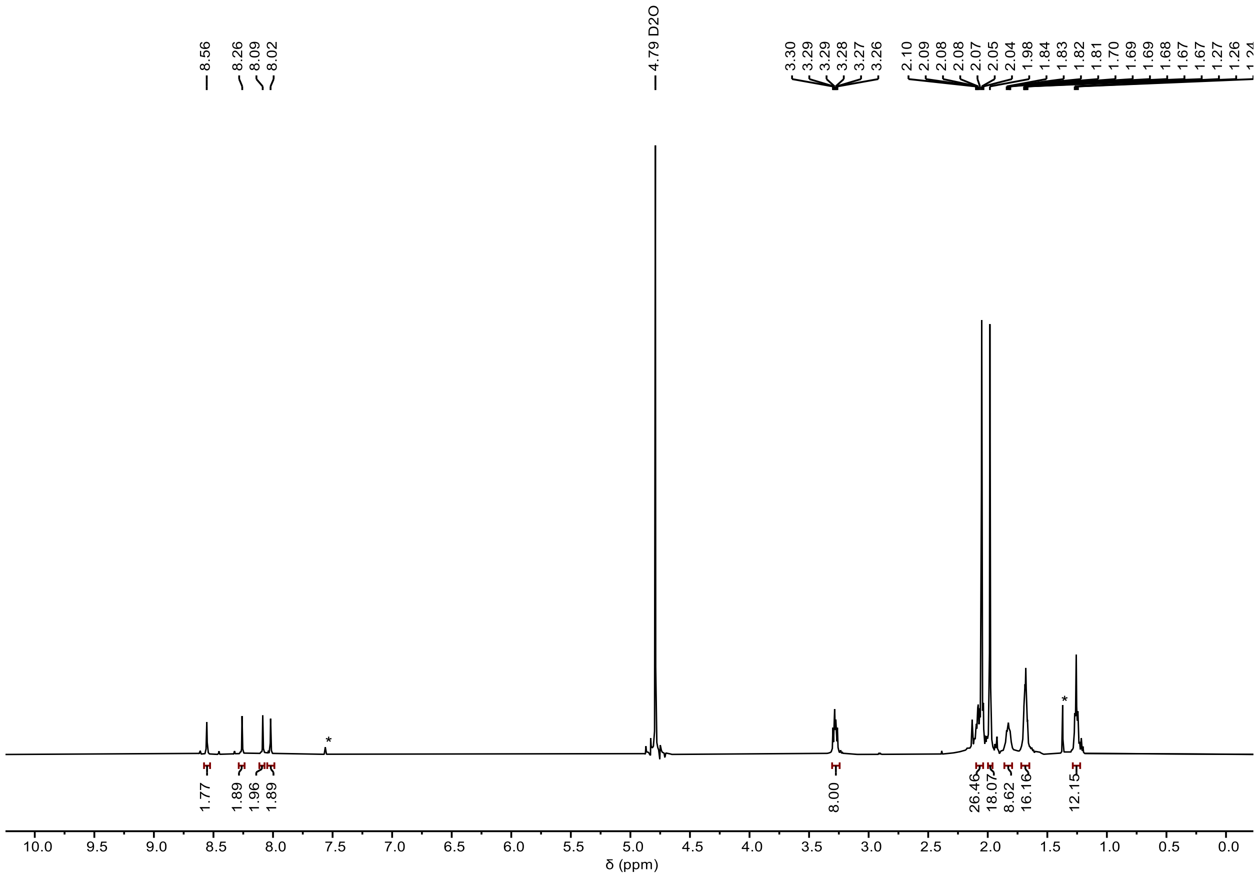

Figure S4. ^{1}H NMR (500 MHz) of compound **4** in CS_2. * indicates residual impurities from the carbon disulfide (see blank below).

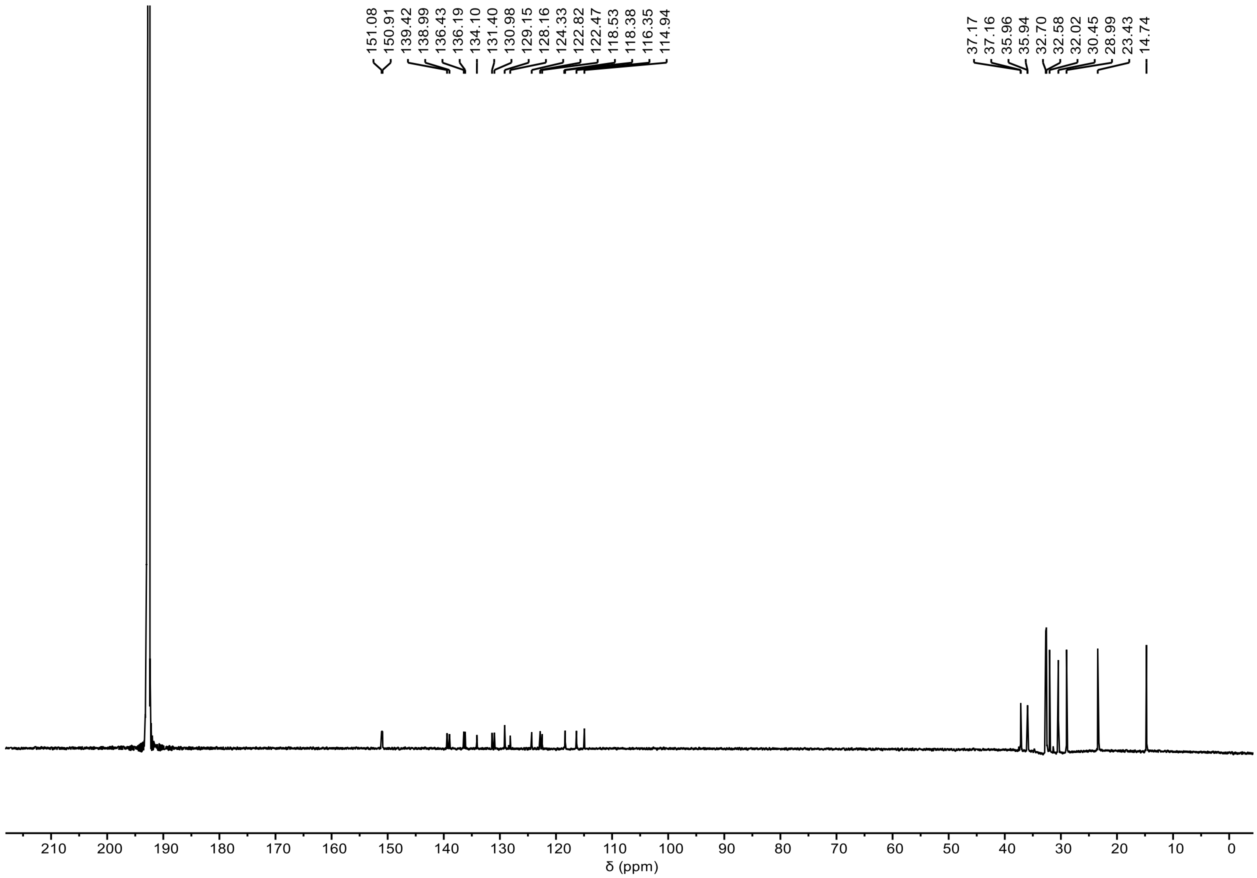

Figure S5. ^{13}C NMR (126 MHz) of compound **4** in CS_2. Referenced against CS_2 carbon at 192.58 ppm.

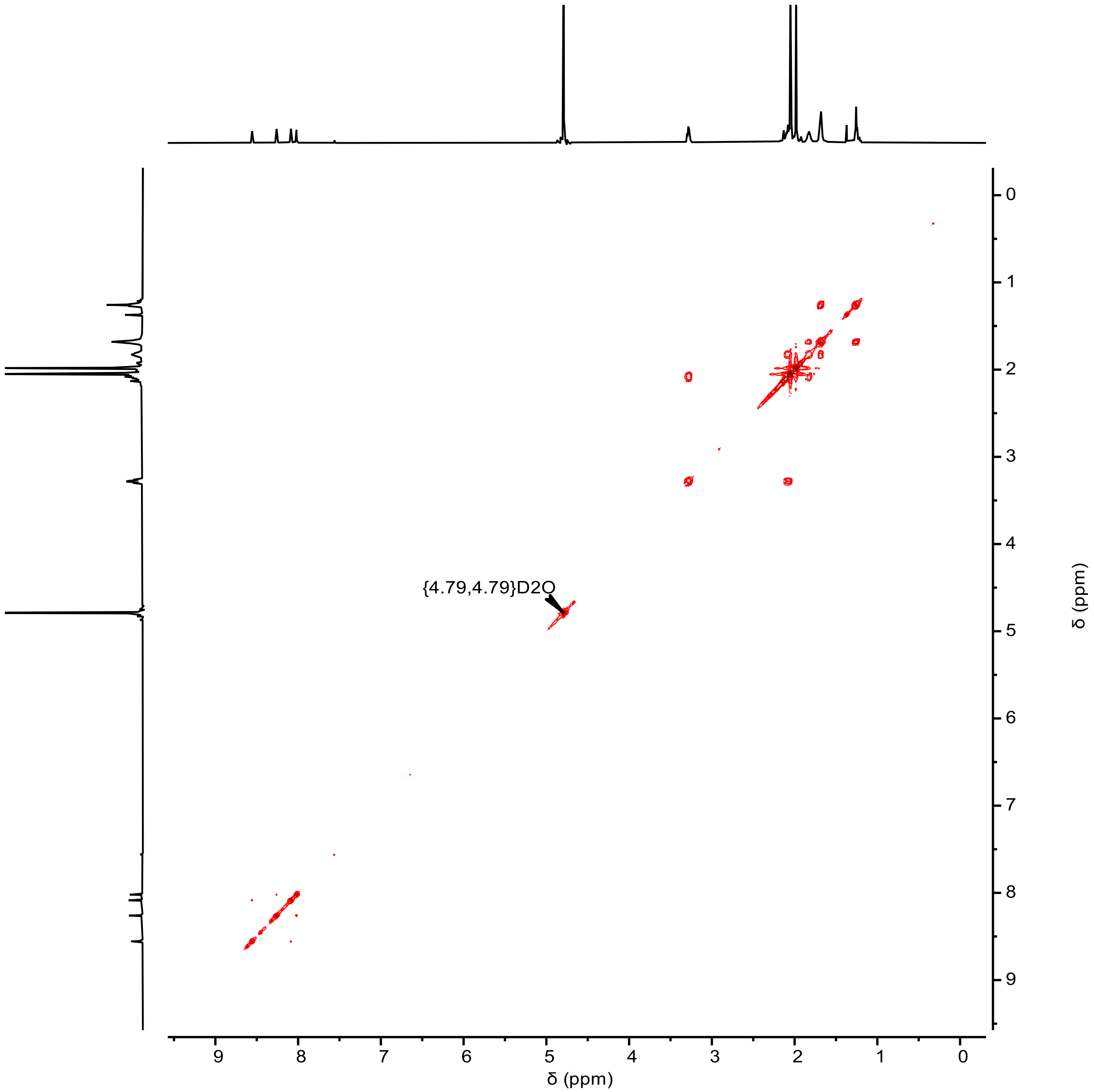

Figure S6. ^{1}H-^{1}H COSY NMR of compound **4** in CS_2.

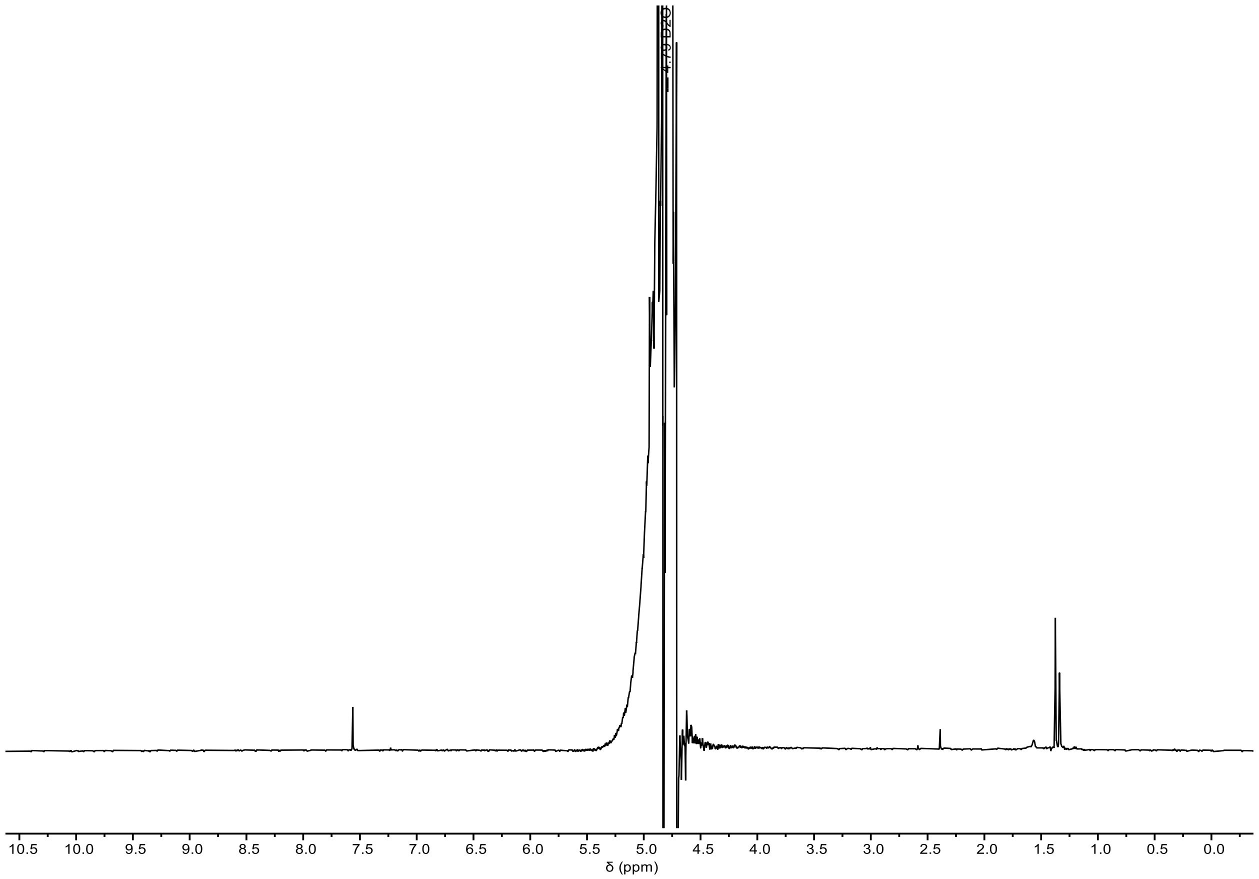

Figure S7. ^{1}H NMR (500 MHz) of blank CS_2 with a D_2O lock tube.

Compound 5:

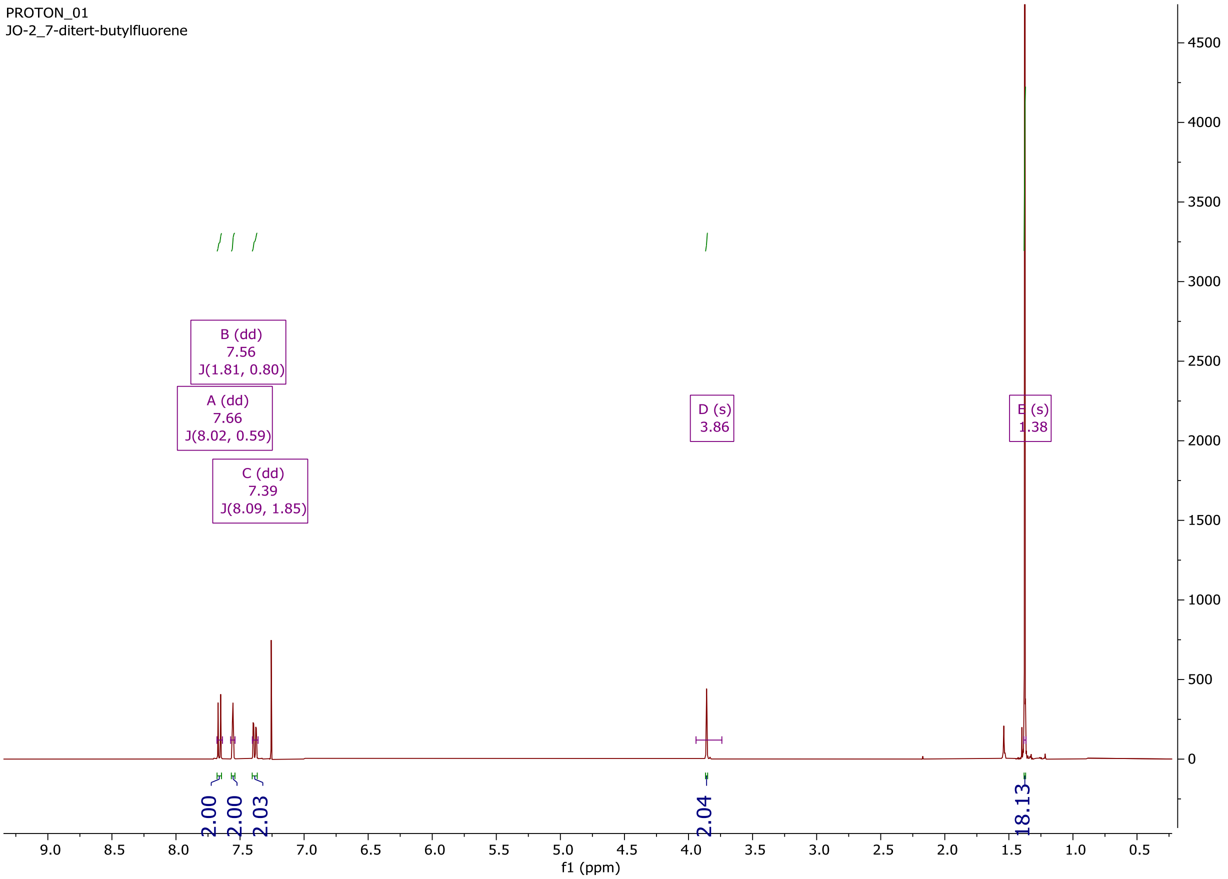

Figure S8. ^{1}H NMR (400 MHz) of compound **5** in $CDCl_3$.

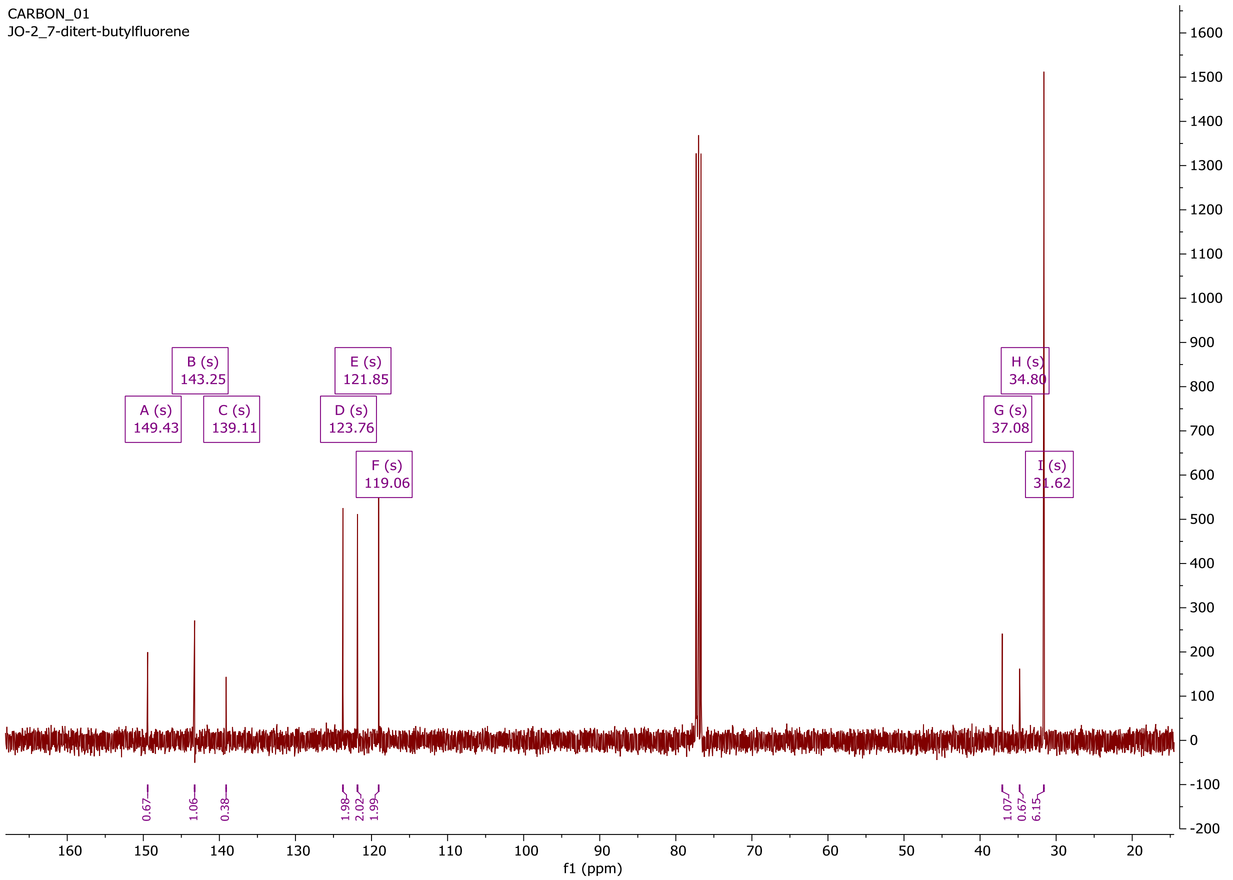

Figure S9. ^{13}C NMR (101 MHz) of compound **5** in $CDCl_3$.

Compound 6:

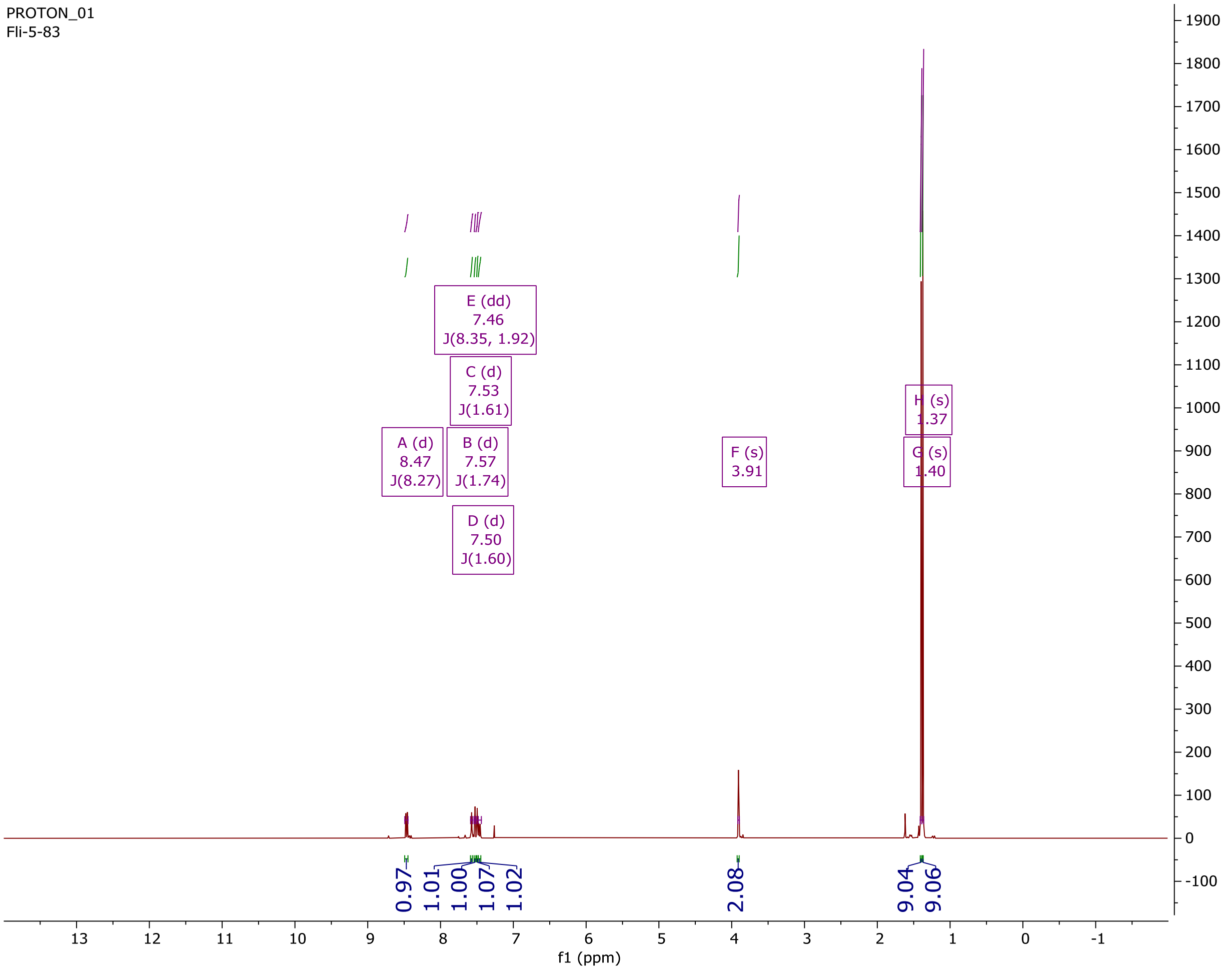

Figure S10. ^{1}H NMR (400 MHz) of compound **6** in $CDCl_3$.

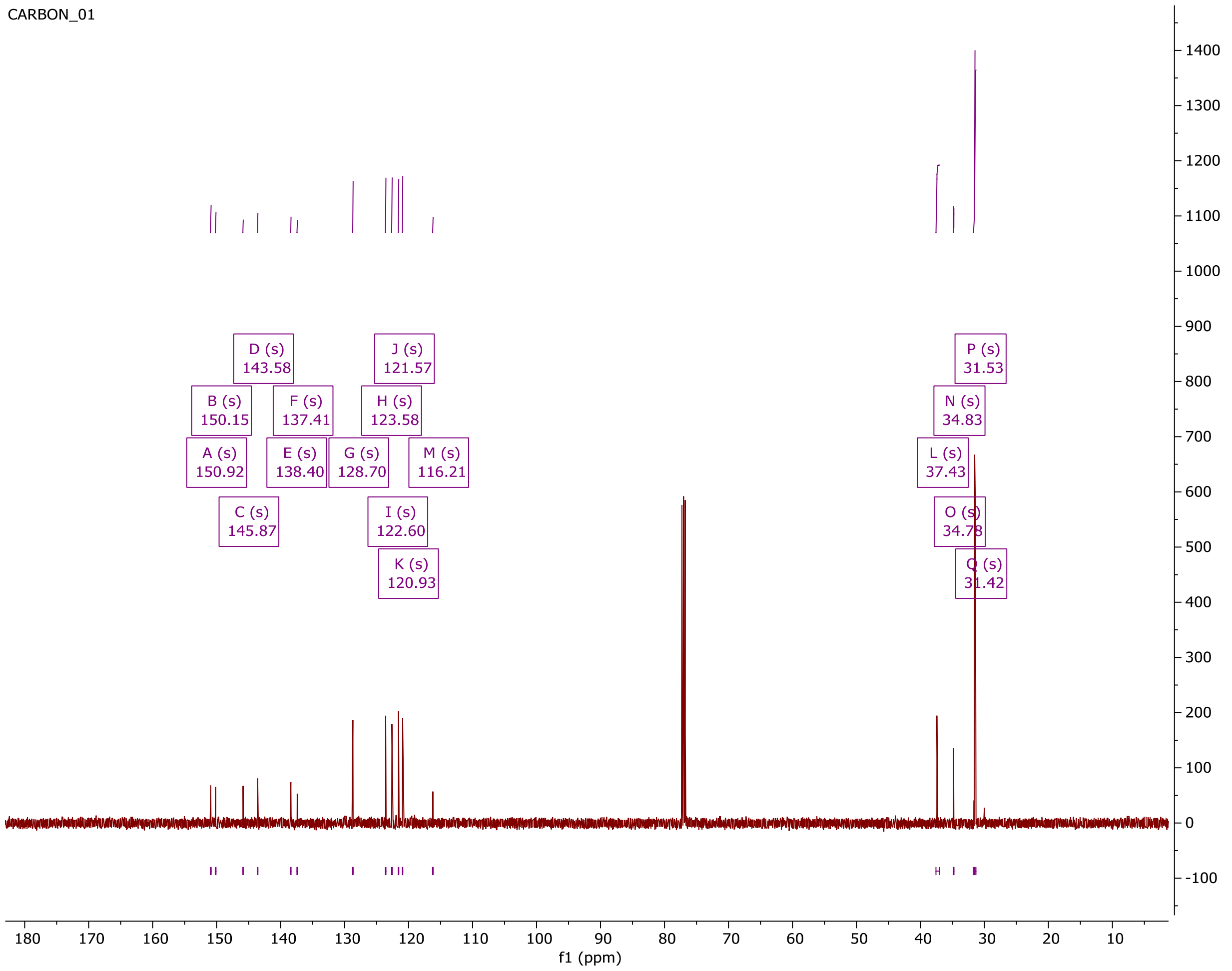

Figure S11. ^{13}C NMR (126 MHz) of compound **6** in $CDCl_3$.

Compound 7:

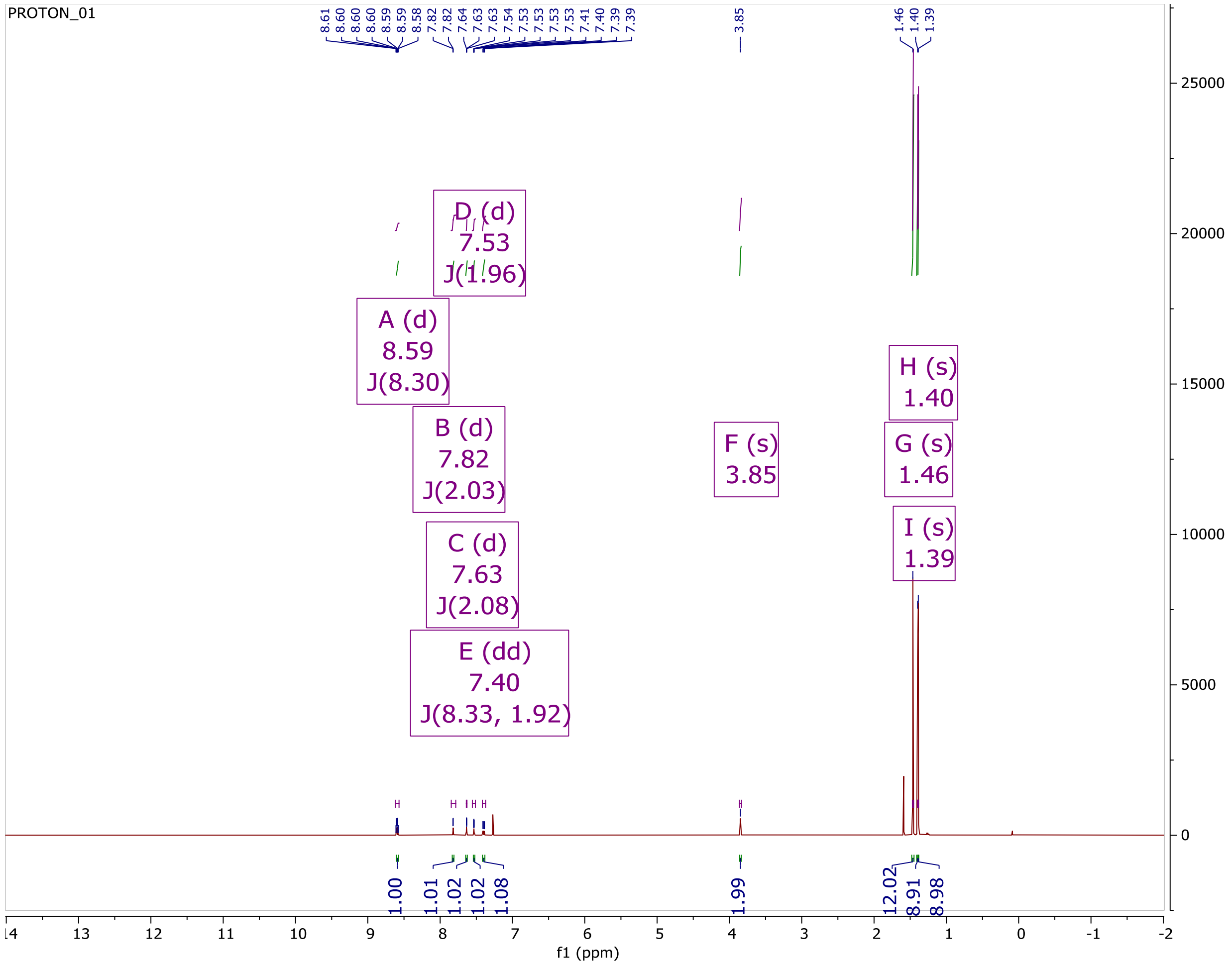

Figure S12. ^{1}H NMR (500 MHz) of compound **7** in $CDCl_3$.

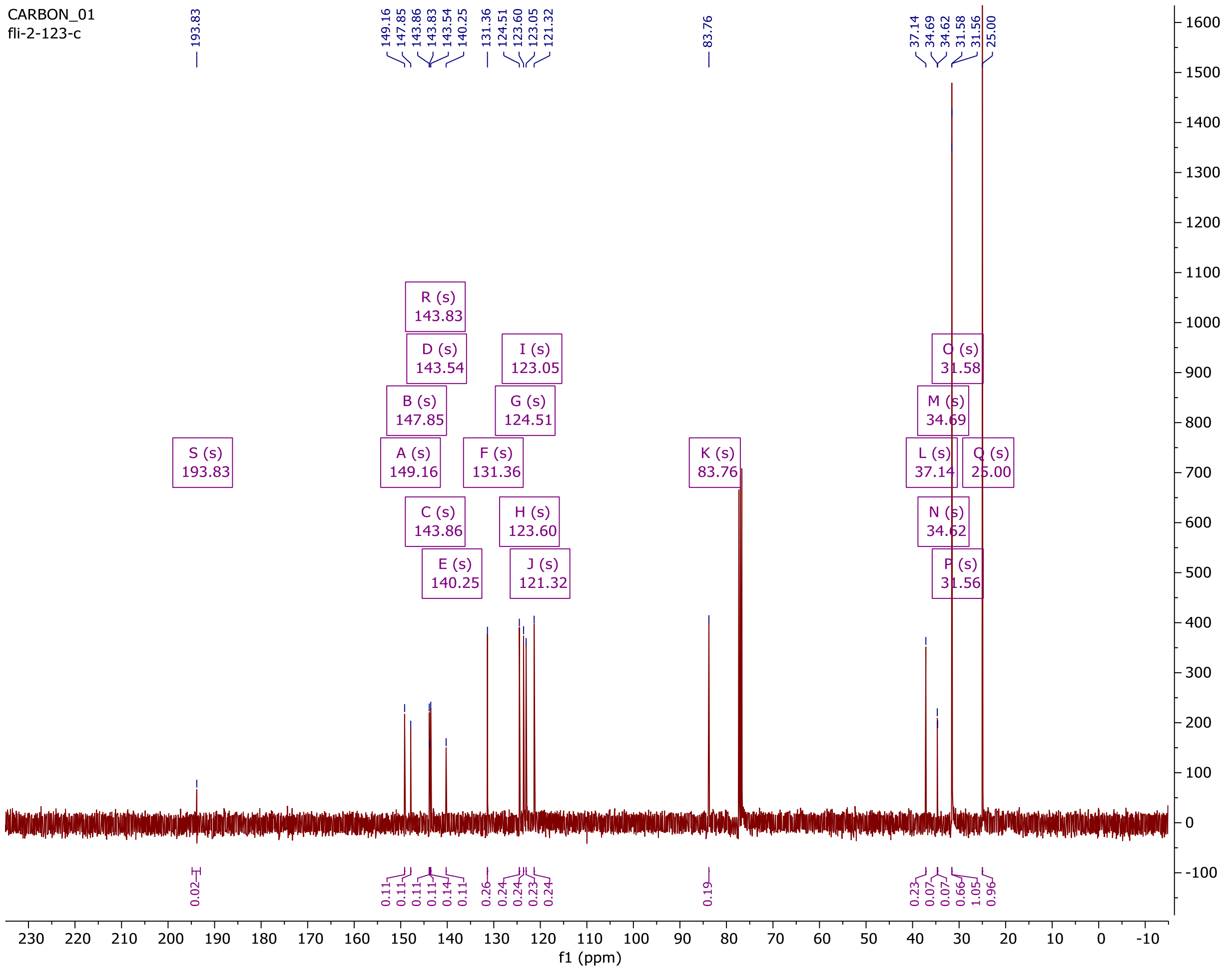

Figure S13. ^{13}C NMR (101 MHz) of compound **7** in $CDCl_3$.

Compound 8:

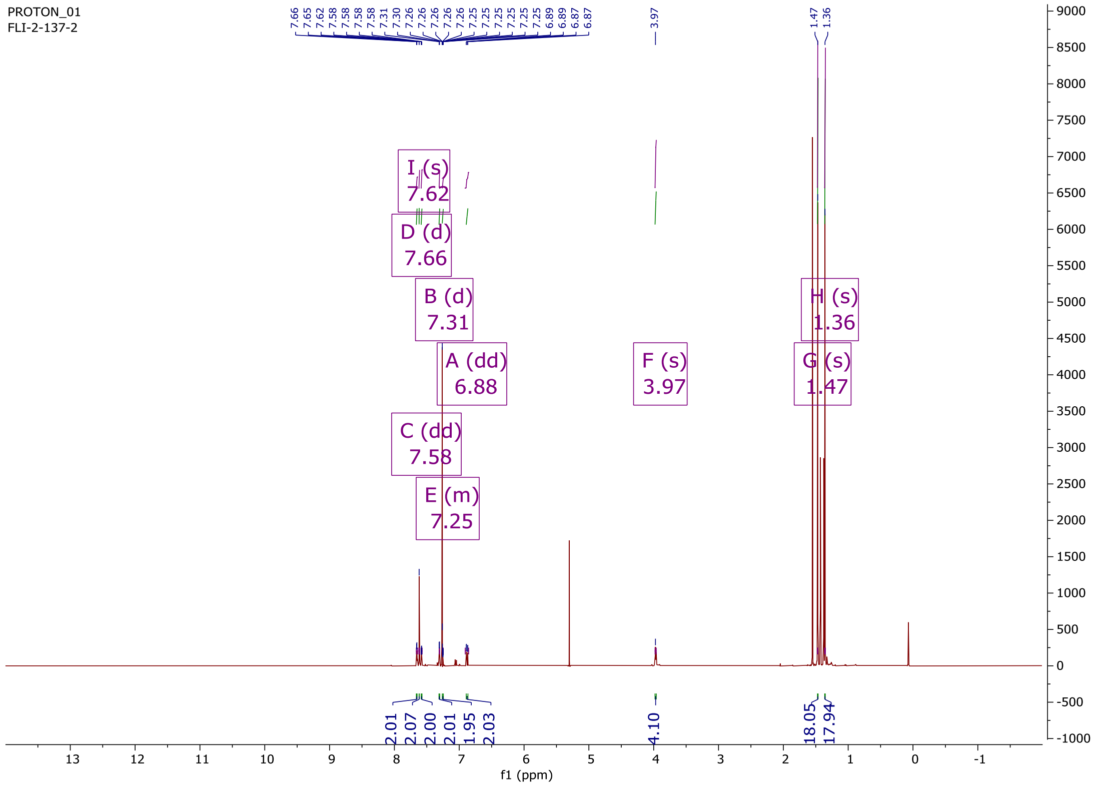

Figure S14. ^{1}H NMR (400 MHz) of compound **8** in $CDCl_3$.

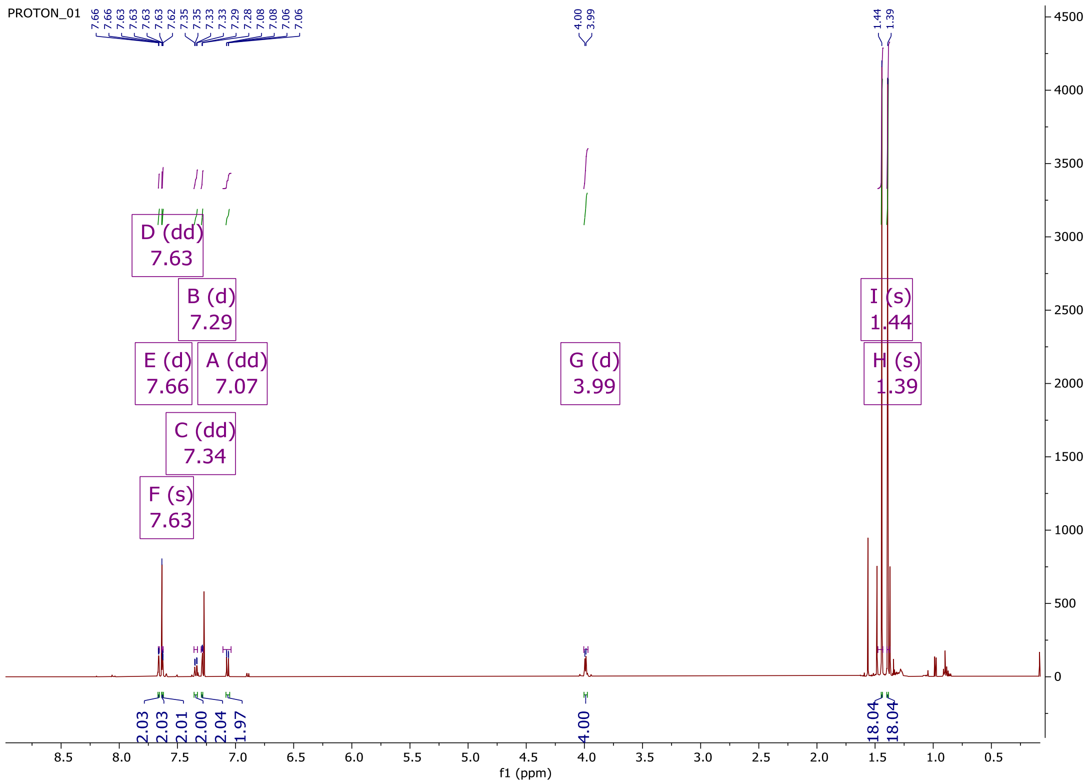

Figure S15. ^{1}H NMR (500 MHz) of compound **8** in $CDCl_3$.

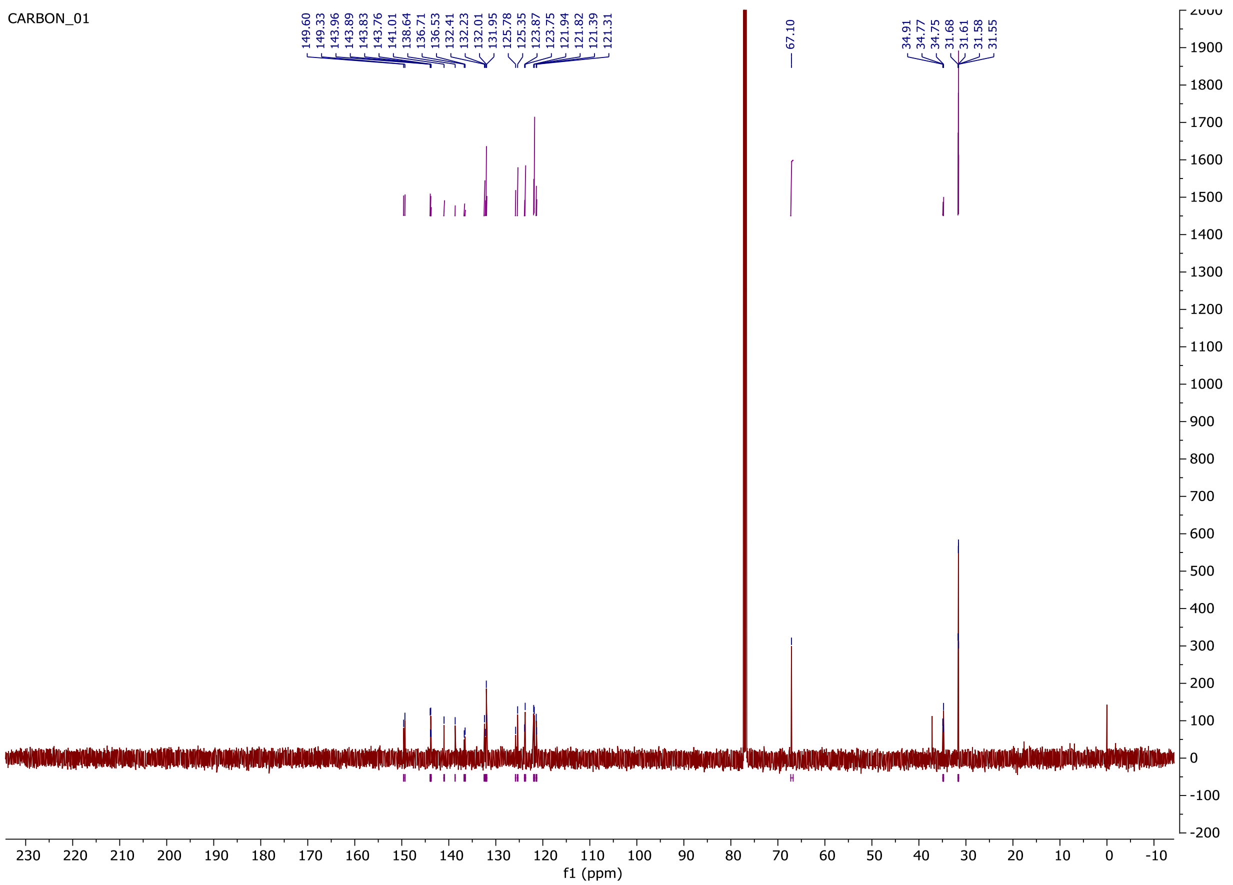

Figure S16. ^{13}C NMR (126 MHz) of compound **8** in $CDCl_3$.

Compound 9:

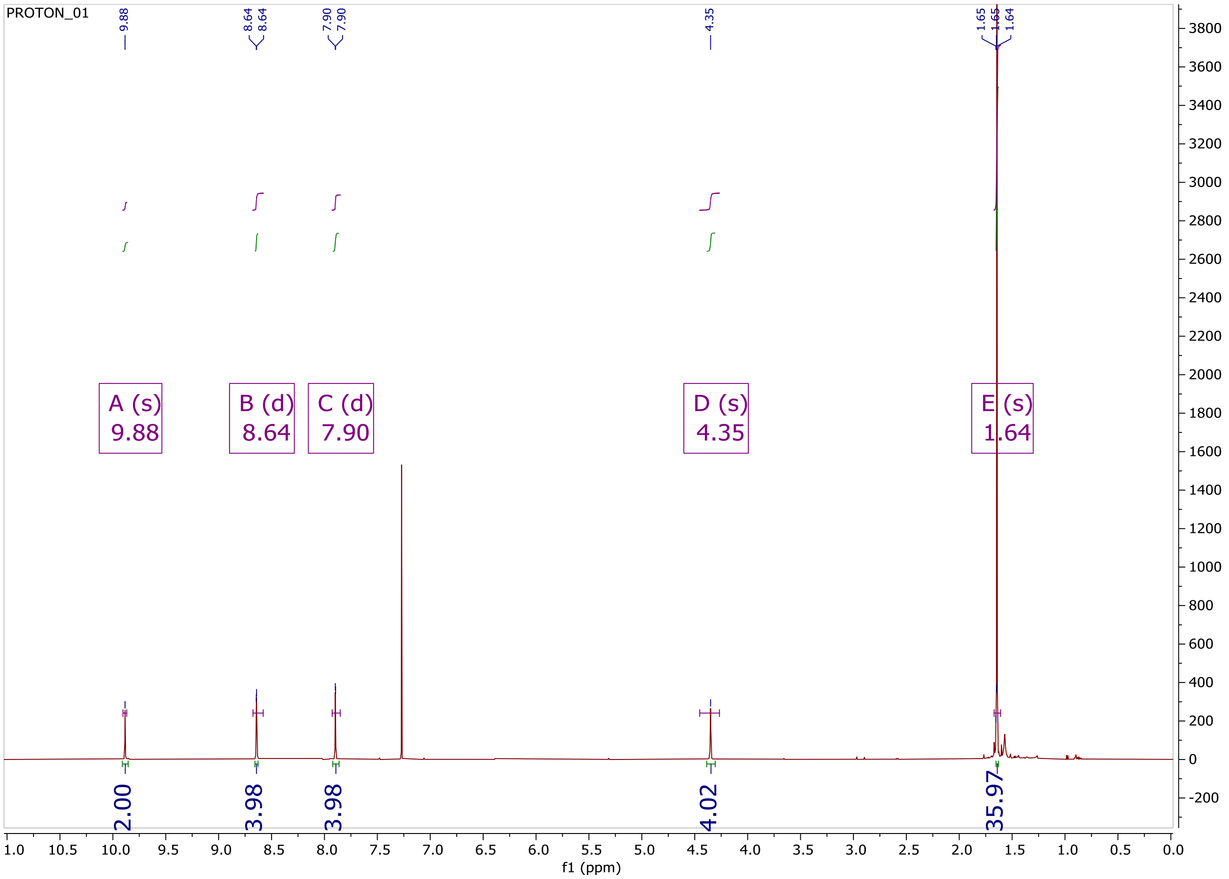

Figure S17. ^{1}H NMR (500 MHz) of compound **9** in $CDCl_3$.

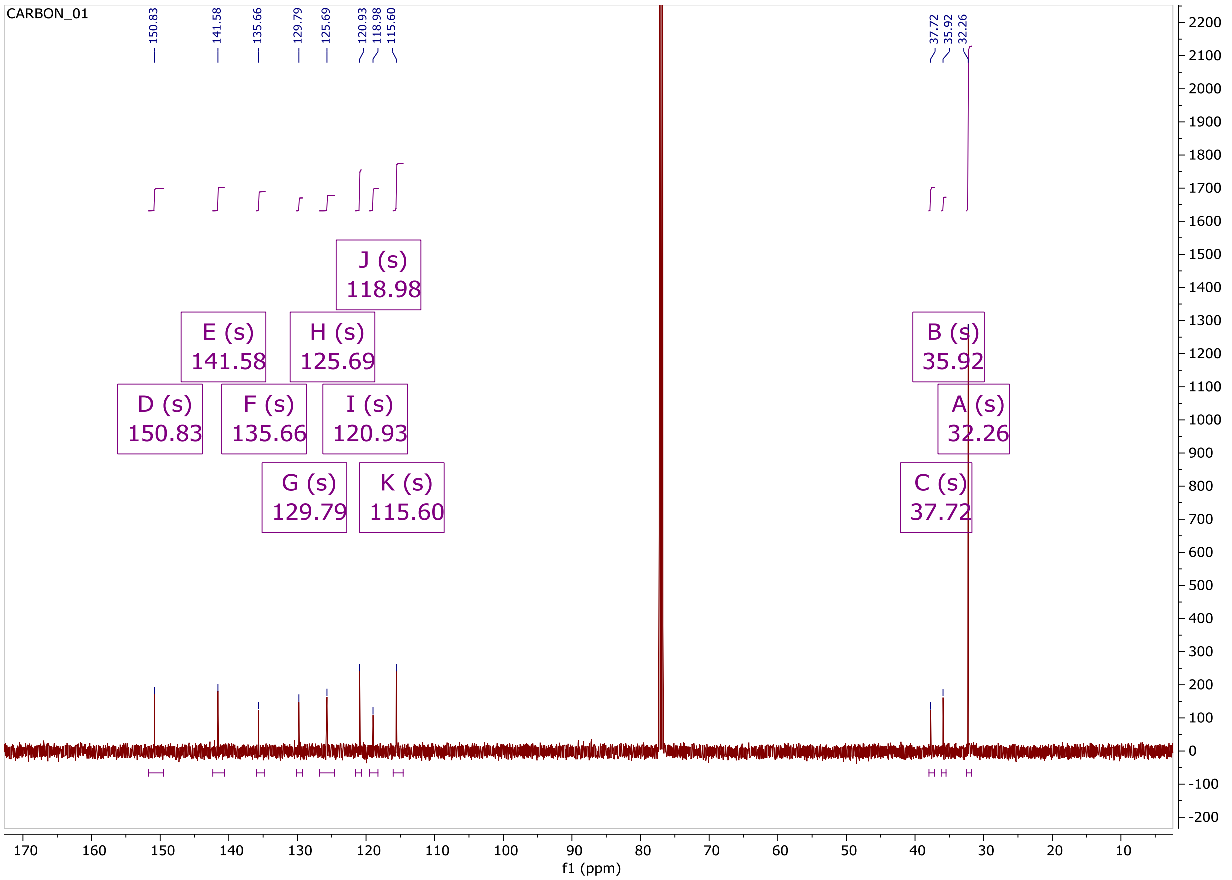

Figure S18. ^{13}C NMR (126 MHz) of compound **9** in $CDCl_3$.

Compound 10:

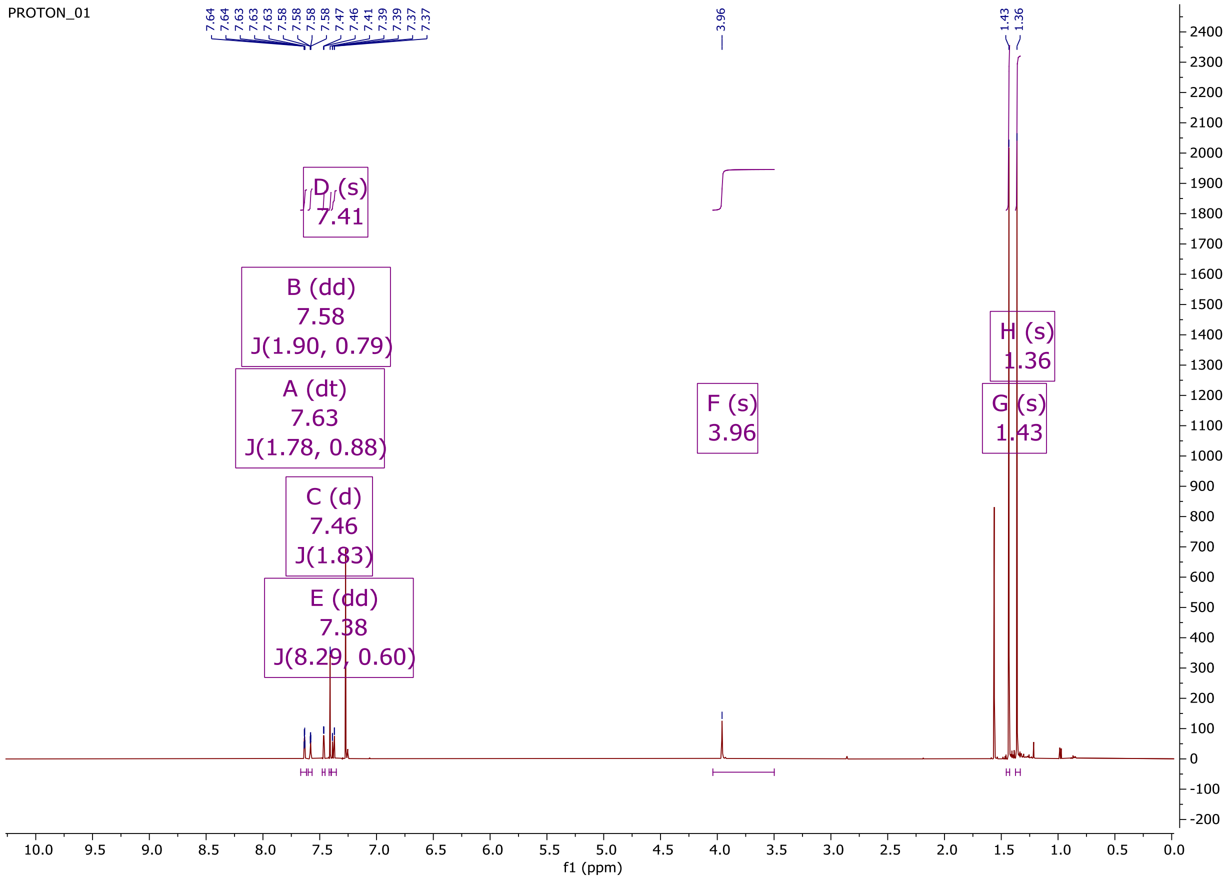

Figure S19. ^{1}H NMR (500 MHz) of compound **10** in $CDCl_3$.

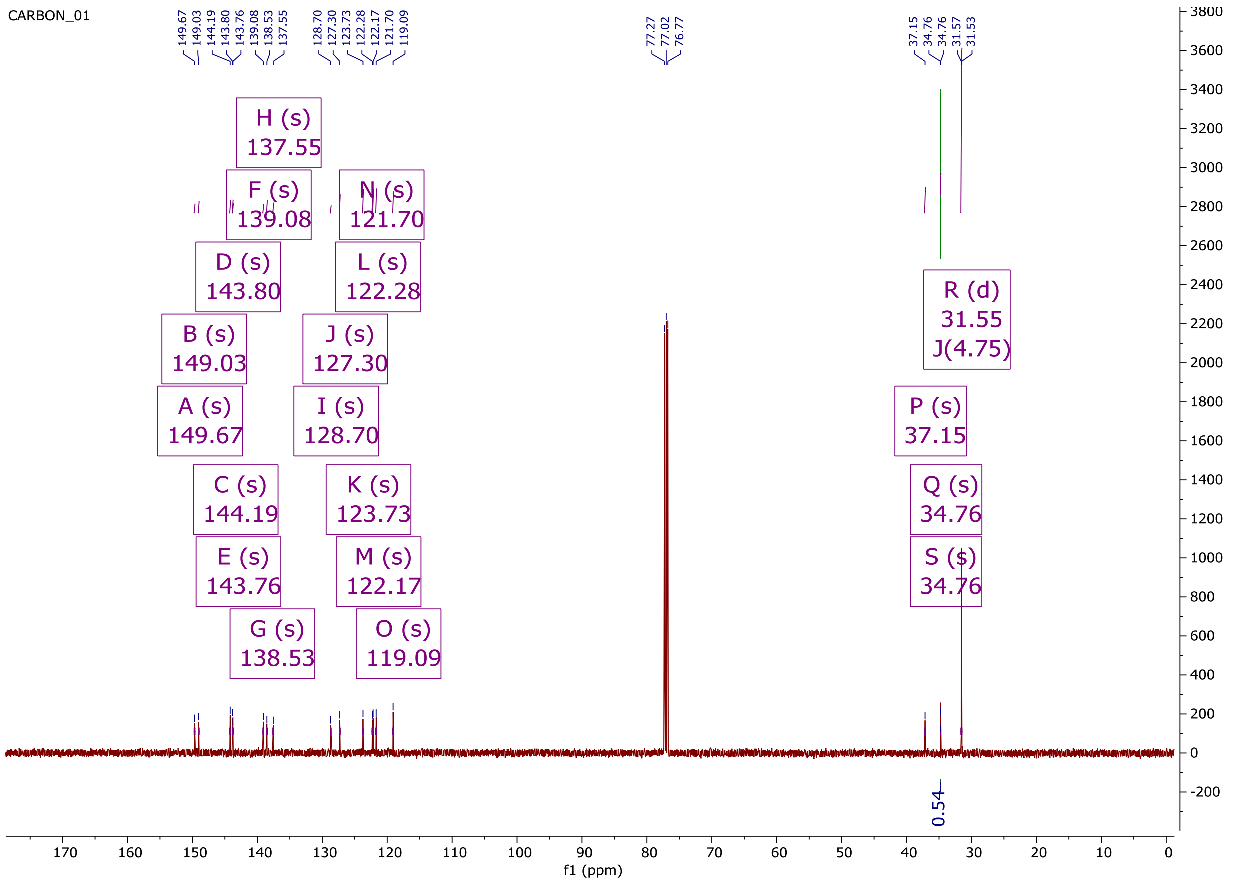

Figure S20. ^{13}C NMR (126 MHz) of compound **10** in $CDCl_3$.

Compound 11:

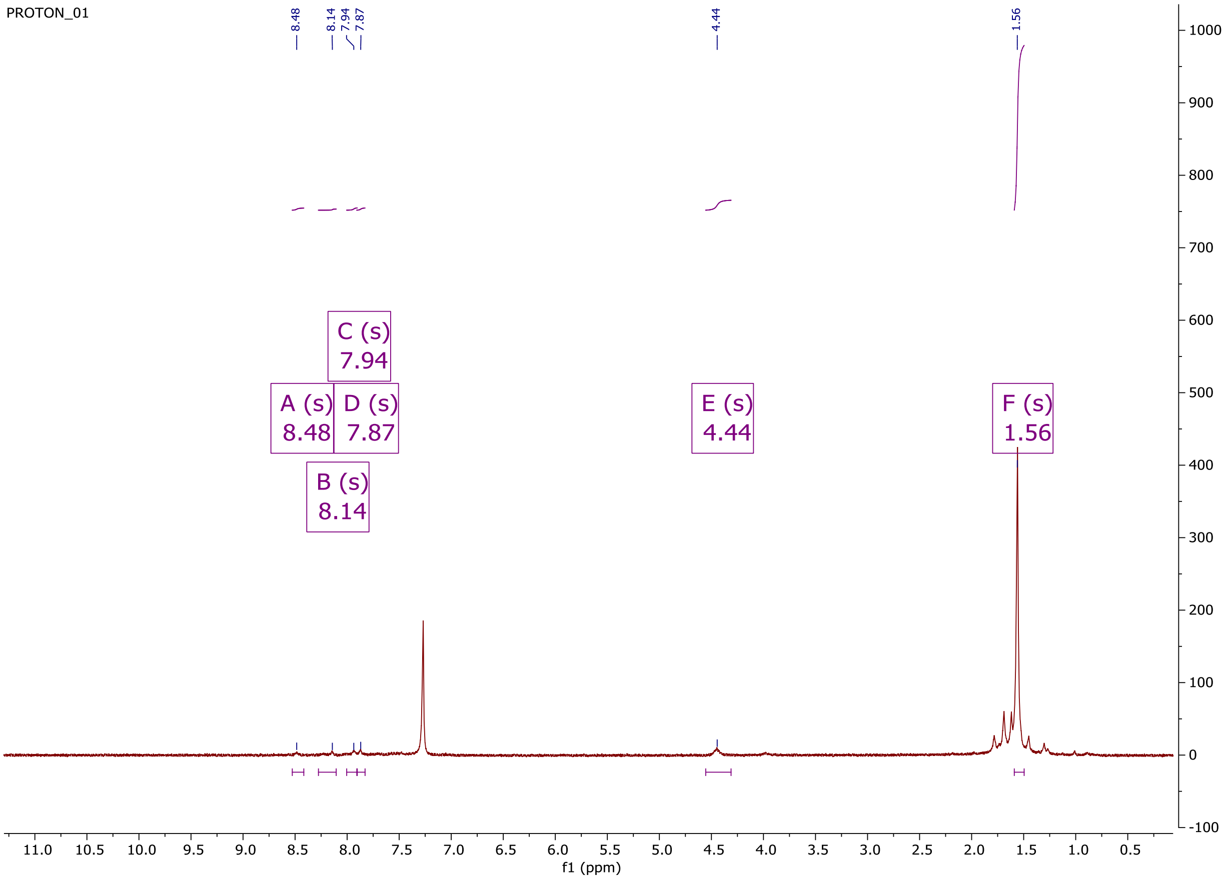

Figure S21. ^{1}H NMR (500 MHz) of compound **11** in $CDCl_3$.

Compound 14:

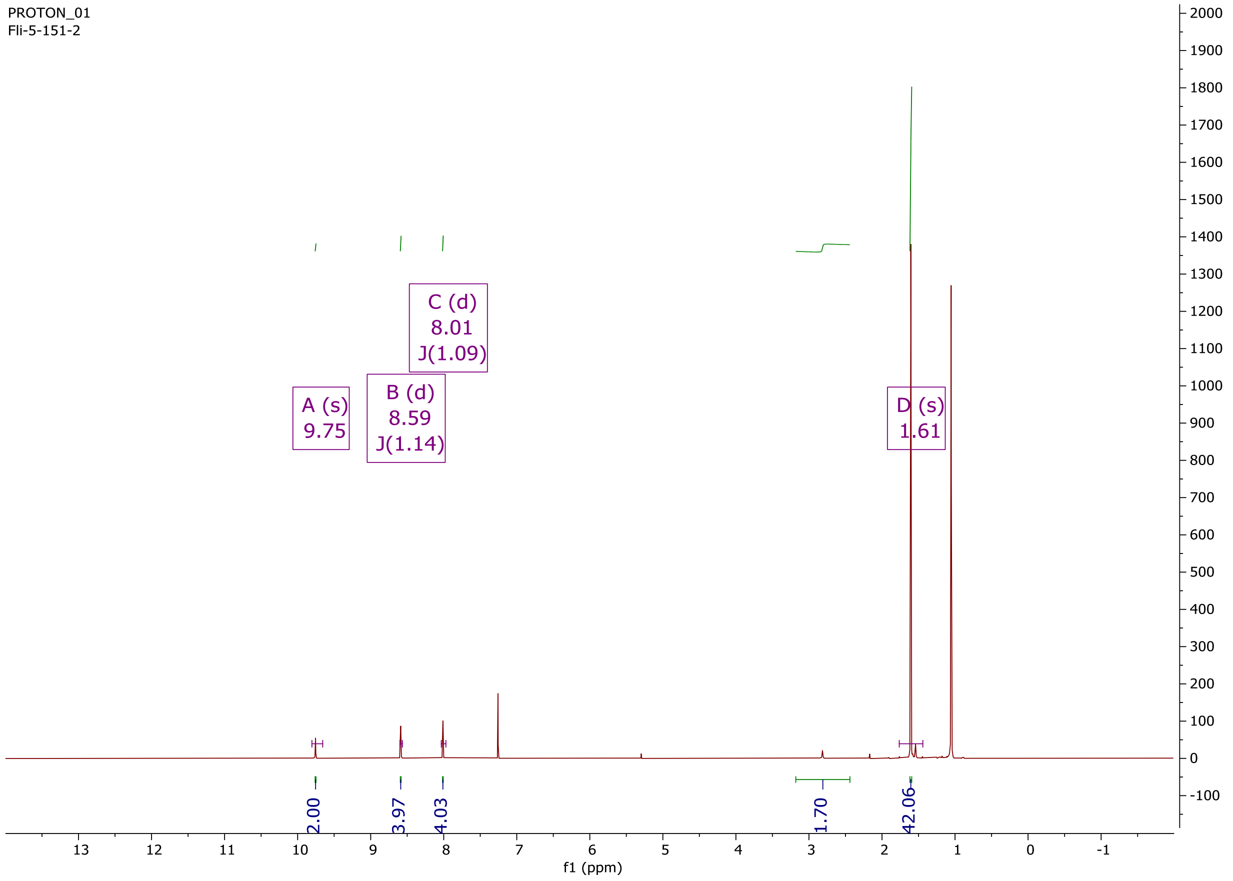

Figure S22. ^{1}H NMR (400 MHz) of compound **14** in $CDCl_3$.

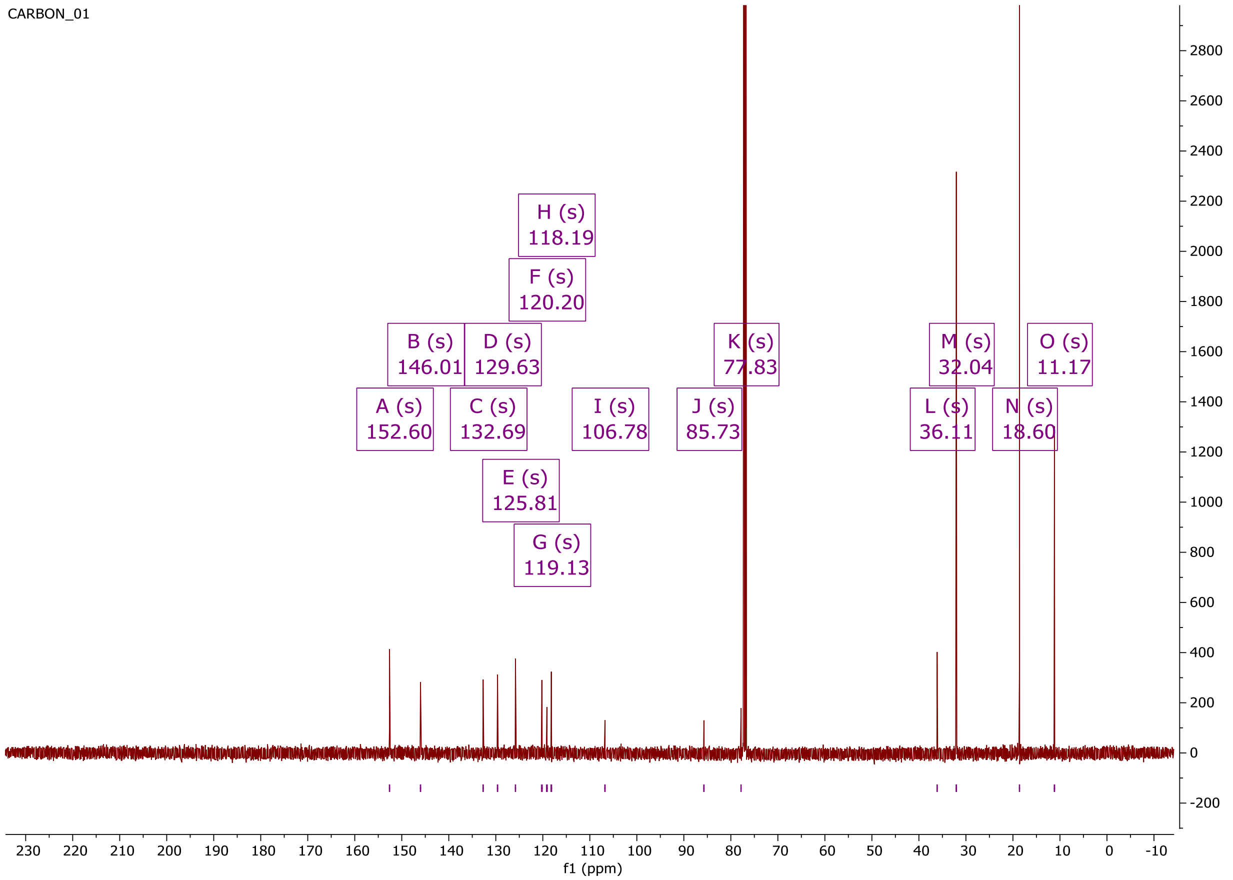

Figure S23. ^{13}C NMR (126 MHz) of compound **14** in $CDCl_3$.

Compound 15:

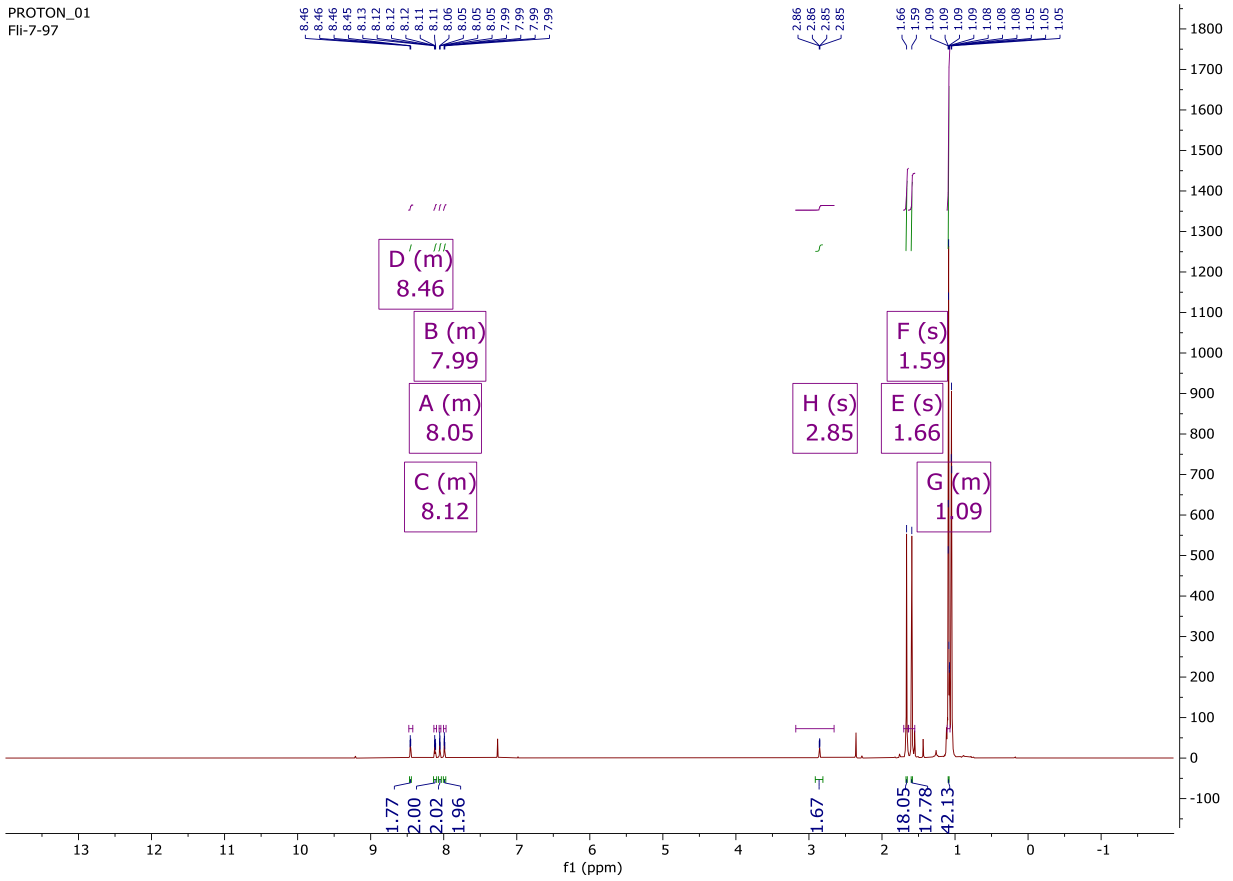

Figure S24. ^{1}H NMR (400 MHz) of compound **15** in $CDCl_3$.

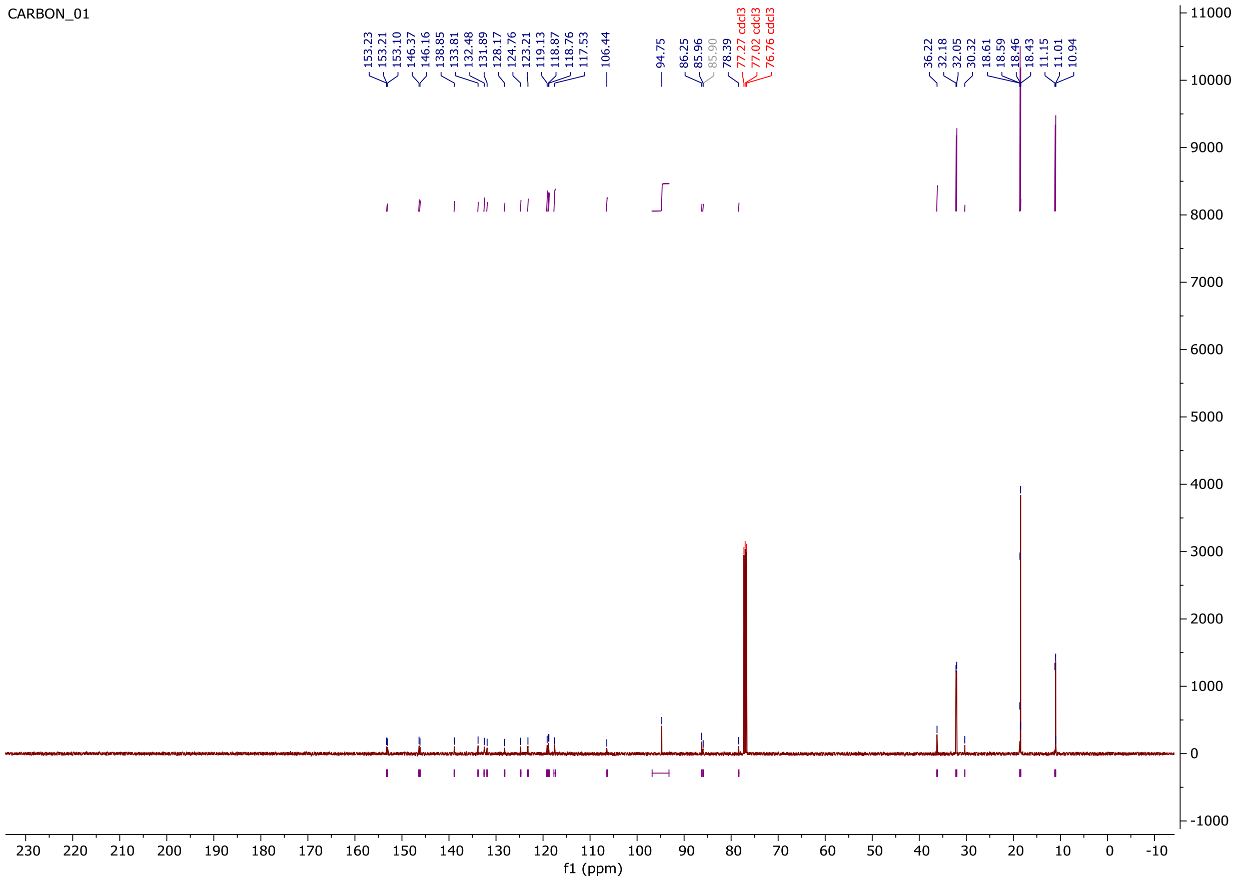

Figure S25. ^{13}C NMR (126 MHz) of compound **15** in $CDCl_3$.

Compound 16-Tips:

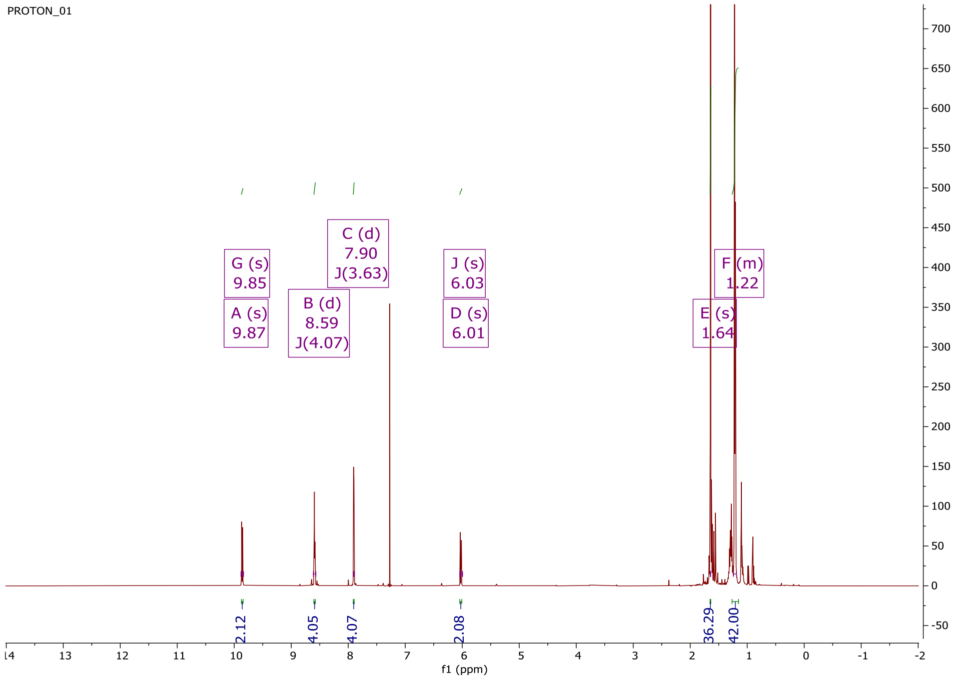

Figure S26. ^{1}H NMR (500 MHz) of compound **16-Tips** in $CDCl_3$.

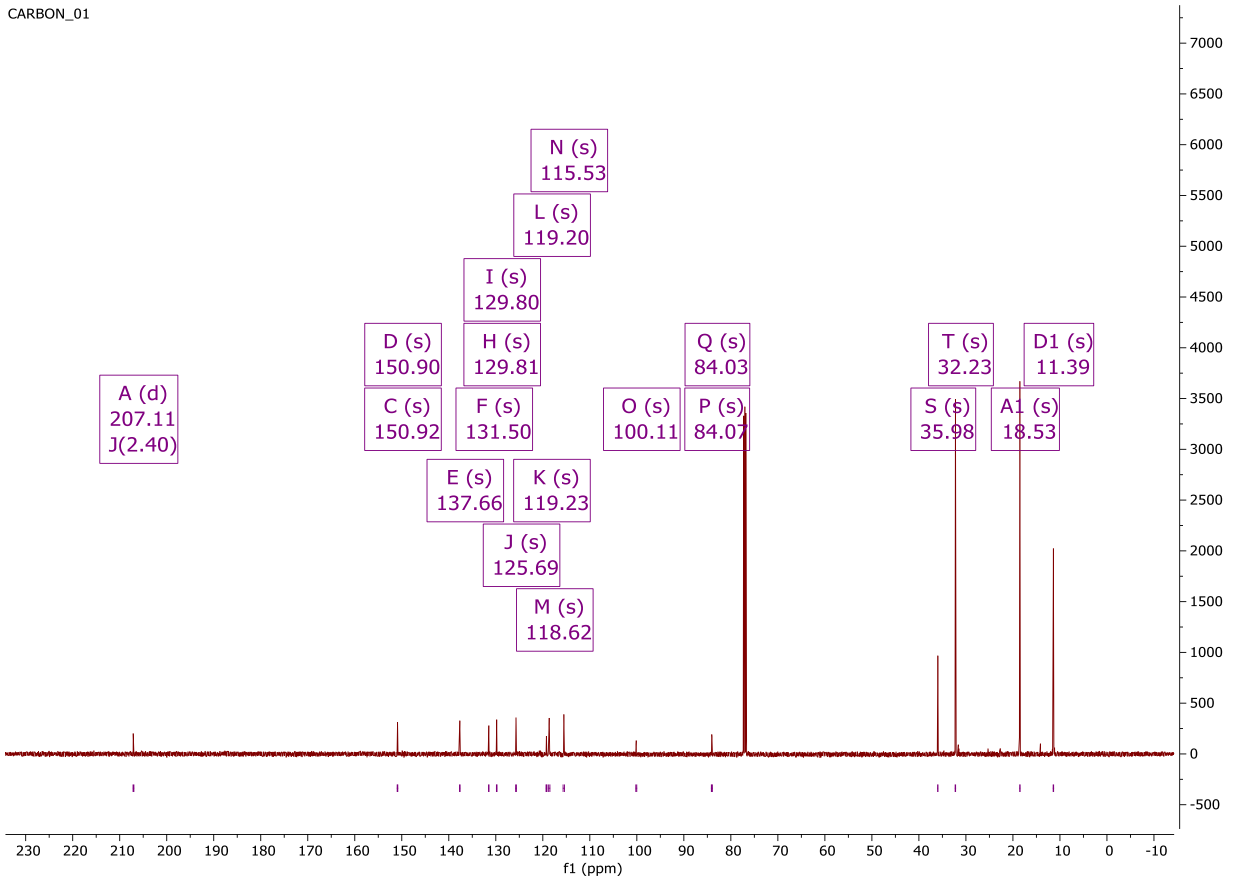

Figure S27. ^{13}C NMR (126 MHz) of compound **16-Tips** in $CDCl_3$.

Compound 16-Mes:

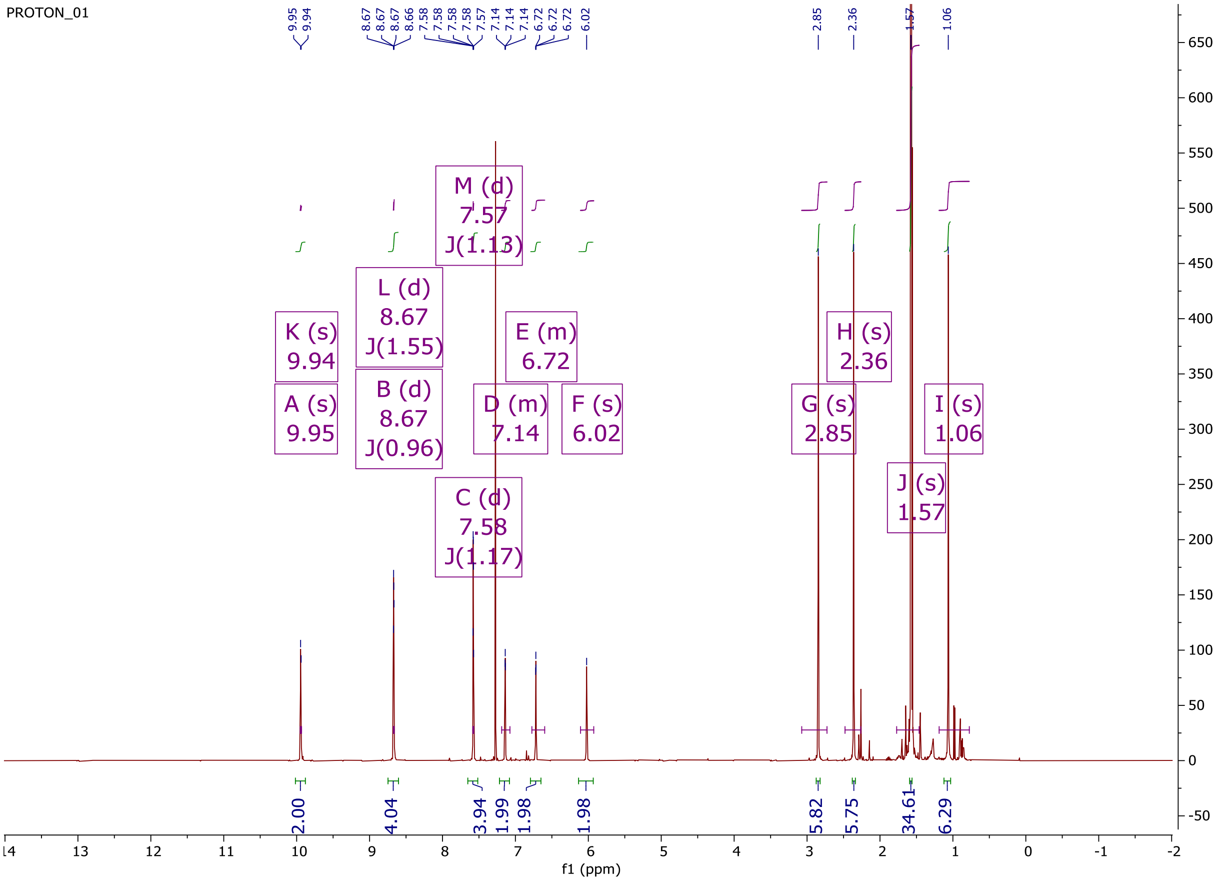

Figure S28. ^{1}H NMR (500 MHz) of compound **16-Mes** in $CDCl_3$.

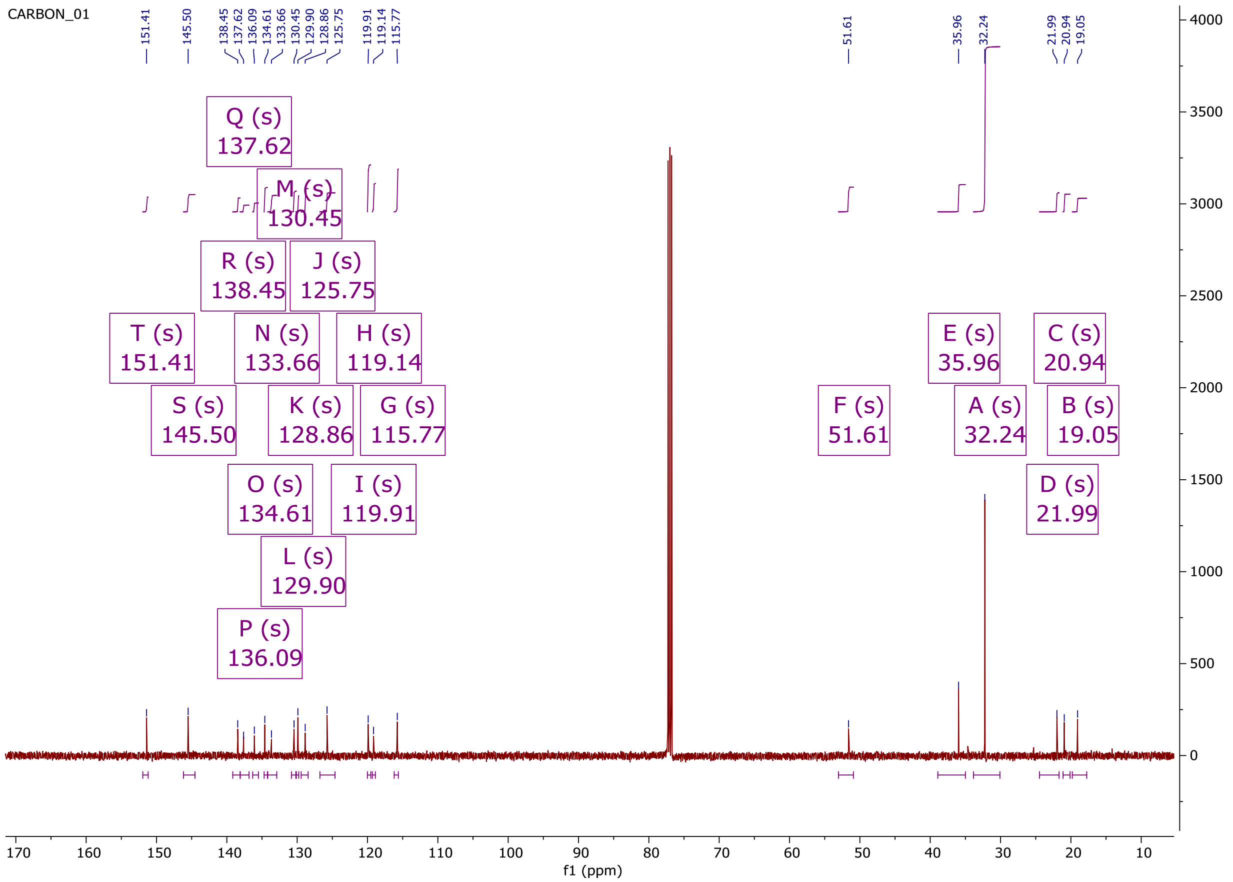

Figure S29. ^{13}C NMR (126 MHz) of compound **16-Mes** in $CDCl_3$.

Compound 17-Tips:

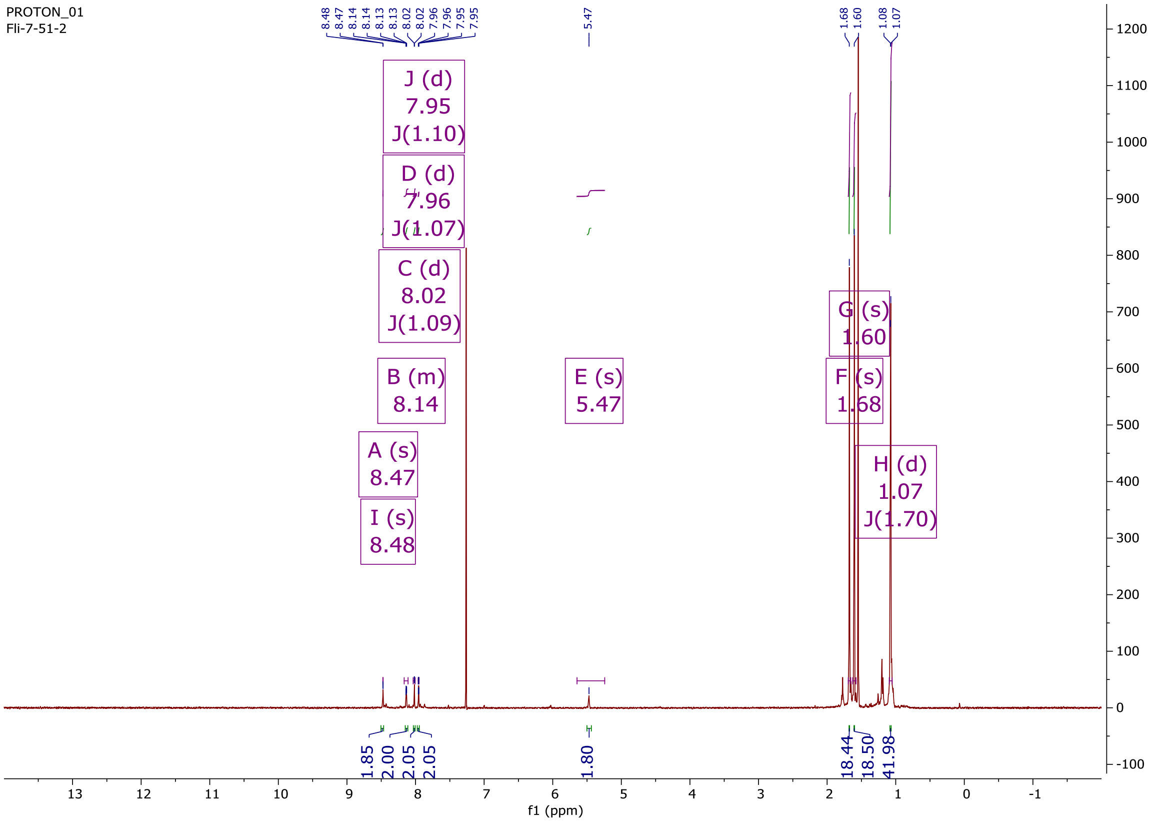

Figure S30. ^{1}H NMR (400 MHz) of compound **17-Tips** in $CDCl_3$.

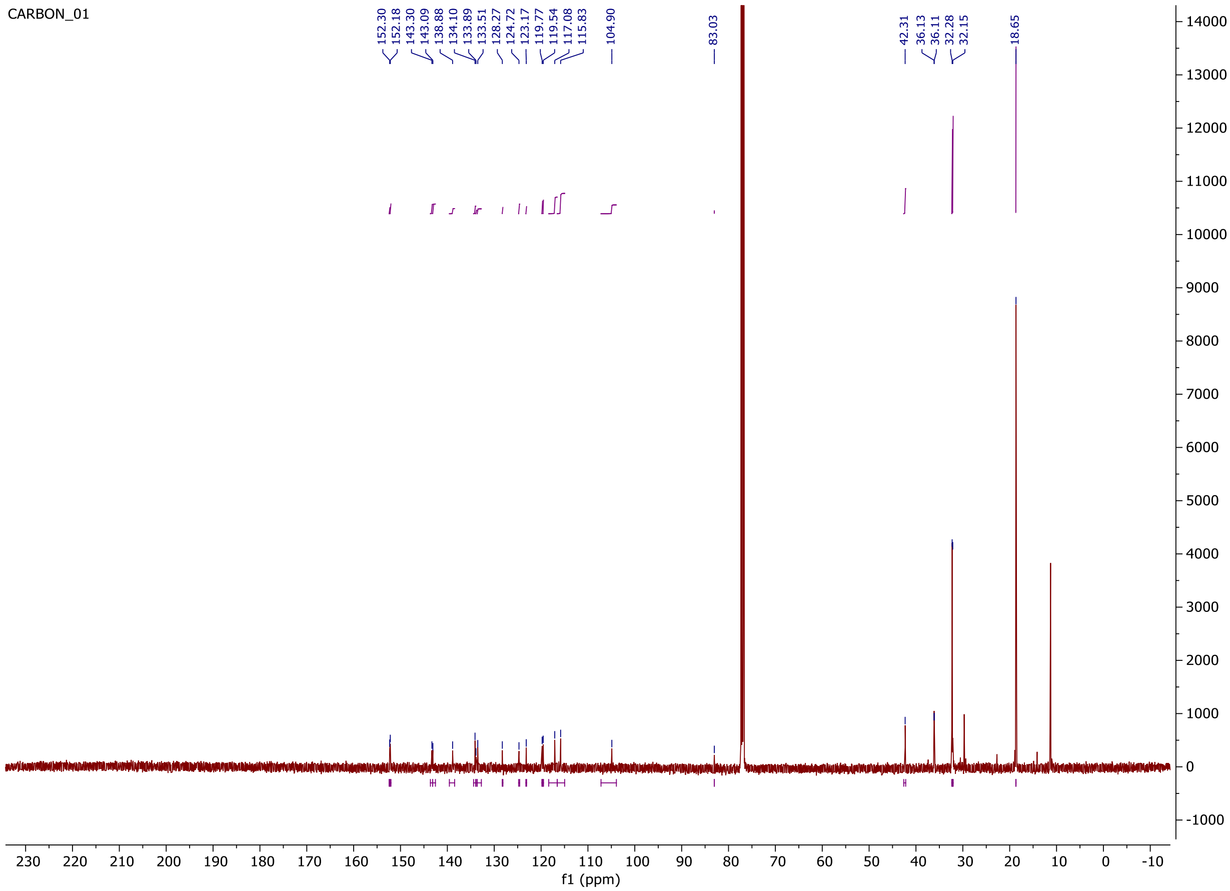

Figure S31. ^{13}C NMR (126 MHz) of compound **17-Tips** in $CDCl_3$.

Compound 17-Mes:

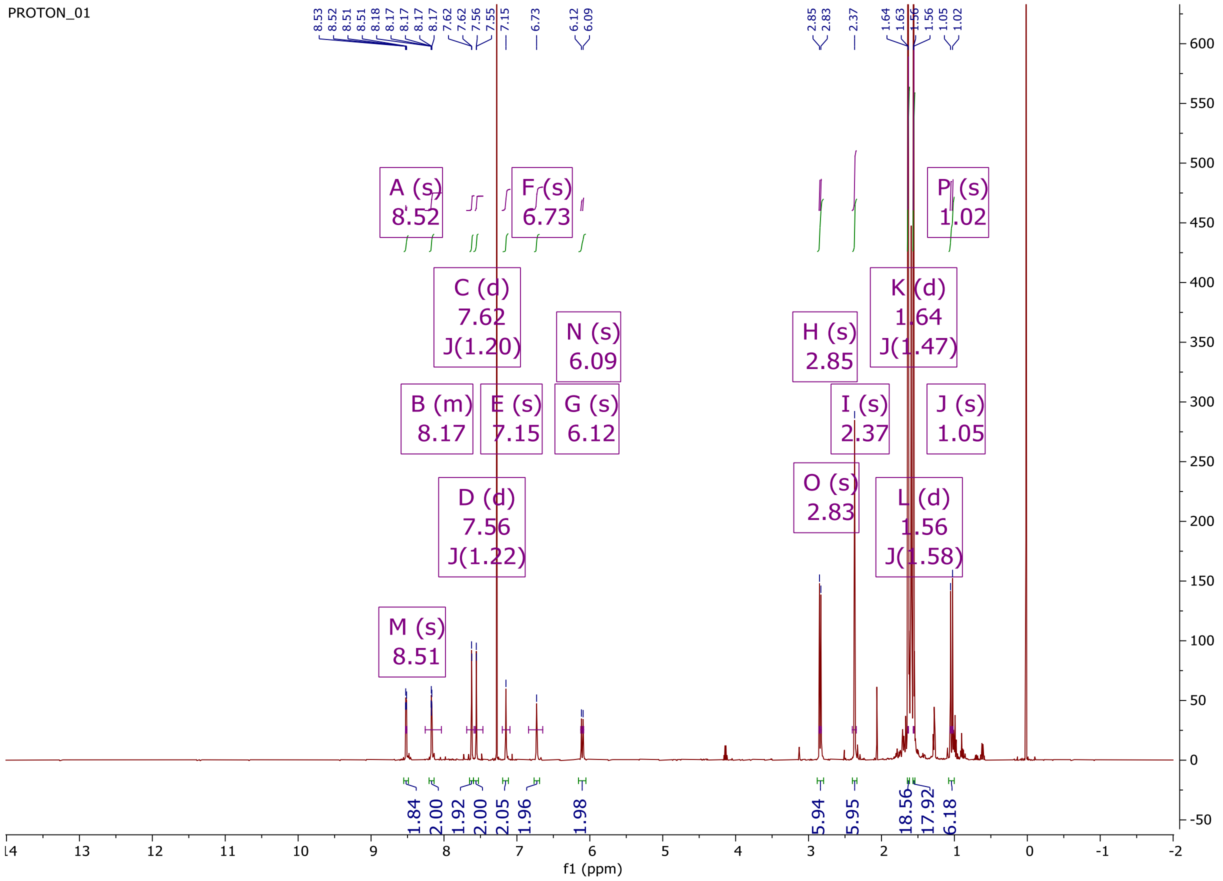

Figure S32. ^{1}H NMR (500 MHz) of compound **17-Mes** in $CDCl_3$.

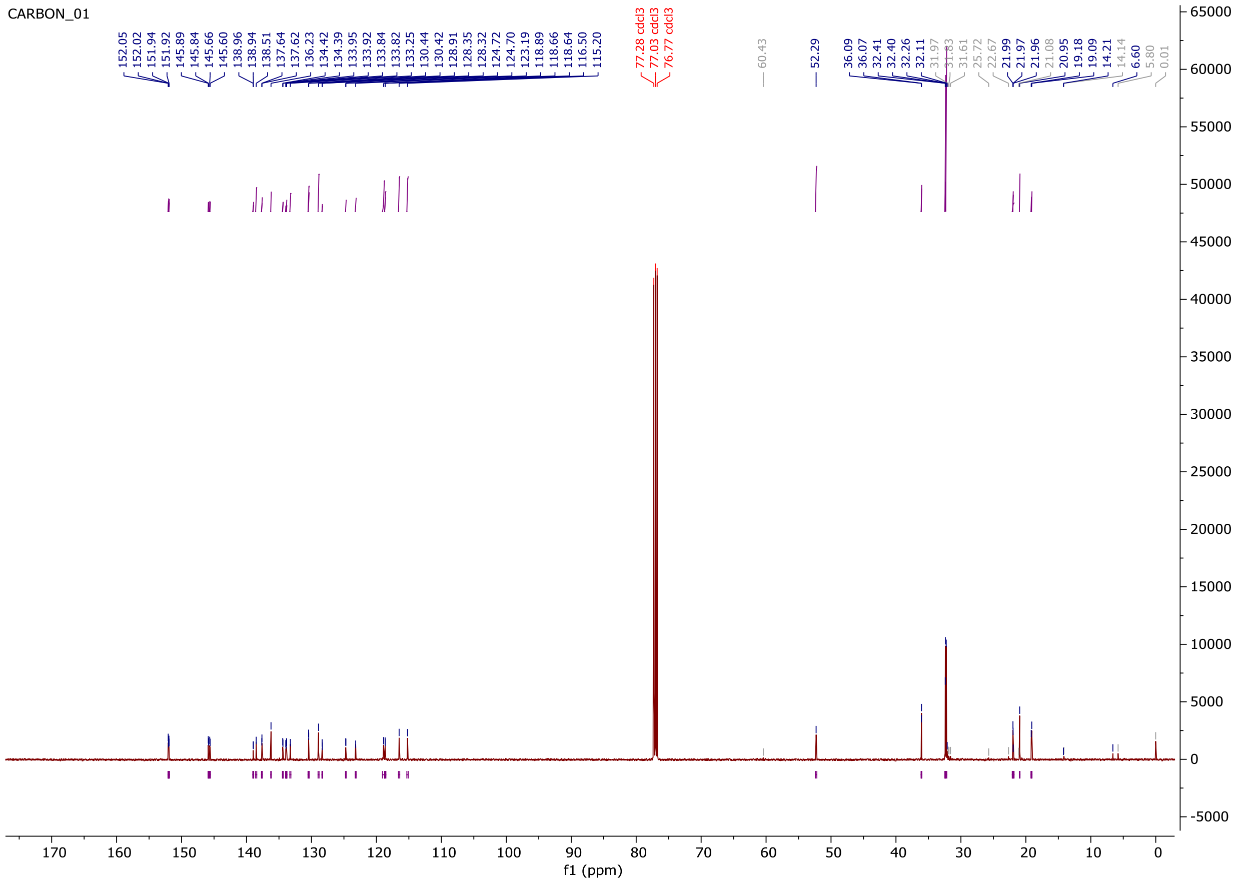

Figure S33. ^{13}C NMR (126 MHz) of compound **17-Mes** in $CDCl_3$.

UV-visible spectroscopy

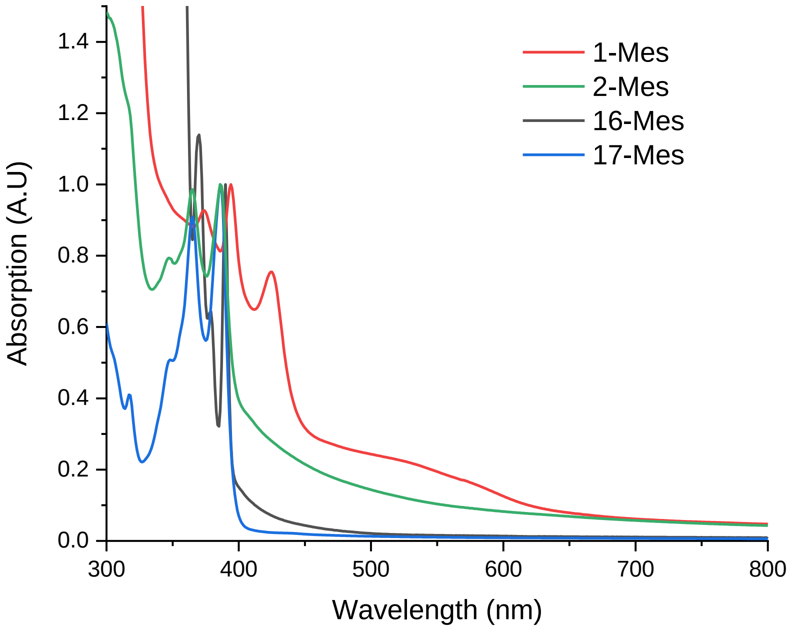

Figure S34. Normalized UV-visible absorption of **1-Mes** (red) and **2-Mes** (green) (both *in situ* generated) as well as **16-Mes** (black) and **17-Mes** (blue) in THF under a N_2 atmosphere at 25 °C

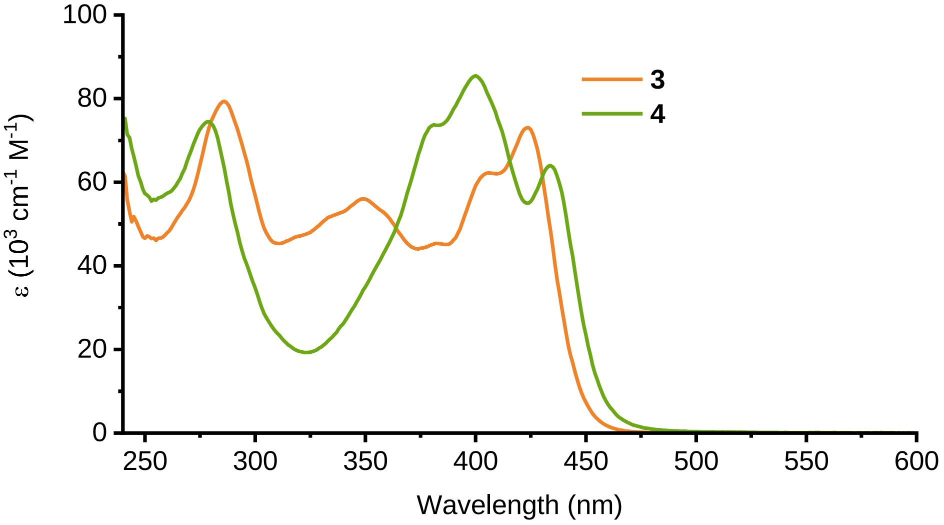

Figure S35. UV-visible absorption spectra of **3** and **4** in chloroform at 25 °C.

Chemical oxidation

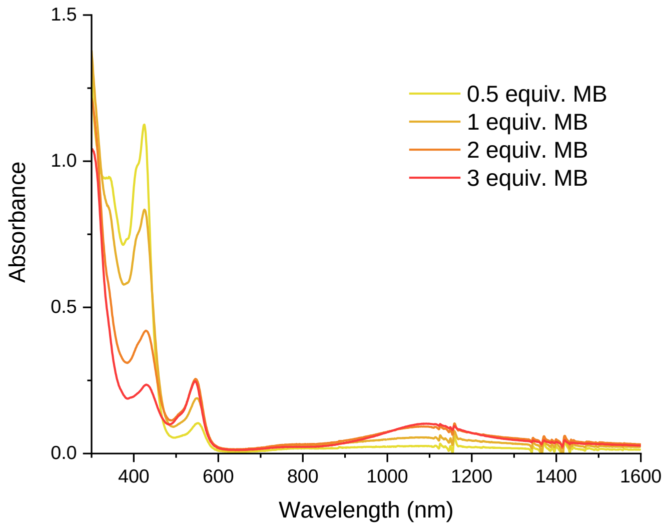

Figure S36. UV-Vis-NIR absorption spectra of chemically oxidized solutions of compound **3** by Magic Blue in CH_2Cl_2 at 25 °C.

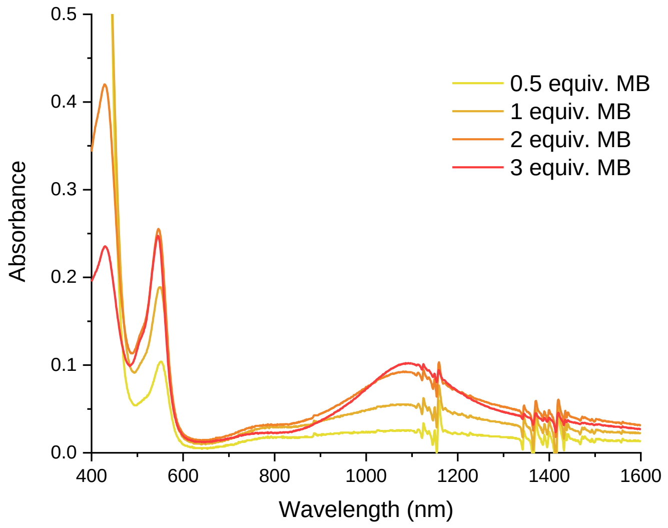

Figure S37. UV-Vis-NIR absorption spectra of chemically oxidized solutions of compound **3** by Magic Blue in CH_2Cl_2 at 25 °C.

Electrochemistry

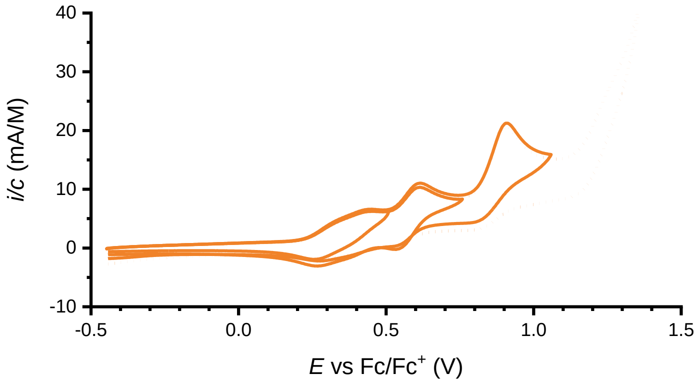

Figure S38. Cyclic voltammogram of **3**. Scan rate: 0.1 V/s. All potentials are corrected to the Fc/Fc$^+$ redox couple, measured at 0.5 mM of **3** in CH_2Cl_2 with Bu_4NPF_6 (0.1 M) as electrolyte.

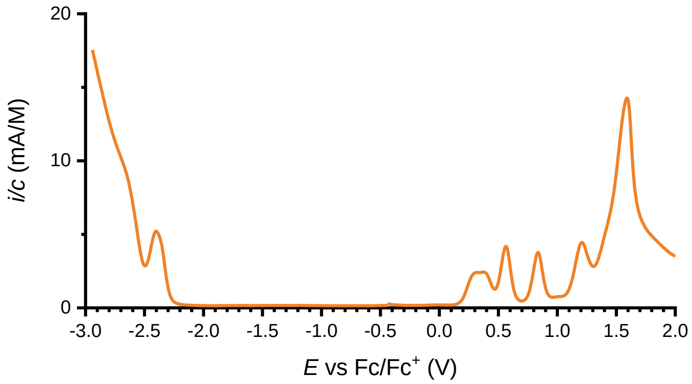

Figure S39. Differential pulse voltammogram of **3** (0.5 mM). Scan rate: 0.004 V/s. All potentials are corrected to the Fc/Fc$^+$ redox couple, measured at 0.5 mM of **3** in CH_2Cl_2 with Bu_4NPF_6 (0.1 M) as electrolyte.

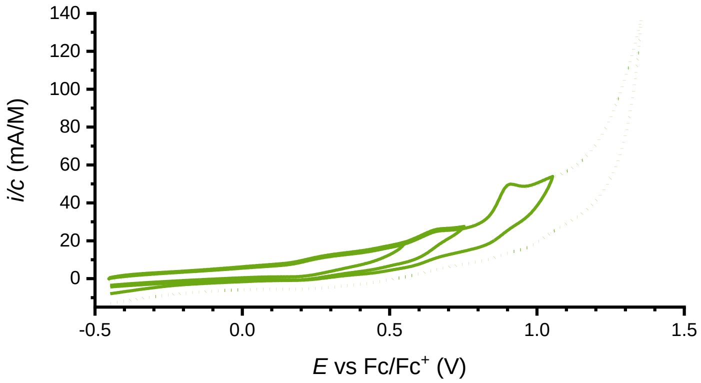

Figure S40. Cyclic voltammogram of **4**. Scan rate: 0.1 V/s. All potentials are corrected to the Fc/Fc^+ redox couple, measured at 0.5 mM of **4** in CH_2Cl_2 with Bu_4NPF_6 (0.1 M) as electrolyte.

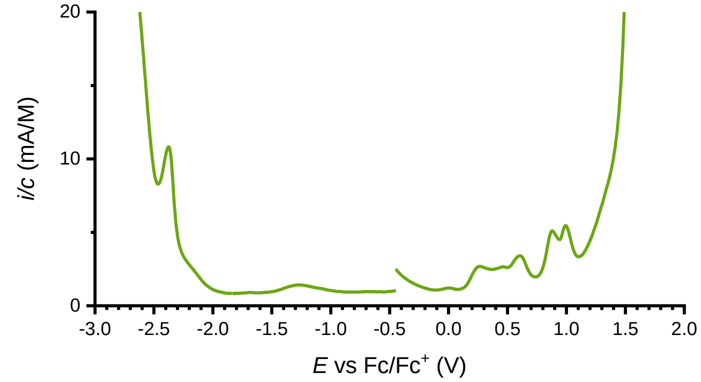

Figure S41. Differential pulse voltammogram of **4** (approximately 0.1 mM). Scan rate: 0.004 V/s. All potentials are corrected to the Fc/Fc^+ redox couple, measured at 0.5 mM of **4** in CH_2Cl_2 with Bu_4NPF_6 (0.1 M) as electrolyte.

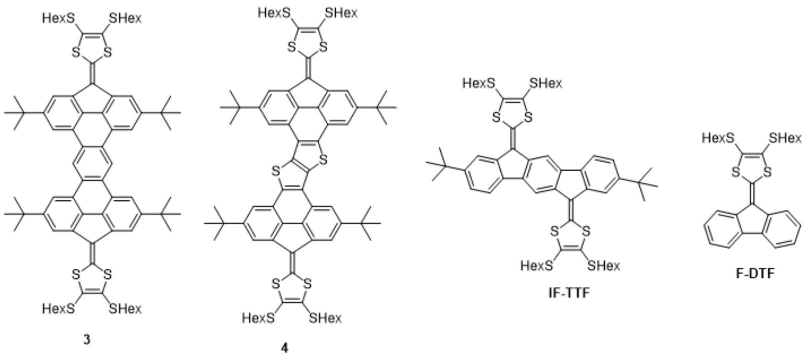

Figure S42. Molecular structure of different TTF-fluorene derivatives.

Table S1. Redox properties of compound **3** and **4** and other molecules for comparison.

Compound (core)	E^1_{ox} [V]	E^2_{ox} [V]	E^3_{ox} [V]	E^4_{ox} [V]	E^1_{red} [V]
**3**	0.32	0.38	0.56	0.84	-2.40
**4**	0.26	0.47	0.61	0.88	-2.38
IF-TTF[57]	0.27	0.39			-2.27
F-DTF[58]	0.43				-2.39
All potentials vs Fc/Fc$^+$		Irreversible processes shaded in grey			

EPR spectroscopy

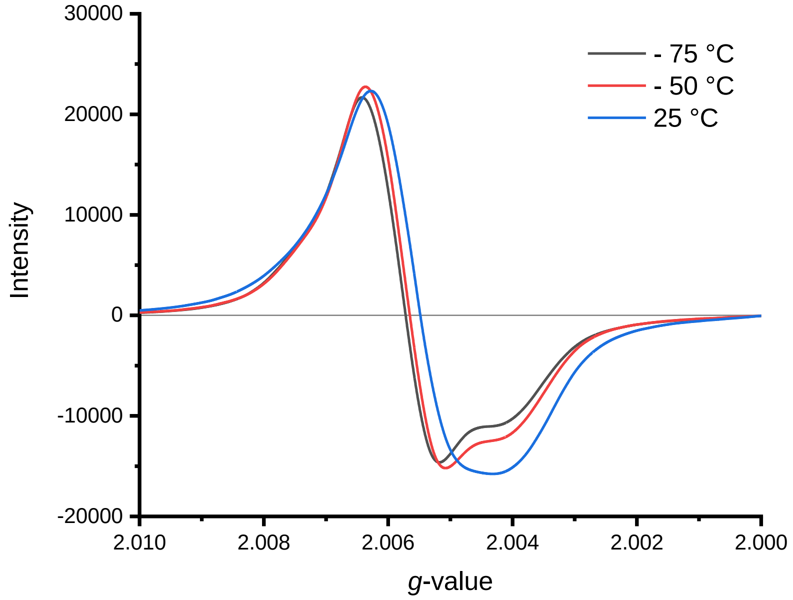

Figure S43. EPR spectra of *in-situ* generated compound **1-Tips** in THF at variable temperature.

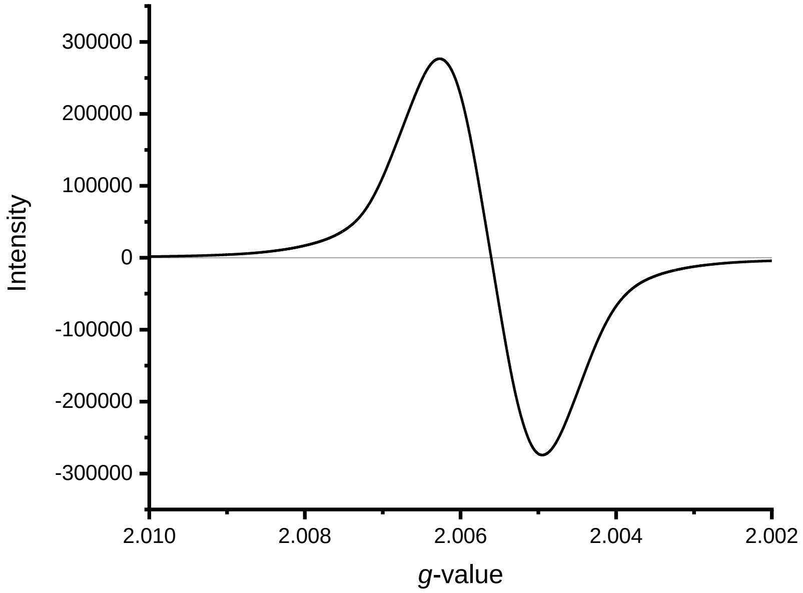

Figure S44. EPR spectra of *in-situ* generated compound **2-Tips** in THF at -75 °C.

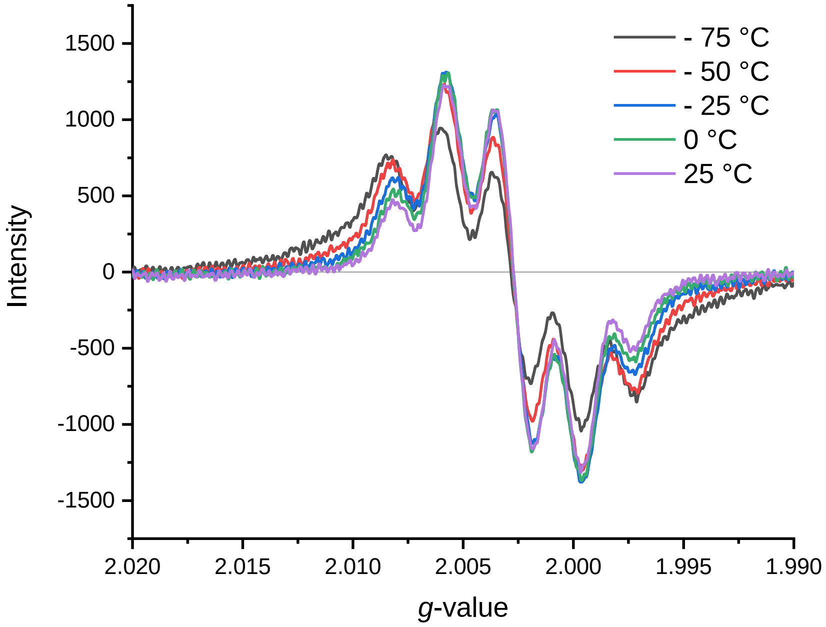

Figure S45. EPR spectra of *in-situ* generated compound **1-Mes** in THF at variable temperature.

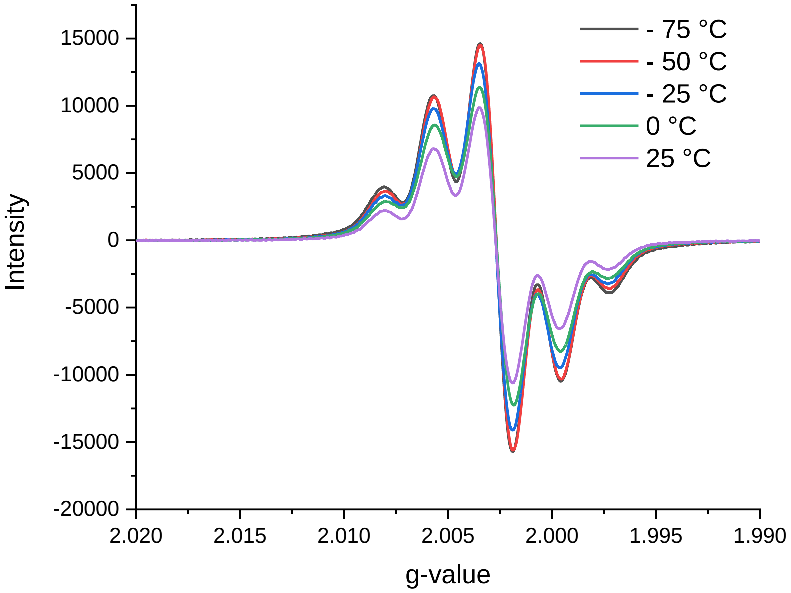

Figure S46. EPR spectra of *in-situ* generated compound **2-Mes** in THF at variable temperature.

Radical Quenching Experiment

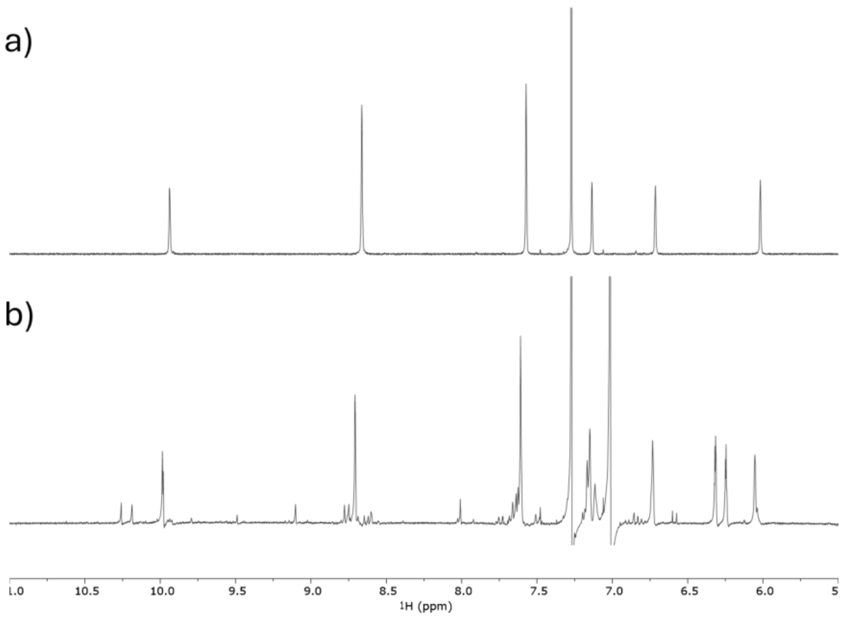

Figure S47. (a) Aromatic region of the ^{1}H NMR of **16-Mes** in $CDCl_3$ at 25 °C and (b) aromatic region of the ^{1}H NMR of **1-Mes** in $CDCl_3$ at 25 °C after treatment with tri-*n*-butyltin hydride (Bu_3SnH).

Computational Details

All the calculations reported in this paper were obtained with the GAUSSIAN 09 suite of programs.[59] Electron correlation was partially taken into account using the B3LYP[60–62] functional in conjunction with the D3 dispersion correction suggested by Grimme et al.[63] and the double-□ quality plus polarization functions def2-SVP[64] basis set for all atoms. All species were characterized by frequency calculations,[65] and have positive definite Hessian matrices. This level is denoted B3LYP-D3/def2-SVP, which was proven to provide good results for strongly related systems.[66,67] Calculations of the absorption spectrum were accomplished using time-dependent density functional theory (TD-DFT)[68,69] at the B3LYP-D3/def2-SVP level using the optimized geometries. The assignment of the excitation energies to the experimental bands was performed on

the basis of the energy values and oscillator strengths. The B3LYP Hamiltonian was chosen because it was proven to provide reasonable UV-vis spectra for a variety of chromophores.[70] For the TD-DFT calculations, solvent effects were taken into account by using the Polarizable Continuum Model (PCM).[71–73] The aromaticity of the considered species has been assessed by the computation of the NICS[74] values computed using the gauge invariant atomic orbital (GIAO) method[75] at the B3LYP/def2-SVP level using the optimized B3LYP-D·/def2-SVP geometries. Cartesian coordinates (in Å) and total energies (in a.u., ZPVE included) of all the stationary points discussed in the text. All calculations have been performed at the B3LYP-D3/def2-SVP level.

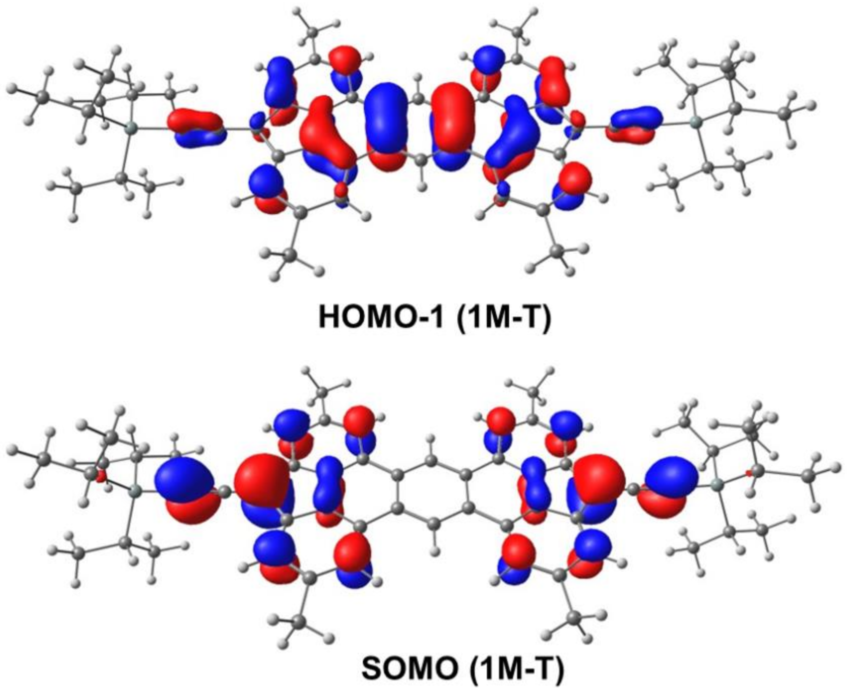

Figure S48. Frontier molecular orbitals for 1M-T

1M: E= -2825.045688

C	2.726618000	-2.751842000	0.026993000
C	4.030553000	-3.280446000	0.039168000
C	5.170469000	-2.432818000	0.057033000
C	4.987232000	-1.047661000	0.064196000
C	3.649624000	-0.574616000	0.050732000
C	2.500615000	-1.348451000	0.031984000
H	1.886244000	-3.449726000	0.012506000
H	6.172539000	-2.870967000	0.063796000
C	5.854009000	0.130198000	0.080194000

C	4.986932000	1.307560000	0.076080000
C	5.168247000	2.693003000	0.084035000
C	4.027598000	3.539693000	0.072472000
C	2.724261000	3.010047000	0.051840000
C	2.499842000	1.606496000	0.043030000
C	3.649424000	0.833550000	0.056991000
H	6.169846000	3.132023000	0.098521000
H	1.883225000	3.707206000	0.042982000
C	1.234712000	-0.597829000	0.017844000
C	0.000019000	-1.257497000	0.000633000
C	1.234567000	0.855154000	0.020755000
C	-1.234680000	-0.597843000	-0.016605000
C	0.000002000	1.514735000	0.000646000
C	-1.234554000	0.855139000	-0.019502000
H	0.000026000	-2.347852000	0.000639000
H	-0.000004000	2.605065000	0.000660000
C	-2.499837000	1.606465000	-0.041881000
C	-2.500573000	-1.348482000	-0.030844000
C	-3.649593000	-0.574660000	-0.049603000
C	-3.649409000	0.833505000	-0.055870000
C	-4.987187000	-1.047724000	-0.063571000
C	-5.170402000	-2.432886000	-0.056806000
H	-6.172459000	-2.871055000	-0.064158000
C	-2.726553000	-2.751876000	-0.026216000
H	-1.886170000	-3.449750000	-0.011835000
C	-2.724267000	3.010012000	-0.051019000
H	-1.883237000	3.707180000	-0.042236000
C	-4.986917000	1.307497000	-0.075454000
C	-5.853975000	0.130127000	-0.079896000
C	-5.168241000	2.692937000	-0.083768000
H	-6.169838000	3.131947000	-0.098805000
C	-4.027605000	3.539641000	-0.071998000
C	-4.030478000	-3.280498000	-0.038736000
C	-4.245326000	-4.777003000	-0.031378000
H	-4.816813000	-5.101262000	-0.917245000
H	-3.293568000	-5.328412000	-0.025214000
H	-4.822462000	-5.091541000	0.854433000
C	-4.240614000	5.036437000	-0.081263000
H	-4.808826000	5.351985000	-0.972390000
H	-4.819825000	5.361365000	0.799444000
H	-3.288131000	5.586621000	-0.078174000
C	4.240581000	5.036487000	0.082609000
H	4.799959000	5.352621000	0.979157000
H	4.828504000	5.360777000	-0.792486000
H	3.288204000	5.586712000	0.069897000
C	4.245416000	-4.776955000	0.032980000
H	4.805645000	-5.102001000	0.925793000
H	3.293788000	-5.328311000	0.014548000
H	4.833698000	-5.090754000	-0.845661000
C	-7.246808000	0.122870000	-0.089705000
C	-8.479345000	0.100367000	-0.095136000
C	7.246844000	0.122970000	0.089488000

C	8.479382000	0.100470000	0.094460000
Si	-10.323676000	0.034100000	-0.043966000
Si	10.323697000	0.034048000	0.043007000
C	-10.987566000	1.181085000	-1.430484000
H	-10.899576000	2.203607000	-1.015167000
C	-12.466833000	0.929781000	-1.773640000
C	-10.126086000	1.109919000	-2.703559000
H	-9.073195000	1.363102000	-2.507620000
H	-10.143735000	0.097295000	-3.143366000
H	-10.506411000	1.803046000	-3.474464000
H	-12.606589000	-0.066904000	-2.224635000
H	-12.825970000	1.668020000	-2.511889000
H	-13.129862000	0.993105000	-0.897573000
C	-10.846555000	-1.783769000	-0.367963000
H	-10.856962000	-1.858155000	-1.472661000
C	-12.265489000	-2.108175000	0.135159000
C	-9.836096000	-2.819376000	0.155625000
H	-8.826918000	-2.650165000	-0.249745000
H	-9.757591000	-2.787364000	1.255017000
H	-10.146757000	-3.842492000	-0.120406000
H	-12.313944000	-2.092042000	1.236011000
H	-12.569788000	-3.120355000	-0.184282000
H	-13.024826000	-1.405117000	-0.238662000
C	-10.787875000	0.709654000	1.687272000
H	-10.188876000	1.638733000	1.747681000
C	-10.310047000	-0.219675000	2.816323000
C	-12.264235000	1.100574000	1.863886000
H	-12.581673000	1.864038000	1.136660000
H	-12.937090000	0.235098000	1.754384000
H	-12.436612000	1.519058000	2.871267000
H	-10.881102000	-1.162634000	2.833301000
H	-10.445017000	0.257107000	3.803000000
H	-9.243968000	-0.477625000	2.713177000
C	10.787826000	0.710391000	-1.687938000
H	10.188997000	1.639620000	-1.747767000
C	10.309719000	-0.218282000	-2.817408000
C	12.264239000	1.101145000	-1.864545000
H	12.581928000	1.864193000	-1.136987000
H	12.936941000	0.235489000	-1.755569000
H	12.436540000	1.520101000	-2.871743000
H	10.880569000	-1.161362000	-2.834842000
H	10.444716000	0.258930000	-3.803870000
H	9.243595000	-0.476063000	-2.714291000
C	10.846373000	-1.784018000	0.366094000
H	10.857046000	-1.858971000	1.470745000
C	12.265137000	-2.108407000	-0.137527000
C	9.835614000	-2.819201000	-0.157764000
H	8.826574000	-2.650044000	0.247974000
H	9.756811000	-2.786602000	-1.257120000
H	10.146200000	-3.842506000	0.117647000
H	12.313341000	-2.091763000	-1.238383000
H	12.569359000	-3.120783000	0.181365000

H	13.024664000	-1.405638000	0.236457000
C	10.987879000	1.180416000	1.429892000
H	10.900088000	2.203081000	1.014886000
C	12.467096000	0.928616000	1.772879000
C	10.126454000	1.109061000	2.702997000
H	9.073613000	1.362543000	2.507186000
H	10.143889000	0.096293000	3.142483000
H	10.506972000	1.801867000	3.474098000
H	12.606585000	-0.068276000	2.223510000
H	12.826482000	1.666489000	2.511370000
H	13.130084000	0.992043000	0.896792000

1M(triplet): E= -2825.074639

C	2.718171000	-2.767420000	-0.039775000
C	4.018085000	-3.300672000	-0.029061000
C	5.162450000	-2.456191000	0.009190000
C	4.980363000	-1.074969000	0.035478000
C	3.648642000	-0.594678000	0.021602000
C	2.496950000	-1.364068000	-0.013966000
H	1.874926000	-3.461411000	-0.069217000
H	6.163417000	-2.896688000	0.017133000
C	5.855528000	0.106706000	0.076380000
C	4.985345000	1.292343000	0.084819000
C	5.171872000	2.672916000	0.116684000
C	4.030529000	3.522270000	0.112797000
C	2.728803000	2.994674000	0.077457000
C	2.502775000	1.592289000	0.044685000
C	3.651600000	0.817931000	0.050348000
H	6.174305000	3.109137000	0.144384000
H	1.888061000	3.692302000	0.076208000
C	1.232499000	-0.608243000	-0.019147000
C	-0.003935000	-1.264942000	-0.048011000
C	1.235548000	0.842110000	0.007652000
C	-1.237512000	-0.602293000	-0.053385000
C	0.002086000	1.504756000	-0.001232000
C	-1.234271000	0.848138000	-0.031003000
H	-0.006341000	-2.355131000	-0.065752000
H	0.004470000	2.594910000	0.017311000
C	-2.498316000	1.604557000	-0.036601000
C	-2.505309000	-1.352108000	-0.078351000
C	-3.653747000	-0.577059000	-0.080042000
C	-3.650596000	0.835720000	-0.060712000
C	-4.987692000	-1.050988000	-0.097318000
C	-5.175932000	-2.431563000	-0.114686000
H	-6.178824000	-2.867446000	-0.126158000
C	-2.732701000	-2.754587000	-0.097916000
H	-1.892427000	-3.452799000	-0.098478000
C	-2.718271000	3.008181000	-0.018835000
H	-1.874392000	3.701763000	-0.000231000
C	-4.982484000	1.316656000	-0.065798000
C	-5.857819000	0.135060000	-0.087949000

C	-5.163028000	2.698290000	-0.047345000
H	-6.163636000	3.139541000	-0.049587000
C	-4.017847000	3.542181000	-0.024577000
C	-4.035073000	-3.281625000	-0.115748000
C	-4.252504000	-4.777336000	-0.135707000
H	-4.818500000	-5.084846000	-1.031153000
H	-3.301892000	-5.330740000	-0.132422000
H	-4.837000000	-5.105803000	0.740182000
C	-4.227193000	5.039096000	-0.005889000
H	-4.791308000	5.372756000	-0.893127000
H	-4.809763000	5.348195000	0.878322000
H	-3.273558000	5.587006000	0.011591000
C	4.246296000	5.017999000	0.147230000
H	4.809125000	5.317970000	1.047198000
H	4.832827000	5.355310000	-0.723981000
H	3.295048000	5.570319000	0.146099000
C	4.228916000	-4.797175000	-0.058271000
H	4.788917000	-5.137170000	0.829148000
H	3.275936000	-5.345841000	-0.084950000
H	4.816409000	-5.098666000	-0.941848000
C	-7.248635000	0.130251000	-0.089480000
C	-8.481951000	0.108884000	-0.087207000
C	7.246092000	0.096344000	0.099858000
C	8.479244000	0.071491000	0.118584000
Si	-10.328569000	0.042918000	-0.024715000
Si	10.327196000	0.015618000	0.109936000
C	-10.996559000	1.186243000	-1.411091000
H	-10.904253000	2.210152000	-1.000179000
C	-12.478183000	0.937118000	-1.745874000
C	-10.141576000	1.108557000	-2.688267000
H	-9.087412000	1.361694000	-2.499215000
H	-10.162807000	0.094256000	-3.124073000
H	-10.524888000	1.799043000	-3.459985000
H	-12.622582000	-0.060852000	-2.192519000
H	-12.839355000	1.673546000	-2.484855000
H	-13.136487000	1.005145000	-0.866577000
C	-10.851891000	-1.775549000	-0.342518000
H	-10.868287000	-1.852327000	-1.446975000
C	-12.268222000	-2.098325000	0.169063000
C	-9.838940000	-2.810271000	0.178076000
H	-8.831695000	-2.642567000	-0.232646000
H	-9.754753000	-2.775988000	1.276954000
H	-10.151428000	-3.833814000	-0.094112000
H	-12.310250000	-2.080762000	1.270155000
H	-12.574984000	-3.110679000	-0.147340000
H	-13.029196000	-1.395164000	-0.201237000
C	-10.779330000	0.720686000	1.707917000
H	-10.180438000	1.650226000	1.763086000
C	-10.293427000	-0.206844000	2.835184000
C	-12.254589000	1.111404000	1.894536000
H	-12.577570000	1.873095000	1.167894000
H	-12.927693000	0.245325000	1.791619000

H	-12.419930000	1.531918000	2.902178000
H	-10.864414000	-1.149717000	2.857670000
H	-10.421421000	0.271645000	3.821917000
H	-9.228057000	-0.465203000	2.725634000
C	10.849099000	1.283883000	-1.223655000
H	10.247762000	2.173606000	-0.953686000
C	10.420507000	0.848660000	-2.635414000
C	12.329091000	1.699378000	-1.186407000
H	12.616728000	2.127643000	-0.213374000
H	13.001215000	0.850152000	-1.389131000
H	12.536411000	2.466200000	-1.953542000
H	10.995275000	-0.027154000	-2.978749000
H	10.594733000	1.656782000	-3.367264000
H	9.351990000	0.583821000	-2.678522000
C	10.842398000	-1.798085000	-0.245873000
H	10.796909000	-2.279499000	0.750445000
C	12.286335000	-1.933220000	-0.764553000
C	9.866071000	-2.550293000	-1.167331000
H	8.833963000	-2.524988000	-0.786097000
H	9.850687000	-2.115712000	-2.180525000
H	10.164863000	-3.608361000	-1.271023000
H	12.389651000	-1.509616000	-1.776916000
H	12.577318000	-2.996214000	-0.830966000
H	13.023557000	-1.431615000	-0.120116000
C	10.930656000	0.554510000	1.849518000
H	10.859339000	1.659249000	1.842725000
C	12.394090000	0.170759000	2.131906000
C	10.016899000	0.026539000	2.969466000
H	8.974531000	0.357039000	2.846483000
H	10.011123000	-1.077318000	2.995403000
H	10.368960000	0.370443000	3.957928000
H	12.513622000	-0.924123000	2.187192000
H	12.722530000	0.578069000	3.104097000
H	13.093723000	0.541996000	1.367836000

2M: E= -3462.965983

C	-3.189398000	2.503273000	-0.035124000
C	-4.468338000	3.082194000	-0.063933000
C	-5.664469000	2.330892000	-0.029456000
C	-5.566719000	0.935099000	0.038594000
C	-4.256013000	0.383889000	0.068388000
C	-3.053606000	1.091193000	0.033110000
H	-2.312523000	3.153035000	-0.066833000
H	-4.540529000	4.171519000	-0.116608000
H	-6.633947000	2.834166000	-0.056057000
C	-6.500514000	-0.184942000	0.084841000
C	-5.703116000	-1.411609000	0.145369000
C	-5.963496000	-2.783166000	0.202633000
C	-4.861642000	-3.675997000	0.242461000
C	-3.528292000	-3.253375000	0.225637000
C	-3.230444000	-1.861571000	0.166915000

C	-4.339090000	-1.015859000	0.132099000
H	-6.985388000	-3.169876000	0.216247000
H	-5.065415000	-4.748828000	0.287536000
H	-2.724932000	-3.993701000	0.256538000
C	-1.862629000	0.255137000	0.067087000
S	0.337047000	2.175480000	-0.040253000
C	-1.969035000	-1.163508000	0.131290000
C	1.902654000	1.380754000	-0.023751000
C	0.418360000	-0.420184000	0.077693000
C	1.794800000	-0.037560000	0.042646000
S	-0.403437000	-1.957138000	0.154316000
C	2.984151000	-0.875970000	0.061408000
C	3.165135000	2.076402000	-0.075628000
C	4.272757000	1.228531000	-0.057358000
C	4.187068000	-0.170971000	0.007580000
C	5.637795000	1.620707000	-0.101094000
C	5.899248000	2.991868000	-0.167273000
H	6.921000000	3.377393000	-0.204093000
C	3.464821000	3.467352000	-0.143220000
H	2.662671000	4.209402000	-0.161609000
C	3.117924000	-2.288526000	0.123440000
H	2.240379000	-2.936474000	0.169447000
C	5.496100000	-0.725608000	0.007332000
C	6.432213000	0.391506000	-0.062398000
C	5.592506000	-2.121624000	0.069084000
H	6.561508000	-2.626535000	0.073584000
C	4.395773000	-2.870490000	0.126278000
H	4.466489000	-3.960099000	0.174780000
C	4.798605000	3.886869000	-0.186236000
H	5.003836000	4.959135000	-0.237601000
C	-0.486030000	0.638942000	0.039068000
C	7.820894000	0.290088000	-0.090546000
C	9.047651000	0.171245000	-0.116944000
C	-7.890579000	-0.106737000	0.058904000
C	-9.120947000	-0.038260000	0.026028000
Si	10.868225000	-0.125525000	-0.137434000
Si	-10.960318000	0.072539000	-0.073838000
C	11.198330000	-1.622719000	1.004661000
H	12.284510000	-1.815984000	0.917853000
C	10.448297000	-2.879877000	0.532117000
C	10.880534000	-1.323777000	2.479142000
H	11.490236000	-0.500134000	2.880167000
H	9.820652000	-1.048500000	2.611978000
H	11.071423000	-2.210110000	3.109500000
H	9.357322000	-2.716186000	0.541437000
H	10.658876000	-3.736419000	1.196173000
H	10.728248000	-3.179458000	-0.489172000
C	11.743097000	1.473215000	0.453627000
H	11.732026000	2.117628000	-0.446633000
C	13.214704000	1.233711000	0.836650000
C	10.997775000	2.231755000	1.565372000
H	9.952087000	2.440849000	1.291679000

H	10.980946000	1.663958000	2.509404000
H	11.492545000	3.196140000	1.776513000
H	13.302716000	0.571639000	1.714485000
H	13.711896000	2.184080000	1.098188000
H	13.796075000	0.777090000	0.020284000
C	11.403456000	-0.495509000	-1.947086000
H	11.582958000	0.508462000	-2.377070000
C	10.313631000	-1.171225000	-2.797033000
C	12.726109000	-1.279568000	-2.025254000
H	13.545966000	-0.790462000	-1.476222000
H	12.618682000	-2.297781000	-1.615890000
H	13.054525000	-1.387067000	-3.073823000
H	10.068661000	-2.178214000	-2.421496000
H	10.651103000	-1.287600000	-3.841986000
H	9.378376000	-0.591107000	-2.805185000
C	-11.692655000	-0.243638000	1.675455000
H	-12.715413000	-0.616202000	1.467897000
C	-11.825402000	1.013184000	2.551109000
C	-10.928162000	-1.338328000	2.441587000
H	-10.851850000	-2.280743000	1.880580000
H	-9.900665000	-1.009744000	2.668366000
H	-11.425864000	-1.563644000	3.401071000
H	-10.836448000	1.432371000	2.801094000
H	-12.321085000	0.767224000	3.506528000
H	-12.415967000	1.808347000	2.070432000
C	-11.415477000	1.832821000	-0.694261000
H	-12.446219000	1.990442000	-0.320092000
C	-11.449045000	1.981490000	-2.224280000
C	-10.512284000	2.919780000	-0.083259000
H	-10.494813000	2.896680000	1.015864000
H	-9.472313000	2.799182000	-0.429249000
H	-10.850116000	3.925543000	-0.388555000
H	-10.447405000	1.832650000	-2.660894000
H	-11.776638000	2.997186000	-2.507272000
H	-12.135545000	1.269435000	-2.707639000
C	-11.572933000	-1.268107000	-1.308354000
H	-12.547697000	-0.876031000	-1.660507000
C	-11.828953000	-2.648523000	-0.681591000
C	-10.643691000	-1.413445000	-2.527155000
H	-10.471595000	-0.463545000	-3.052639000
H	-9.657125000	-1.798628000	-2.221277000
H	-11.067093000	-2.124571000	-3.258102000
H	-10.892736000	-3.100243000	-0.312675000
H	-12.245051000	-3.342177000	-1.433123000
H	-12.538365000	-2.612511000	0.159252000

2M(triplet): E= -3462.995256

C	3.188554000	2.497978000	0.025376000
C	4.463553000	3.077126000	0.054538000
C	5.662795000	2.323779000	0.020775000
C	5.561800000	0.933312000	-0.046749000

C	4.256979000	0.379262000	-0.077046000
C	3.054561000	1.084486000	-0.042501000
H	2.310035000	3.145504000	0.056304000
H	4.536089000	4.166479000	0.106848000
H	6.632493000	2.826501000	0.047758000
C	6.502196000	-0.195427000	-0.092391000
C	5.700741000	-1.426419000	-0.153723000
C	5.962940000	-2.795847000	-0.210738000
C	4.859321000	-3.685870000	-0.250883000
C	3.526928000	-3.260208000	-0.234431000
C	3.230951000	-1.870022000	-0.175912000
C	4.341084000	-1.026102000	-0.140747000
H	6.984722000	-3.182345000	-0.223895000
H	5.060128000	-4.759363000	-0.295870000
H	2.722157000	-3.999059000	-0.265474000
C	1.863621000	0.248476000	-0.076526000
S	-0.335903000	2.172811000	0.031940000
C	1.968841000	-1.167282000	-0.140494000
C	-1.902095000	1.380014000	0.016114000
C	-0.418452000	-0.421690000	-0.086558000
C	-1.795584000	-0.035431000	-0.050656000
S	0.402639000	-1.959091000	-0.163359000
C	-2.985077000	-0.873587000	-0.068881000
C	-3.165147000	2.080386000	0.069497000
C	-4.274365000	1.234476000	0.052113000
C	-4.187899000	-0.170623000	-0.013849000
C	-5.634856000	1.631414000	0.097698000
C	-5.898007000	3.000374000	0.164707000
H	-6.919588000	3.385694000	0.202871000
C	-3.462782000	3.469763000	0.137844000
H	-2.659099000	4.210234000	0.155609000
C	-3.117354000	-2.287507000	-0.131410000
H	-2.238281000	-2.933264000	-0.178348000
C	-5.491128000	-0.727943000	-0.012834000
C	-6.433595000	0.397959000	0.059281000
C	-5.591050000	-2.118587000	-0.074636000
H	-6.560328000	-2.622871000	-0.078246000
C	-4.391327000	-2.869564000	-0.133375000
H	-4.462547000	-3.959157000	-0.182160000
C	-4.795465000	3.892488000	0.182631000
H	-4.997536000	4.965418000	0.234626000
C	0.486302000	0.635810000	-0.048127000
C	-7.819986000	0.295427000	0.090027000
C	-9.047199000	0.174191000	0.118936000
C	7.889975000	-0.115235000	-0.063101000
C	9.120718000	-0.042462000	-0.026092000
Si	-10.870087000	-0.125935000	0.144802000
Si	10.961835000	0.078011000	0.083276000
C	-11.197241000	-1.627145000	-0.991670000
H	-12.283560000	-1.820285000	-0.906291000
C	-10.448174000	-2.882913000	-0.513713000
C	-10.876986000	-1.333090000	-2.466709000

H	-11.485890000	-0.510742000	-2.871521000
H	-9.816796000	-1.058712000	-2.599149000
H	-11.066944000	-2.221528000	-3.094293000
H	-9.357122000	-2.719569000	-0.521020000
H	-10.657325000	-3.741313000	-1.175731000
H	-10.730616000	-3.179415000	0.507750000
C	-11.747176000	1.468677000	-0.452935000
H	-11.739859000	2.115966000	0.445268000
C	-13.217417000	1.224020000	-0.838342000
C	-11.001625000	2.225916000	-1.565508000
H	-9.957592000	2.440584000	-1.289815000
H	-10.980093000	1.654876000	-2.507494000
H	-11.499589000	3.187542000	-1.781390000
H	-13.301809000	0.560618000	-1.715490000
H	-13.716896000	2.172561000	-1.101956000
H	-13.798928000	0.766663000	-0.022475000
C	-11.395140000	-0.487249000	1.957522000
H	-11.569504000	0.518389000	2.385767000
C	-10.302812000	-1.163397000	2.804222000
C	-12.719908000	-1.267060000	2.045227000
H	-13.541406000	-0.775887000	1.500464000
H	-12.618344000	-2.286155000	1.636532000
H	-13.042289000	-1.371906000	3.095845000
H	-10.062678000	-2.172457000	2.431263000
H	-10.635348000	-1.274920000	3.851230000
H	-9.365815000	-0.586005000	2.806443000
C	11.701291000	-0.245173000	-1.660130000
H	12.725457000	-0.610646000	-1.447157000
C	11.830492000	1.006171000	-2.544359000
C	10.945977000	-1.349897000	-2.421261000
H	10.873149000	-2.288688000	-1.853722000
H	9.917451000	-1.029305000	-2.654836000
H	11.448582000	-1.579180000	-3.377186000
H	10.840327000	1.418484000	-2.800890000
H	12.330868000	0.756117000	-3.496203000
H	12.415173000	1.807742000	-2.067112000
C	11.401851000	1.844065000	0.697076000
H	12.435508000	2.003911000	0.331944000
C	11.419717000	2.001262000	2.226573000
C	10.500085000	2.923902000	0.071436000
H	10.493081000	2.894786000	-1.027618000
H	9.457374000	2.801404000	0.408399000
H	10.831086000	3.932616000	0.374326000
H	10.414393000	1.850965000	2.654163000
H	11.740363000	3.019824000	2.507067000
H	12.104165000	1.294719000	2.720745000
C	11.570718000	-1.250500000	1.331409000
H	12.537379000	-0.847832000	1.693775000
C	11.847250000	-2.631892000	0.715369000
C	10.628107000	-1.397525000	2.539812000
H	10.439582000	-0.445896000	3.056605000
H	9.649542000	-1.795669000	2.224904000

H	11.050067000	-2.099044000	3.280710000
H	10.919739000	-3.095375000	0.339261000
H	12.262485000	-3.316837000	1.475242000
H	12.565149000	-2.593279000	-0.118150000

1M': E= -2082.121680

C	-2.727288000	-2.883179000	0.011644000
C	-4.033377000	-3.408824000	0.005483000
C	-5.171804000	-2.560781000	0.003969000
C	-4.994168000	-1.170145000	0.019252000
C	-3.648499000	-0.705130000	0.009833000
C	-2.501110000	-1.478952000	0.008843000
H	-1.887802000	-3.582219000	0.013353000
H	-6.172113000	-3.002964000	-0.015346000
C	-5.856780000	-0.001853000	-0.002129000
C	-4.993908000	1.166216000	-0.023067000
C	-5.171662000	2.556810000	-0.009179000
C	-4.033226000	3.404833000	-0.010266000
C	-2.727155000	2.879119000	-0.014769000
C	-2.501012000	1.474878000	-0.010827000
C	-3.648385000	0.701086000	-0.012190000
H	-6.172079000	2.998815000	0.008387000
H	-1.887639000	3.578124000	-0.016338000
C	-1.234693000	-0.728426000	0.001413000
C	0.000026000	-1.388257000	0.000030000
C	-1.234684000	0.724306000	-0.002310000
C	1.234720000	-0.728378000	-0.001358000
C	-0.000027000	1.384176000	0.000025000
C	1.234657000	0.724354000	0.002359000
H	0.000048000	-2.478672000	0.000023000
H	-0.000049000	2.474587000	0.000016000
C	2.500957000	1.474976000	0.010854000
C	2.501164000	-1.478853000	-0.008811000
C	3.648523000	-0.704987000	-0.009871000
C	3.648361000	0.701229000	0.012153000
C	4.994206000	-1.169951000	-0.019303000
C	5.171902000	-2.560573000	-0.003969000
H	6.172231000	-3.002710000	0.015338000
C	2.727400000	-2.883073000	-0.011557000
H	1.887941000	-3.582146000	-0.013203000
C	2.727039000	2.879224000	0.014834000
H	1.887494000	3.578194000	0.016458000
C	4.993871000	1.166412000	0.023018000
C	5.856780000	-0.001617000	0.002072000
C	5.171559000	2.557020000	0.009153000
H	6.171953000	2.999073000	-0.008448000
C	4.033091000	3.404993000	0.010303000
C	4.033507000	-3.408664000	-0.005410000
C	4.249531000	-4.905449000	0.000785000

H	4.834692000	-5.219605000	0.881400000
H	3.298134000	-5.457388000	0.015700000
H	4.812715000	-5.230795000	-0.890193000
C	4.248938000	4.901808000	0.005719000
H	4.811818000	5.226285000	0.897203000
H	4.834330000	5.216953000	-0.874392000
H	3.297483000	5.453658000	-0.008913000
C	-4.249137000	4.901638000	-0.005542000
H	-4.812782000	5.226074000	-0.896552000
H	-4.833804000	5.216773000	0.875059000
H	-3.297696000	5.453532000	0.008296000
C	-4.249336000	-4.905619000	-0.000545000
H	-4.835194000	-5.219808000	-0.880679000
H	-3.297925000	-5.457513000	-0.016211000
H	-4.811781000	-5.230987000	0.890896000
C	7.327368000	0.000099000	0.002959000
C	8.040038000	0.509629000	1.117988000
C	8.043697000	-0.509125000	-1.113236000
C	9.440740000	0.497978000	1.096180000
C	9.441482000	-0.492945000	-1.090174000
C	10.162795000	0.005347000	0.003670000
H	9.984404000	0.875623000	1.967605000
H	9.988211000	-0.871203000	-1.959884000
C	-7.327371000	-0.000061000	-0.003054000
C	-8.039976000	0.509494000	-1.118095000
C	-8.043762000	-0.509187000	1.113160000
C	-9.440690000	0.498019000	-1.096283000
C	-9.441535000	-0.492848000	1.090099000
C	-10.162796000	0.005532000	-0.003754000
H	-9.984300000	0.875683000	-1.967733000
H	-9.988315000	-0.871049000	1.959801000
C	7.319070000	-1.016136000	-2.338531000
H	8.011665000	-1.144692000	-3.183350000
H	6.829922000	-1.985807000	-2.155102000
H	6.521385000	-0.321967000	-2.646108000
C	7.315198000	1.012270000	2.345023000
H	6.513049000	0.320772000	2.646717000
H	8.006709000	1.131925000	3.192056000
H	6.832110000	1.986038000	2.166746000
C	11.671368000	0.020066000	-0.016571000
H	12.090579000	0.287303000	0.964971000
H	12.078472000	-0.962959000	-0.305002000
H	12.049935000	0.751285000	-0.751745000
C	-7.315095000	1.012003000	-2.345164000
H	-6.512900000	0.320508000	-2.646734000
H	-8.006572000	1.131519000	-3.192245000
H	-6.832070000	1.985822000	-2.166985000
C	-7.319157000	-1.016208000	2.338461000
H	-8.011829000	-1.145159000	3.183155000
H	-6.829615000	-1.985652000	2.154925000
H	-6.521771000	-0.321797000	2.646288000

C	-11.671365000	0.020554000	0.016532000
H	-12.090564000	0.286711000	-0.965312000
H	-12.078662000	-0.962027000	0.306174000
H	-12.049746000	0.752764000	0.750821000

1M' (triplet): E= -2082.152912

C	-2.728475000	2.874903000	0.201287000
C	-4.031836000	3.399570000	0.243383000
C	-5.172963000	2.552632000	0.182803000
C	-4.990711000	1.171176000	0.074097000
C	-3.650130000	0.703460000	0.045533000
C	-2.502210000	1.474373000	0.101366000
H	-1.888142000	3.571301000	0.250453000
H	-6.174465000	2.990681000	0.224427000
C	-5.857837000	-0.001593000	0.000037000
C	-4.989795000	-1.173646000	-0.073986000
C	-5.170984000	-2.555169000	-0.183685000
C	-4.029207000	-3.401214000	-0.244353000
C	-2.726256000	-2.875591000	-0.201485000
C	-2.501088000	-1.474942000	-0.100730000
C	-3.649591000	-0.704933000	-0.044731000
H	-6.172143000	-2.993911000	-0.226236000
H	-1.885381000	-3.571317000	-0.250879000
C	-1.235367000	0.723923000	0.051049000
C	-0.000484000	1.382554000	0.097128000
C	-1.234845000	-0.723542000	-0.050018000
C	1.234872000	0.724786000	0.051412000
C	0.000492000	-1.381304000	-0.095736000
C	1.235357000	-0.722681000	-0.049641000
H	-0.000877000	2.470397000	0.171797000
H	0.000887000	-2.469142000	-0.170441000
C	2.502131000	-1.473202000	-0.099915000
C	2.501182000	1.476120000	0.102127000
C	3.649662000	0.706059000	0.046015000
C	3.650072000	-0.702336000	-0.044217000
C	4.989941000	1.174666000	0.075346000
C	5.171154000	2.556182000	0.185187000
H	6.172301000	2.994949000	0.227798000
C	2.726429000	2.876742000	0.203029000
H	1.885593000	3.572511000	0.252503000
C	2.728321000	-2.873761000	-0.199644000
H	1.887952000	-3.570122000	-0.248712000
C	4.990582000	-1.170154000	-0.072699000
C	5.857845000	0.002464000	0.001370000
C	5.172812000	-2.551621000	-0.181211000
H	6.174329000	-2.989636000	-0.222790000
C	4.031654000	-3.398513000	-0.241640000
C	4.029411000	3.402278000	0.245960000
C	4.244674000	4.894304000	0.362009000

H	4.833044000	5.279562000	-0.487749000
H	3.293246000	5.445683000	0.389318000
H	4.805375000	5.144967000	1.278380000
C	4.247919000	-4.890590000	-0.355157000
H	4.816416000	-5.141768000	-1.266527000
H	4.829050000	-5.275335000	0.499849000
H	3.296769000	-5.442015000	-0.390084000
C	-4.244401000	-4.893307000	-0.359665000
H	-4.828878000	-5.278852000	0.492671000
H	-4.809056000	-5.144101000	-1.273544000
H	-3.292893000	-5.444322000	-0.391132000
C	-4.248167000	4.891555000	0.357985000
H	-4.810686000	5.142566000	1.273138000
H	-3.297106000	5.443499000	0.386608000
H	-4.835195000	5.275862000	-0.493143000
C	7.332815000	0.001643000	0.001874000
C	8.043701000	0.517953000	-1.110208000
C	8.041307000	-0.513462000	1.112670000
C	9.442094000	0.505411000	-1.088193000
C	9.442531000	-0.502624000	1.091582000
C	10.163051000	-0.000834000	0.002187000
H	9.987872000	0.896060000	-1.952945000
H	9.986204000	-0.891116000	1.958161000
C	-7.332803000	-0.002188000	-0.000021000
C	-8.042333000	-0.519944000	1.109664000
C	-8.042689000	0.511405000	-1.113178000
C	-9.442856000	-0.511702000	1.086376000
C	-9.441786000	0.496072000	-1.093497000
C	-10.163182000	-0.007807000	-0.003044000
H	-9.987197000	-0.905680000	1.950175000
H	-9.986450000	0.880879000	-1.961428000
C	7.304564000	-1.035014000	2.324103000
H	7.997842000	-1.254813000	3.149197000
H	6.561056000	-0.305352000	2.682874000
H	6.746489000	-1.956633000	2.094783000
C	11.672058000	-0.015009000	-0.018427000
H	12.078746000	0.966927000	-0.310737000
H	12.091490000	-0.278108000	0.964167000
H	12.050753000	-0.749373000	-0.750326000
C	7.306060000	1.043231000	-2.319397000
H	6.564634000	0.312956000	-2.681321000
H	6.745194000	1.962084000	-2.086117000
H	7.998937000	1.268323000	-3.143391000
C	-7.306091000	-1.045669000	2.319578000
H	-6.561440000	-0.317868000	2.679730000
H	-7.999472000	-1.266214000	3.144387000
H	-6.749377000	-1.967558000	2.088076000
C	-7.304557000	1.032477000	-2.323908000
H	-7.997340000	1.257051000	-3.148122000
H	-6.742074000	1.950887000	-2.092776000
H	-6.564498000	0.300182000	-2.684592000

C	-11.672097000	0.021659000	0.003177000
H	-12.090919000	-0.707257000	0.713588000
H	-12.046048000	1.018652000	0.295886000
H	-12.084228000	-0.196977000	-0.994684000

2M’: E= -2720.042179

C	3.067135000	2.480518000	-0.376438000
C	4.326441000	3.095790000	-0.477934000
C	5.547516000	2.395115000	-0.372206000
C	5.506970000	1.008559000	-0.144701000
C	4.208655000	0.421140000	-0.065903000
C	2.982739000	1.077782000	-0.164979000
H	2.167962000	3.093036000	-0.467528000
H	4.360234000	4.174730000	-0.650047000
H	6.495696000	2.928861000	-0.472672000
C	6.477106000	-0.057526000	0.001008000
C	5.728457000	-1.294324000	0.167435000
C	6.034303000	-2.643716000	0.390487000
C	4.967869000	-3.567844000	0.529722000
C	3.617987000	-3.203046000	0.466642000
C	3.268062000	-1.837063000	0.259668000
C	4.343115000	-0.960537000	0.124742000
H	7.068244000	-2.990328000	0.462459000
H	5.213160000	-4.619741000	0.697291000
H	2.843866000	-3.965883000	0.581573000
C	1.822779000	0.207659000	-0.036767000
S	-0.444319000	2.031904000	-0.299709000
C	1.980887000	-1.191910000	0.169915000
C	-1.980878000	1.191861000	-0.169632000
C	-0.432888000	-0.539532000	0.081916000
C	-1.822773000	-0.207703000	0.037102000
S	0.444331000	-2.031936000	0.300128000
C	-2.982737000	-1.077827000	0.165307000
C	-3.268050000	1.837005000	-0.259459000
C	-4.343106000	0.960487000	-0.124474000
C	-4.208654000	-0.421181000	0.066240000
C	-5.728442000	1.294251000	-0.167359000
C	-6.034298000	2.643612000	-0.390563000
H	-7.068244000	2.990192000	-0.462641000
C	-3.617980000	3.202966000	-0.466583000
H	-2.843857000	3.965801000	-0.581533000
C	-3.067132000	-2.480575000	0.376678000
H	-2.167958000	-3.093095000	0.467753000
C	-5.506976000	-1.008619000	0.144899000
C	-6.477104000	0.057431000	-0.000968000
C	-5.547514000	-2.395195000	0.372330000
H	-6.495695000	-2.928954000	0.472724000

C	-4.326443000	-3.095863000	0.478093000
H	-4.360235000	-4.174812000	0.650150000
C	-4.967858000	3.567747000	-0.529785000
H	-5.213147000	4.619629000	-0.697455000
C	0.432894000	0.539488000	-0.081576000
C	7.940858000	0.083324000	-0.008632000
C	8.592203000	0.829730000	1.008091000
C	8.710836000	-0.520913000	-1.036856000
C	9.986214000	0.951227000	0.976732000
C	10.101194000	-0.364254000	-1.028200000
C	10.761992000	0.363611000	-0.030159000
H	10.483956000	1.515519000	1.771471000
H	10.687295000	-0.814028000	-1.835727000
C	-7.940871000	-0.083319000	0.008439000
C	-8.592116000	-0.829614000	-1.008385000
C	-8.710953000	0.520940000	1.036593000
C	-9.986156000	-0.950984000	-0.977267000
C	-10.101311000	0.364362000	1.027747000
C	-10.762031000	-0.363383000	0.029553000
H	-10.483818000	-1.515140000	-1.772148000
H	-10.687498000	0.814162000	1.835200000
C	8.048793000	-1.276998000	-2.165741000
H	8.756523000	-1.461117000	-2.987325000
H	7.660061000	-2.252690000	-1.834121000
H	7.187324000	-0.721247000	-2.567758000
C	7.812107000	1.449859000	2.144021000
H	7.073211000	0.743842000	2.553998000
H	8.481326000	1.763361000	2.958682000
H	7.244792000	2.335403000	1.817101000
C	12.265988000	0.482500000	-0.024078000
H	12.599113000	1.400475000	0.484131000
H	12.726131000	-0.369983000	0.506457000
H	12.675932000	0.487620000	-1.046055000
C	-7.811852000	-1.449874000	-2.144125000
H	-7.072512000	-0.744116000	-2.553769000
H	-7.245055000	-2.335712000	-1.817108000
H	-8.480911000	-1.763050000	-2.959045000
C	-8.048986000	1.276945000	2.165572000
H	-8.757094000	1.462116000	2.986592000
H	-7.188334000	0.720525000	2.568431000
H	-7.659029000	2.252079000	1.833763000
C	-12.266039000	-0.482132000	0.023354000
H	-12.599329000	-1.398852000	-0.486993000
H	-12.675842000	-0.489659000	1.045383000
H	-12.726175000	0.371695000	-0.505019000

2M’ (triplet): E= -2720.073656

C	3.065415000	-2.486478000	0.336986000
C	4.320144000	-3.104229000	0.425287000
C	5.545261000	-2.400275000	0.329069000

C	5.500010000	-1.015600000	0.129435000
C	4.208737000	-0.424315000	0.054564000
C	2.982708000	-1.079137000	0.145032000
H	2.164528000	-3.097585000	0.420365000
H	4.353793000	-4.186128000	0.577881000
H	6.494674000	-2.934865000	0.412977000
C	6.477068000	0.060054000	-0.007626000
C	5.726390000	1.302580000	-0.163713000
C	6.037293000	2.653690000	-0.354301000
C	4.969742000	3.578804000	-0.477471000
C	3.620432000	3.210800000	-0.424448000
C	3.269973000	1.843105000	-0.241623000
C	4.345553000	0.964927000	-0.123317000
H	7.072385000	2.999472000	-0.410785000
H	5.213014000	4.634562000	-0.622757000
H	2.845879000	3.975179000	-0.526041000
C	1.823483000	-0.206440000	0.029570000
S	-0.445181000	-2.035304000	0.271425000
C	1.981569000	1.193602000	-0.157338000
C	-1.981565000	-1.193567000	0.157340000
C	-0.432669000	0.540453000	-0.072865000
C	-1.823479000	0.206470000	-0.029609000
S	0.445186000	2.035328000	-0.271516000
C	-2.982706000	1.079166000	-0.145073000
C	-3.269968000	-1.843066000	0.241666000
C	-4.345548000	-0.964888000	0.123363000
C	-4.208734000	0.424349000	-0.054558000
C	-5.726385000	-1.302542000	0.163777000
C	-6.037287000	-2.653649000	0.354380000
H	-7.072378000	-2.999431000	0.410875000
C	-3.620425000	-3.210760000	0.424502000
H	-2.845871000	-3.975139000	0.526086000
C	-3.065415000	2.486500000	-0.337069000
H	-2.164528000	3.097603000	-0.420483000
C	-5.500009000	1.015632000	-0.129431000
C	-6.477065000	-0.060018000	0.007679000
C	-5.545261000	2.400299000	-0.329121000
H	-6.494674000	2.934886000	-0.413041000
C	-4.320145000	3.104248000	-0.425377000
H	-4.353795000	4.186142000	-0.578010000
C	-4.969734000	-3.578763000	0.477545000
H	-5.213006000	-4.634520000	0.622840000
C	0.432672000	-0.540425000	0.072810000
C	7.945065000	-0.082351000	0.006105000
C	8.603839000	-0.737156000	-1.063138000
C	8.697288000	0.429329000	1.090821000
C	9.997359000	-0.864144000	-1.026719000
C	10.089054000	0.275871000	1.085316000
C	10.760498000	-0.364325000	0.036354000
H	10.503740000	-1.363712000	-1.858470000
H	10.665402000	0.659940000	1.932647000

C	-7.945063000	0.082371000	-0.006064000
C	-8.603862000	0.737203000	1.063137000
C	-8.697268000	-0.429397000	-1.090762000
C	-9.997397000	0.864141000	1.026703000
C	-10.089029000	-0.275991000	-1.085271000
C	-10.760507000	0.364239000	-0.036339000
H	-10.503796000	1.363727000	1.858430000
H	-10.665362000	-0.660128000	-1.932583000
C	8.010813000	1.095753000	2.260220000
H	8.718928000	1.296183000	3.077500000
H	7.549123000	2.052680000	1.969506000
H	7.196903000	0.466969000	2.655246000
C	7.823015000	-1.262710000	-2.244705000
H	7.162113000	-0.486095000	-2.661972000
H	8.493735000	-1.609830000	-3.044193000
H	7.170584000	-2.103105000	-1.960056000
C	12.264828000	-0.484866000	0.034136000
H	12.597165000	-1.402548000	-0.475651000
H	12.727264000	0.366821000	-0.495474000
H	12.672988000	-0.493438000	1.056506000
C	-7.823080000	1.262828000	2.244700000
H	-7.162134000	0.486264000	2.661991000
H	-7.170700000	2.103259000	1.960041000
H	-8.493829000	1.609923000	3.044174000
C	-8.010755000	-1.095875000	-2.260107000
H	-8.718852000	-1.296380000	-3.077384000
H	-7.196860000	-0.467091000	-2.655160000
H	-7.549037000	-2.052769000	-1.969325000
C	-12.264849000	0.484640000	-0.034200000
H	-12.597403000	1.401342000	0.477191000
H	-12.672784000	0.495040000	-1.056650000
H	-12.727312000	-0.368096000	0.493699000

3M: E= -4956.247235

C	-2.710416000	2.826327000	-0.123734000
C	-4.007256000	3.357068000	-0.106400000
C	-5.151353000	2.512233000	-0.101054000
C	-4.984790000	1.128241000	-0.112741000
C	-3.653066000	0.651300000	-0.130584000
C	-2.498567000	1.427125000	-0.136828000
H	-1.864924000	3.517884000	-0.127723000
H	-6.135064000	2.984444000	-0.088916000
C	-5.869629000	-0.071576000	-0.112039000
C	-4.967974000	-1.257995000	-0.133534000
C	-5.118722000	-2.643359000	-0.144958000
C	-3.963606000	-3.473193000	-0.163785000
C	-2.673521000	-2.925530000	-0.171976000
C	-2.479114000	-1.523716000	-0.161609000
C	-3.643369000	-0.763040000	-0.142800000

H	-6.097325000	-3.126244000	-0.140641000
H	-1.819299000	-3.606137000	-0.187427000
C	-1.227642000	0.683478000	-0.156873000
C	0.002939000	1.351831000	-0.165744000
C	-1.218107000	-0.763312000	-0.168859000
C	1.242276000	0.700176000	-0.185974000
C	0.021295000	-1.414969000	-0.188411000
C	1.251845000	-0.746591000	-0.197813000
H	-0.004291000	2.441752000	-0.157171000
H	0.028424000	-2.504881000	-0.197418000
C	2.522891000	-1.489928000	-0.220706000
C	2.503208000	1.460751000	-0.196596000
C	3.667873000	0.700633000	-0.216939000
C	3.677287000	-0.714046000	-0.228530000
C	4.992162000	1.196010000	-0.233260000
C	5.141833000	2.581455000	-0.231231000
H	6.120038000	3.064761000	-0.248941000
C	2.697032000	2.862684000	-0.191410000
H	1.842438000	3.542847000	-0.176860000
C	2.735520000	-2.889031000	-0.238519000
H	1.890184000	-3.580793000	-0.235111000
C	5.008061000	-1.191308000	-0.252921000
C	5.894450000	0.008351000	-0.247875000
C	5.176321000	-2.574460000	-0.273771000
H	6.160956000	-3.044126000	-0.299457000
C	4.032326000	-3.419685000	-0.264298000
C	3.986614000	3.411000000	-0.208111000
C	-4.159164000	-4.971981000	-0.176888000
H	-4.733755000	-5.290658000	-1.063100000
H	-3.200214000	-5.510856000	-0.186666000
H	-4.725301000	-5.307607000	0.708506000
C	-4.221804000	4.853415000	-0.094643000
H	-4.799615000	5.180131000	-0.975963000
H	-4.791634000	5.167697000	0.796326000
H	-3.269640000	5.404236000	-0.095163000
C	4.181457000	4.909979000	-0.205602000
H	4.761758000	5.235024000	0.674664000
H	4.741010000	5.240301000	-1.097147000
H	3.222278000	5.448362000	-0.192644000
C	4.247052000	-4.915787000	-0.286323000
H	4.831182000	-5.247548000	0.588890000
H	3.295065000	-5.466897000	-0.282859000
H	4.811332000	-5.223949000	-1.182804000
C	-7.239246000	-0.091138000	-0.092461000
C	7.262789000	0.017378000	-0.244399000
S	-8.219891000	-1.559414000	-0.111977000
S	-8.223236000	1.388082000	-0.064996000
C	-9.799042000	-0.761667000	-0.023223000
C	-9.800133000	0.596722000	-0.001413000
S	8.263250000	-1.444477000	-0.295758000
S	8.243723000	1.493108000	-0.272110000
C	9.824357000	-0.647200000	-0.031701000

C	9.815376000	0.712489000	-0.020777000
S	-11.291219000	1.545722000	0.064224000
S	-11.244539000	-1.775436000	0.041112000
C	-11.410536000	-1.955471000	1.860349000
H	-10.508642000	-2.419492000	2.283909000
H	-11.589716000	-0.974216000	2.322052000
H	-12.277370000	-2.609876000	2.034451000
C	-10.677135000	3.221793000	0.452466000
H	-10.107821000	3.237272000	1.392827000
H	-10.071066000	3.633947000	-0.367670000
H	-11.581452000	3.836280000	0.567548000
S	11.259293000	-1.669308000	0.088043000
S	11.237137000	1.750856000	0.114589000
C	10.871412000	-2.628785000	1.602560000
H	10.806179000	-1.964304000	2.475877000
H	9.937037000	-3.196456000	1.490076000
H	11.707920000	-3.329999000	1.736363000
C	10.834223000	2.687881000	1.639144000
H	9.894591000	3.247567000	1.530434000
H	10.772819000	2.012302000	2.504185000
H	11.663190000	3.395768000	1.784281000

3M(triplet): E= -4956.183043

C	-2.662596000	2.780822000	-0.122040000
C	-3.947145000	3.336371000	-0.192595000
C	-5.107722000	2.514419000	-0.208855000
C	-4.970547000	1.128287000	-0.153172000
C	-3.649894000	0.625726000	-0.084078000
C	-2.480459000	1.378532000	-0.065612000
H	-1.803460000	3.455272000	-0.113168000
H	-6.080393000	3.005728000	-0.266945000
C	-5.879444000	-0.053231000	-0.146914000
C	-5.003135000	-1.256384000	-0.075017000
C	-5.182309000	-2.637965000	-0.039589000
C	-4.045438000	-3.489984000	0.029814000
C	-2.745130000	-2.968028000	0.062414000
C	-2.521915000	-1.570907000	0.027713000
C	-3.669401000	-0.787747000	-0.038635000
H	-6.169997000	-3.101269000	-0.064392000
H	-1.905905000	-3.665269000	0.114411000
C	-1.226002000	0.610608000	0.006120000
C	0.016958000	1.255559000	0.031866000
C	-1.246117000	-0.835808000	0.050330000
C	1.242364000	0.581369000	0.095574000
C	-0.021267000	-1.512175000	0.113233000
C	1.222362000	-0.869359000	0.136720000
H	0.032614000	2.345153000	-0.000201000
H	-0.035998000	-2.601818000	0.144827000
C	2.477435000	-1.636948000	0.200816000
C	2.517840000	1.316990000	0.118839000
C	3.654543000	0.530727000	0.177525000

C	3.634921000	-0.879668000	0.217032000
C	4.999600000	0.984246000	0.203105000
C	5.203766000	2.366306000	0.168469000
H	6.211425000	2.789788000	0.186051000
C	2.764224000	2.718765000	0.087672000
H	1.933568000	3.427036000	0.042721000
C	2.685458000	-3.044345000	0.251007000
H	1.835864000	-3.731162000	0.241604000
C	4.967130000	-1.366909000	0.282068000
C	5.848860000	-0.204287000	0.275833000
C	5.132750000	-2.753860000	0.331613000
H	6.128943000	-3.202311000	0.384577000
C	3.980398000	-3.587617000	0.314749000
C	4.073370000	3.228865000	0.113163000
C	-4.271608000	-4.984065000	0.067251000
H	-4.824436000	-5.325471000	-0.824223000
H	-3.324206000	-5.541464000	0.109220000
H	-4.872260000	-5.274042000	0.946074000
C	-4.130651000	4.835570000	-0.254039000
H	-4.670752000	5.133709000	-1.168669000
H	-4.724319000	5.199905000	0.601640000
H	-3.167930000	5.367620000	-0.244700000
C	4.309919000	4.721652000	0.079079000
H	4.864977000	5.056453000	0.971571000
H	4.913481000	5.008854000	-0.798448000
H	3.366755000	5.286303000	0.038023000
C	4.175132000	-5.085934000	0.369310000
H	4.719498000	-5.383349000	1.281433000
H	3.216601000	-5.625538000	0.359321000
H	4.770589000	-5.441543000	-0.488581000
C	-7.248632000	-0.045625000	-0.194768000
C	7.304978000	-0.238589000	0.296474000
S	-8.258144000	-1.494272000	-0.198980000
S	-8.201076000	1.451658000	-0.278242000
C	-9.822330000	-0.663824000	-0.223266000
C	-9.795690000	0.693947000	-0.260461000
S	8.184600000	-0.629496000	-1.196131000
S	8.237514000	0.708954000	1.451532000
C	9.719773000	0.076483000	-0.695568000
C	9.747876000	0.679786000	0.529221000
S	-11.267036000	1.673430000	-0.318540000
S	-11.289797000	-1.647847000	-0.186129000
C	-11.574441000	-1.700845000	1.626335000
H	-10.712966000	-2.153277000	2.137415000
H	-11.758459000	-0.686725000	2.008223000
H	-12.466643000	-2.323858000	1.786907000
C	-10.644577000	3.345709000	0.073237000
H	-10.135066000	3.370241000	1.047075000
H	-9.980053000	3.728518000	-0.715220000
H	-11.541509000	3.979832000	0.118479000
S	11.152647000	0.008117000	-1.732059000
S	11.189823000	1.416778000	1.240637000

C	10.613654000	-1.111251000	-3.070181000
H	10.326058000	-2.098550000	-2.680974000
H	9.786437000	-0.677003000	-3.650481000
H	11.490230000	-1.220510000	-3.724604000
C	11.937746000	-0.067898000	2.018153000
H	11.246350000	-0.501219000	2.754608000
H	12.192467000	-0.809738000	1.247917000
H	12.854361000	0.267639000	2.525411000

3M·+: E= -4956.030627

C	-2.704811000	2.807797000	-0.011199000
C	-3.997339000	3.349637000	0.000455000
C	-5.144418000	2.509158000	-0.017939000
C	-4.985277000	1.121894000	-0.048495000
C	-3.656715000	0.637439000	-0.058727000
C	-2.498958000	1.404402000	-0.041916000
H	-1.855788000	3.494358000	0.003501000
H	-6.124534000	2.988538000	-0.008199000
C	-5.873328000	-0.069101000	-0.075957000
C	-4.984204000	-1.259176000	-0.103601000
C	-5.145081000	-2.645781000	-0.138295000
C	-3.997903000	-3.486455000	-0.158554000
C	-2.705231000	-2.944777000	-0.144132000
C	-2.498842000	-1.541445000	-0.109673000
C	-3.656364000	-0.774341000	-0.091316000
H	-6.125915000	-3.123644000	-0.152192000
H	-1.856410000	-3.631527000	-0.160976000
C	-1.233780000	0.657125000	-0.058846000
C	0.000003000	1.318144000	-0.043880000
C	-1.233773000	-0.794218000	-0.092077000
C	1.233787000	0.657125000	-0.058827000
C	0.000004000	-1.455203000	-0.107021000
C	1.233780000	-0.794218000	-0.092057000
H	0.000003000	2.407549000	-0.019455000
H	0.000004000	-2.544574000	-0.131512000
C	2.498850000	-1.541446000	-0.109636000
C	2.498963000	1.404402000	-0.041886000
C	3.656722000	0.637440000	-0.058699000
C	3.656371000	-0.774340000	-0.091273000
C	4.985283000	1.121898000	-0.048512000
C	5.144421000	2.509162000	-0.017982000
H	6.124535000	2.988548000	-0.008292000
C	2.704815000	2.807799000	-0.011190000
H	1.855790000	3.494358000	0.003491000
C	2.705240000	-2.944776000	-0.144120000
H	1.856422000	-3.631527000	-0.160986000
C	4.984212000	-1.259173000	-0.103585000
C	5.873336000	-0.069099000	-0.075970000
C	5.145091000	-2.645778000	-0.138330000
H	6.125927000	-3.123634000	-0.152310000
C	3.997915000	-3.486452000	-0.158561000

C	3.997340000	3.349640000	0.000451000
C	-4.204581000	-4.982233000	-0.197212000
H	-4.773947000	-5.280375000	-1.093194000
H	-3.250375000	-5.528201000	-0.208129000
H	-4.779680000	-5.325731000	0.678644000
C	-4.203467000	4.845843000	0.031850000
H	-4.771327000	5.186546000	-0.849930000
H	-4.778352000	5.148720000	0.922818000
H	-3.248977000	5.391214000	0.047168000
C	4.203470000	4.845838000	0.032175000
H	4.775865000	5.148780000	0.924738000
H	4.773823000	5.186411000	-0.848029000
H	3.248973000	5.391268000	0.044800000
C	4.204593000	-4.982239000	-0.196898000
H	4.777074000	-5.325895000	0.680627000
H	3.250383000	-5.528134000	-0.210657000
H	4.776588000	-5.280305000	-1.091214000
C	-7.257410000	-0.077834000	-0.075122000
C	7.257417000	-0.077841000	-0.075166000
S	-8.235288000	-1.527887000	-0.106833000
S	-8.224031000	1.397378000	-0.040415000
C	-9.806413000	-0.742506000	-0.102071000
C	-9.803176000	0.626174000	-0.075924000
S	8.235287000	-1.527902000	-0.106681000
S	8.224049000	1.397378000	-0.040721000
C	9.806418000	-0.742527000	-0.102036000
C	9.803186000	0.626158000	-0.076047000
S	-11.291302000	1.561599000	-0.079137000
S	-11.253223000	-1.754626000	-0.144239000
C	-11.676155000	-1.777926000	1.643765000
H	-10.872519000	-2.250597000	2.225012000
H	-11.870482000	-0.758478000	2.004570000
H	-12.593535000	-2.378238000	1.728529000
C	-10.687074000	3.275765000	0.085933000
H	-10.132559000	3.420824000	1.024519000
H	-10.075043000	3.571987000	-0.778598000
H	-11.595219000	3.894462000	0.107001000
S	11.253228000	-1.754657000	-0.144027000
S	11.291322000	1.561580000	-0.079289000
C	11.675960000	-1.777849000	1.644022000
H	11.870129000	-0.758364000	2.004811000
H	10.872305000	-2.250586000	2.225192000
H	12.593395000	-2.378055000	1.728921000
C	10.687103000	3.275767000	0.085586000
H	10.075173000	3.571930000	-0.779039000
H	10.132478000	3.420897000	1.024097000
H	11.595250000	3.894458000	0.106724000

$3M^{2+}$: E= -4955.743035

C	-2.706449000	2.822383000	0.016417000
C	-4.001736000	3.357963000	0.019182000

C	-5.141458000	2.506646000	0.001660000
C	-4.966558000	1.120432000	-0.021355000
C	-3.635186000	0.644817000	-0.018681000
C	-2.485165000	1.418103000	-0.001867000
H	-1.863427000	3.516085000	0.030137000
H	-6.124875000	2.979232000	0.010090000
C	-5.841528000	-0.078071000	-0.039722000
C	-4.948131000	-1.264292000	-0.047005000
C	-5.103038000	-2.652318000	-0.075262000
C	-3.950249000	-3.487016000	-0.083936000
C	-2.663384000	-2.932583000	-0.067008000
C	-2.463291000	-1.524945000	-0.043138000
C	-3.624585000	-0.768785000	-0.034756000
H	-6.079191000	-3.139433000	-0.095588000
H	-1.810024000	-3.613582000	-0.074904000
C	-1.213128000	0.680606000	-0.005745000
C	0.015762000	1.351968000	0.011074000
C	-1.202601000	-0.768747000	-0.028345000
C	1.253517000	0.698129000	0.005399000
C	0.036008000	-1.422248000	-0.035874000
C	1.264008000	-0.750617000	-0.021233000
H	0.008226000	2.441419000	0.029101000
H	0.044200000	-2.511659000	-0.054005000
C	2.536487000	-1.490098000	-0.032514000
C	2.515119000	1.455755000	0.025535000
C	3.677006000	0.698569000	0.014881000
C	3.687253000	-0.716327000	-0.014094000
C	4.999261000	1.195426000	0.030161000
C	5.153114000	2.581421000	0.059373000
H	6.128751000	3.069967000	0.073152000
C	2.713285000	2.861042000	0.055229000
H	1.859816000	3.541891000	0.065929000
C	2.755007000	-2.892402000	-0.060496000
H	1.911562000	-3.585506000	-0.076908000
C	5.016549000	-1.194167000	-0.020190000
C	5.896878000	0.006937000	0.007979000
C	5.190528000	-2.577847000	-0.047984000
H	6.173209000	-3.052184000	-0.054936000
C	4.049680000	-3.428372000	-0.068332000
C	4.000030000	3.415479000	0.072076000
C	-4.146181000	-4.983528000	-0.113393000
H	-4.712886000	-5.290042000	-1.007866000
H	-3.188653000	-5.522767000	-0.120864000
H	-4.718398000	-5.324564000	0.764942000
C	-4.218709000	4.851948000	0.041049000
H	-4.782797000	5.182694000	-0.846536000
H	-4.800660000	5.154271000	0.927202000
H	-3.268790000	5.404220000	0.059307000
C	4.194825000	4.912296000	0.104102000
H	4.759627000	5.218703000	0.999983000
H	4.766819000	5.255762000	-0.773586000
H	3.236885000	5.450882000	0.111491000

C	4.265884000	-4.922288000	-0.098560000
H	4.836748000	-5.257396000	0.783085000
H	3.315710000	-5.474307000	-0.112205000
H	4.840961000	-5.220885000	-0.990531000
C	-7.238017000	-0.094300000	-0.050808000
C	7.281839000	0.016811000	0.012673000
S	-8.195707000	-1.541689000	0.024229000
S	-8.211561000	1.362841000	-0.128725000
C	-9.763769000	-0.799070000	-0.072224000
C	-9.775750000	0.584279000	-0.156290000
S	8.260544000	-1.436626000	-0.021022000
S	8.239456000	1.484048000	0.052847000
C	9.819285000	-0.664023000	0.003356000
C	9.809199000	0.733768000	0.038655000
S	-11.265119000	1.476030000	-0.316540000
S	-11.178277000	-1.852862000	-0.125078000
C	-11.863704000	-1.593185000	1.562164000
H	-11.158733000	-1.956230000	2.321855000
H	-12.116436000	-0.537551000	1.728194000
H	-12.782007000	-2.197031000	1.592076000
C	-10.700341000	3.209515000	-0.354741000
H	-10.190259000	3.485636000	0.579822000
H	-10.059792000	3.398518000	-1.228885000
H	-11.620274000	3.803138000	-0.450618000
S	11.310830000	-1.564916000	-0.013962000
S	11.287553000	1.655963000	0.065745000
C	10.748771000	-3.297131000	-0.068502000
H	10.167686000	-3.552216000	0.829883000
H	10.176186000	-3.497538000	-0.985997000
H	11.672680000	-3.892031000	-0.082006000
C	10.700202000	3.379913000	0.114028000
H	10.121735000	3.625392000	-0.788712000
H	10.118414000	3.573226000	1.027250000
H	11.615284000	3.988153000	0.133115000

4M: E= -5751.225422

C	3.072669000	2.423172000	0.100009000
C	4.319135000	3.060947000	0.084060000
C	5.535530000	2.318779000	0.081257000
C	5.495745000	0.926404000	0.094221000
C	4.210910000	0.332113000	0.110483000
C	2.989203000	1.009347000	0.114302000
H	2.168043000	3.034789000	0.102081000
H	6.472352000	2.878523000	0.069809000
C	6.482413000	-0.192384000	0.096328000
C	5.688310000	-1.454162000	0.117039000
C	5.953386000	-2.821195000	0.129974000
C	4.871685000	-3.751291000	0.148431000
C	3.539810000	-3.323706000	0.154904000
C	3.231207000	-1.940321000	0.142749000
C	4.326063000	-1.072124000	0.124071000

H	6.969040000	-3.220135000	0.127232000
H	2.737516000	-4.066398000	0.170101000
C	1.818087000	0.147116000	0.133703000
S	-0.423676000	2.027242000	0.136788000
C	1.954910000	-1.264939000	0.147369000
C	-1.972671000	1.200367000	0.163082000
C	-0.450113000	-0.570567000	0.164801000
C	-1.835832000	-0.211666000	0.176905000
S	0.405946000	-2.091885000	0.172037000
C	-3.007059000	-1.073645000	0.202083000
C	-3.248844000	1.875849000	0.172166000
C	-4.344146000	1.008226000	0.195489000
C	-4.228628000	-0.396316000	0.209879000
C	-5.706066000	1.390712000	0.211455000
C	-5.970138000	2.757932000	0.204928000
H	-6.985450000	3.157351000	0.220632000
C	-3.556901000	3.259417000	0.163253000
H	-2.754248000	4.001695000	0.146245000
C	-3.091228000	-2.487437000	0.222240000
H	-2.186732000	-3.099259000	0.218857000
C	-5.512430000	-0.990867000	0.236304000
C	-6.500626000	0.127609000	0.230479000
C	-5.553935000	-2.382507000	0.259168000
H	-6.491869000	-2.939781000	0.286175000
C	-4.337605000	-3.125101000	0.249918000
C	-4.888324000	3.687697000	0.178978000
C	0.432302000	0.505888000	0.143782000
C	-4.422762000	-4.633882000	0.274921000
H	-3.427155000	-5.101162000	0.265319000
H	-4.952653000	-4.987091000	1.175765000
H	-4.982836000	-5.015208000	-0.595749000
C	-5.217343000	5.162826000	0.173393000
H	-5.835193000	5.429147000	-0.700934000
H	-5.794466000	5.444341000	1.070502000
H	-4.310991000	5.785385000	0.147290000
C	5.201380000	-5.226170000	0.162079000
H	5.808439000	-5.489174000	1.044844000
H	4.295135000	-5.849226000	0.178549000
H	5.790250000	-5.509826000	-0.726669000
C	4.404095000	4.569998000	0.070428000
H	3.408249000	5.036863000	0.072745000
H	4.954235000	4.945406000	0.950010000
H	4.943103000	4.930287000	-0.822297000
C	-7.864707000	0.017439000	0.231336000
C	7.848763000	-0.092719000	0.080465000
S	-8.733944000	-1.524806000	0.289282000
S	-8.968393000	1.403190000	0.258307000
C	-10.360131000	-0.865845000	0.035174000
C	-10.468731000	0.489452000	0.020308000
S	8.952509000	-1.470032000	0.101516000
S	8.699503000	1.466140000	0.054584000
C	10.456817000	-0.538275000	0.025179000

C	10.339835000	0.815108000	0.003851000
S	-11.700284000	-2.010501000	-0.075006000
S	-11.976322000	1.399783000	-0.107580000
C	-11.260140000	-2.900182000	-1.617660000
H	-11.284111000	-2.216683000	-2.478307000
H	-10.271142000	-3.373147000	-1.538905000
H	-12.025859000	-3.679087000	-1.745520000
C	-11.656748000	2.385881000	-1.620807000
H	-10.774424000	3.030453000	-1.502812000
H	-11.529646000	1.728402000	-2.492586000
H	-12.548043000	3.014337000	-1.762103000
S	11.985347000	-1.423186000	-0.028982000
S	11.743576000	1.889553000	-0.047667000
C	12.201767000	-1.547343000	-1.847566000
H	11.354587000	-2.082193000	-2.299536000
H	12.299658000	-0.543693000	-2.284879000
H	13.128201000	-2.115888000	-2.016173000
C	10.991278000	3.505666000	-0.446738000
H	11.840379000	4.195590000	-0.554580000
H	10.432511000	3.470048000	-1.392844000
H	10.343769000	3.865591000	0.366253000

4M(triplet): E= -5751.147423

C	-3.068799000	2.438207000	-0.097955000
C	-4.322696000	3.076286000	-0.083564000
C	-5.527587000	2.330005000	-0.081811000
C	-5.478853000	0.931557000	-0.094324000
C	-4.195465000	0.344311000	-0.109042000
C	-2.979539000	1.030093000	-0.111641000
H	-2.167267000	3.054143000	-0.099192000
H	-6.469104000	2.881769000	-0.071534000
C	-6.461595000	-0.189447000	-0.097112000
C	-5.662195000	-1.447865000	-0.116342000
C	-5.933960000	-2.825336000	-0.128546000
C	-4.853565000	-3.756624000	-0.145604000
C	-3.522146000	-3.334286000	-0.151206000
C	-3.203401000	-1.941057000	-0.139473000
C	-4.311293000	-1.067787000	-0.122226000
H	-6.950536000	-3.220812000	-0.126107000
H	-2.720708000	-4.077560000	-0.165232000
C	-1.792133000	0.174909000	-0.129393000
S	0.399952000	2.064194000	-0.129989000
C	-1.949202000	-1.290967000	-0.143042000
C	1.966688000	1.224549000	-0.156434000
C	0.472684000	-0.586269000	-0.158582000
C	1.809631000	-0.241584000	-0.170366000
S	-0.382431000	-2.130602000	-0.165728000
C	2.997199000	-1.096163000	-0.195459000
C	3.220502000	1.875185000	-0.165401000
C	4.329019000	1.002661000	-0.189146000
C	4.212773000	-0.410066000	-0.203534000

C	5.679346000	1.383357000	-0.204353000
C	5.950113000	2.761345000	-0.196021000
H	6.966329000	3.157362000	-0.209466000
C	3.538396000	3.268680000	-0.155816000
H	2.736529000	4.011429000	-0.138661000
C	3.087549000	-2.504311000	-0.214763000
H	2.186308000	-3.120706000	-0.211184000
C	5.495137000	-0.997248000	-0.229440000
C	6.479242000	0.123869000	-0.224821000
C	5.546127000	-2.394892000	-0.251263000
H	6.488935000	-2.943856000	-0.277193000
C	4.341431000	-3.141914000	-0.241867000
C	4.869491000	3.691827000	-0.170534000
C	-0.455111000	0.519627000	-0.137654000
C	4.426727000	-4.650936000	-0.266104000
H	3.430940000	-5.117757000	-0.252339000
H	4.952136000	-5.005603000	-1.168937000
H	4.989256000	-5.032505000	0.602771000
C	5.201891000	5.166328000	-0.163835000
H	5.821328000	5.429525000	0.710273000
H	5.779252000	5.446453000	-1.061194000
H	4.297147000	5.791097000	-0.136472000
C	-5.187088000	-5.230746000	-0.158896000
H	-5.792155000	-5.492115000	-1.043485000
H	-4.282579000	-5.856319000	-0.171778000
H	-5.779965000	-5.511102000	0.728181000
C	-4.407369000	4.585600000	-0.071438000
H	-3.411110000	5.051585000	-0.067133000
H	-4.950115000	4.961479000	-0.955350000
H	-4.951554000	4.947593000	0.817333000
C	7.844159000	0.016316000	-0.230377000
C	-7.828894000	-0.092403000	-0.083083000
S	8.717660000	-1.523575000	-0.291832000
S	8.945988000	1.403804000	-0.262385000
C	10.343297000	-0.861589000	-0.042717000
C	10.448894000	0.493906000	-0.028474000
S	-8.931289000	-1.470795000	-0.106071000
S	-8.683526000	1.464523000	-0.058066000
C	-10.437291000	-0.542318000	-0.030631000
C	-10.322895000	0.811235000	-0.008918000
S	11.685175000	-2.004424000	0.064595000
S	11.955372000	1.406761000	0.093607000
C	11.256847000	-2.884053000	1.616352000
H	11.289397000	-2.195331000	2.472538000
H	10.266226000	-3.355328000	1.548623000
H	12.022034000	-3.663762000	1.742506000
C	11.634429000	2.404906000	1.598586000
H	10.754666000	3.051658000	1.473706000
H	11.503052000	1.754326000	2.474889000
H	12.527462000	3.031419000	1.737502000
S	-11.964020000	-1.430509000	0.021559000
S	-11.728171000	1.883607000	0.040962000

C	-12.182156000	-1.555725000	1.839844000
H	-11.334397000	-2.088958000	2.292631000
H	-12.282767000	-0.552456000	2.277407000
H	-13.107564000	-2.126331000	2.007084000
C	-10.979068000	3.500168000	0.444277000
H	-11.829445000	4.188559000	0.551839000
H	-10.421976000	3.463701000	1.391338000
H	-10.330705000	3.862607000	-0.366918000

References

[56] S. L. Broman, C. L. Andersen, T. Jousselin-Oba, M. Mansø, O. Hammerich, M. Frigoli, M. B. Nielsen, *Org. Biomol. Chem.* **2017**, *15*, 807–811.

[57] L. Broløs, M. D. Kilde, O. Hammerich, M. B. Nielsen, *J. Org. Chem.* **2020**, *85*, 3277–3286.

[58] V. Bliksted Roug Pedersen, J. Granhøj, A. Erbs Hillers-Bendtsen, A. Kadziola, K. V. Mikkelsen, M. Brøndsted Nielsen, *Chem. Eur. J.* **2021**, *27*, 8315–8324.

[59] M. J. Frisch, G. W. Trucks, H. B. Schlegel, G. E. Scuseria, M. A. Robb, J. R. Cheeseman, G. Scalmani, V. Barone, G. Petersson, H. Nakatsuji, *Gaussian09, Revision D* **2016**, *1*.

[60] A. D. Becke, *J. Chem. Phys.* **1993**, *98*, 5648–5652.

[61] C. Lee, W. Yang, R. G. Parr, *Phys. Rev. B* **1988**, *37*, 785–789.

[62] S. H. Vosko, L. Wilk, M. Nusair, *Can. J. Phys.* **1980**, *58*, 1200–1211.

[63] S. Grimme, J. Antony, S. Ehrlich, H. Krieg, *J. Chem. Phys.* **2010**, *132*, 154104.

[64] F. Weigend, R. Ahlrichs, *Phys. Chem. Chem. Phys.* **2005**, *7*, 3297–3305.

[65] J. W. McIver, A. Komornicki, *J. Am. Chem. Soc.* **1972**, *94*, 2625–2633.

[66] Z. Zhou, J. M. Fernández-García, Y. Zhu, P. J. Evans, R. Rodríguez, J. Crassous, Z. Wei, I. Fernández, M. A. Petrukhina, N. Martín, *Angew. Chem. Int. Ed.* **2022**, *134*, e202115747.

[67] Z. Zhou, Y. Zhu, J. M. Fernández-García, Z. Wei, I. Fernández, M. A. Petrukhina, N. Martín, *Chem. Commun.* **2022**, *58*, 5574–5577.

[68] J. M. Seminario, Ed. , *Recent Developments and Applications of Modern Density Functional Theory*, Elsevier, Amsterdam ; New York, **1996**.

[69] D. P. Chong, *Recent Advances in Density Functional Methods*, World Scientific, **1995**.

[70] A. Dreuw, M. Head-Gordon, *Chem. Rev.* **2005**, *105*, 4009–4037.

[71] S. Miertuš, E. Scrocco, J. Tomasi, *Chem. Phys.* **1981**, *55*, 117–129.

[72] J. L. Pascual-ahuir, E. Silla, I. Tuñon, *J. Comput. Chem.* **1994**, *15*, 1127–1138.

[73] V. Barone, M. Cossi, *J. Phys. Chem. A* **1998**, *102*, 1995–2001.

[74] Z. Chen, C. S. Wannere, C. Corminboeuf, R. Puchta, P. von R. Schleyer, *Chem. Rev.* **2005**, *105*, 3842–3888.

[75] K. Wolinski, J. F. Hinton, P. Pulay, *J. Am. Chem. Soc.* **1990**, *112*, 8251–8260.
